# Larvicidal Activities of 2-Aryl-2,3-Dihydroquinazolin -4-ones against Malaria Vector *Anopheles arabiensis*, In Silico ADMET Prediction and Molecular Target Investigation

**DOI:** 10.3390/molecules25061316

**Published:** 2020-03-13

**Authors:** Katharigatta N. Venugopala, Pushpalatha Ramachandra, Christophe Tratrat, Raquel M. Gleiser, Subhrajyoti Bhandary, Deepak Chopra, Mohamed A. Morsy, Bandar E. Aldhubiab, Mahesh Attimarad, Anroop B. Nair, Nagaraja Sreeharsha, Rashmi Venugopala, Pran Kishore Deb, Sandeep Chandrashekharappa, Hany Ezzat Khalil, Osama I. Alwassil, Sara Nidal Abed, Yazan A. Bataineh, Ramachandra Palenge, Michelyne Haroun, Shinu Pottathil, Meravanige B. Girish, Sabah H. Akrawi, Viresh Mohanlall

**Affiliations:** 1 Department of Pharmaceutical Sciences, College of Clinical Pharmacy, King Faisal University, Al-Ahsa 31982, Saudi Arabia; ctratrat@kfu.edu.sa (C.T.); momorsy@kfu.edu.sa (M.A.M.); baldhubiab@kfu.edu.sa (B.E.A.); mattimarad@kfu.edu.sa (M.A.); anair@kfu.edu.sa (A.B.N.); sharsha@kfu.edu.sa (N.S.); heahmed@kfu.edu.sa (H.E.K.); mharoun@kfu.edu.sa (M.H.); sakrawi@kfu.edu.sa (S.H.A.); 2 Department of Biotechnology and Food Technology, Durban University of Technology, Durban 4001, South Africa; vireshm@dut.ac.za; 3 Department of Chemistry, School of Applied Sciences, REVA University, Bangalore 560 064, India; pushpalatha@reva.edu.in (P.R.); ramachandrap@reva.edu.in (R.P.); 4 CREAN-IMBIV (UNC-CONICET), Av. Valparaíso s.n., Córdoba, Argentina and FCEFyN, AV. Sarsfield 299, Universidad Nacional de Cordoba, Cordoba 5000, Argentina; raquel.gleiser@unc.edu.ar; 5 Department of Chemistry, Indian Institute of Science Education and Research Bhopal, Bhopal By-pass Road, Bhauri, Bhopal 462 066, Madhya Pradesh, India; bhandarysj@gmail.com (S.B.); dchopra@iiserb.ac.in (D.C.); 6 Department of Pharmacology, Faculty of Medicine, Minia University, El-Minia 61511, Egypt; 7 Department of Public Health Medicine, University of KwaZulu-Natal, Howard College Campus, Durban 4001, South Africa; rashmivenugopala@gmail.com; 8 Department of Pharmaceutical Sciences, Faculty of Pharmacy, Philadelphia University, P.O. Box 1, Amman 19392, Jordan; pdeb@philadelphia.edu.jo (P.K.D.); sousanidal95@gmail.com (S.N.A.); YBatineh@philadelphia.edu.jo (Y.A.B.); 9 Institute for Stem Cell Biology and Regenerative Medicine, NCBS, TIFR, GKVK, Bellary Road, Bangalore 560 065, India; sandeepc@instem.res.in; 10 Department of Pharmacognosy, Faculty of Pharmacy, Minia University, Minia 61519, Egypt; 11 Department of Pharmaceutical Sciences, College of Pharmacy, King Saud bin Abdulaziz University for Health Sciences, Riyadh 11481, Saudi Arabia; wassilo@ksau-hs.edu.sa; 12 Department of Biomedical Sciences, College of Clinical Pharmacy, King Faisal University, Al-Ahsa 31982, Saudi Arabia; spottathail@kfu.edu.sa; 13 Department of Biomedical Sciences, College of Medicine, King Faisal University, Al-Ahsa 31982, Saudi Arabia; gmeravanige@kfu.edu.sa

**Keywords:** *Anopheles arabiensis*, larvicidal activity, 2,3-dihydroquinazolin-4-one, docking, ADMET, crystallography, graphene oxide

## Abstract

Malaria, affecting all continents, remains one of the life-threatening diseases introduced by parasites that are transmitted to humans through the bites of infected *Anopheles* mosquitoes. Although insecticides are currently used to reduce malaria transmission, their safety concern for living systems, as well as the environment, is a growing problem. Therefore, the discovery of novel, less toxic, and environmentally safe molecules to effectively combat the control of these vectors is in high demand. In order to identify new potential larvicidal agents, a series of 2-aryl-1,2-dihydroquinazolin-4-one derivatives were synthesized and evaluated for their larvicidal activity against *Anopheles arabiensis*. The in silico absorption, distribution, metabolism, excretion, and toxicity (ADMET) properties of the compounds were also investigated and most of the derivatives possessed a favorable ADMET profile. Computational modeling studies of the title compounds demonstrated a favorable binding interaction against the acetylcholinesterase enzyme molecular target. Thus, 2-aryl-1,2-dihydroquinazolin-4-ones were identified as a novel class of *Anopheles arabiensis* insecticides which can be used as lead molecules for the further development of more potent and safer larvicidal agents for treating malaria.

## 1. Introduction

Malaria is considered as one of the challenging life-threatening diseases initiated by parasites that are transmitted to humans through the bites of infected *Anopheles* mosquitoes. Both genders are affected, leading to severe health and socioeconomically negative impacts. Recently, the World Health Organization (WHO) reported in 2018 that there were an estimated 228 million cases of malaria that spread in 87 countries. The mortality rate of malaria was found to be 405,000 in 2018 [1]. The *Anopheles arabiensis* mosquito is considered as one of the major vectors of malaria. The origin of infection starts with a bite from an infected female mosquito, where the parasite is delivered into the circulatory system and ultimately to the liver, where it becomes mature and reproduces [2]. Many approaches and strategies for the management of malaria were developed. One of the most effective strategies is to eliminate the vector through environmental modifications, biological control, use of long-lasting insecticidal nets (LLINs), and indoor residual spraying (IRS). Another effective strategy is to eradicate mosquitos at the larval stage by preventing their development.

Organophosphates and carbamates are two major classes of synthetic insecticides known to inactivate acetylcholinesterase (AChE), and they are currently used in combating malaria. However, their use raises concerns regarding safety issues for the living system and the environment [3]. For instance, the most popular insecticide organophosphorus Temephos is a mutagenic compound and is unsafe for human beings. In addition, insecticide resistance is becoming increasingly alarming and threatens the management of vectors, thereby leading to prioritizing the development of new potent and safer biologically active heterocyclic compounds as well as to aid in resistance management [4]. There are certain reports concerning the larvicidal activity of synthetic compounds [5,6,7,8,9,10,11,12].

**Figure 1:**
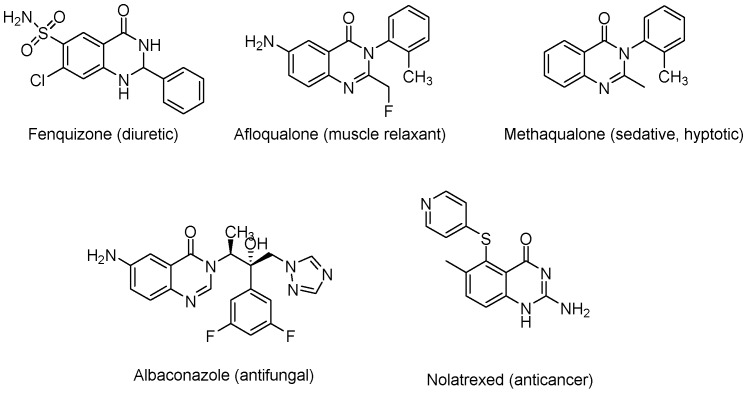
Representatives of 2,3-dihydroquinazolin-4(1*H*)-one derivatives as therapeutic agents.

The 2,3-dihydroquinazolin-4(1*H*)-ones (2,3-DHQs) are fused heterocyclic compounds which exist in natural products such as luotonins A, B, E, and F [13], tryptanthrin [14], and rutaecarpine [15]. 2,3-DHQs possess a broad range of pharmacological properties such as anti-cancer [16,17], antidepressant [18], antidiabetic [19], antifungal [20], antihypertensive [21,22], analgesic, anti-inflammatory [23,24], antibacterial [25], antioxidant [26], and antiviral [27] activities; they also act as bronchodilator [28], centrally acting muscle relaxant [29], diuretic [30], sedative, and hypnotic [31] agents (Figure 1). Quinazoline derivatives were also reported as bactericides [32], fungicides [33], and insecticides [34].

In recent years, green and sustainable chemistry drew much attention for the generation of new chemical methods with less environmental impact. In this regard, graphene oxide (GO), a carbocatalyst, attracted our attention for the preparation of quinazoline derivatives due to its non-hazardous nature and the fact that it is easy to remove from the reaction mixture.

In continuation of our efforts in developing larvicidal agents [35,36,37,38,39,40] and pharmacologically active heterocyclic compounds via green and microwave methods [41,42,43], we report herein the synthesis of 2,3-dihydroquinazolin-4(1*H*)-one derivatives, a biological evaluation of their larvicidal activity against *Anopheles arabiensis,* and an in silico evaluation of their absorption, distribution, metabolism, excretion, and toxicity (ADMET) properties as well as molecular target investigation.

## 2. Results and Discussion

### 2.1. Chemistry

**Scheme 1:**
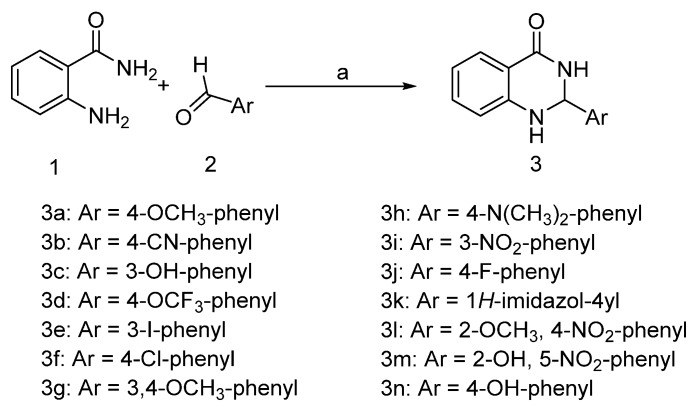
Synthetic scheme for the construction of 2-(substituted phenyl)-2,3-dihydroquinazolin-4(1*H*)-ones (**3a**–**n**). Reagents and conditions: (**a**) graphene oxide (2.5%), water, stirred at room temperature; yield 82–95%.

An efficient protocol was reported for the synthesis of 2-(substituted phenyl)-2,3-dihydroquinazolin-4(1*H*)-ones using graphene oxide as carbocatalyst [44]. The title compounds (**3a**–**n**) were prepared in excellent yields as racemic mixtures (Table 1) via direct one-pot condensation of anthranilamide (**1**) with substituted aryl aldehydes (**2**) in the presence of GO, as depicted in Scheme 1.

The yields obtained were excellent in the range of 82–95%. The purity of the title compounds was ascertained by high-performance liquid chromatography (HPLC), and it was found to be over 99%. Fourier-transform infrared spectroscopy (FT-IR) of 2,3-dihydroquinazolin-4(1*H*) one analogues (**3a**–**n**) revealed a secondary amine and carbonyl carbon of six-membered heterocyclic rings in the ranges of 3296–3382 and 1614–1660 cm^−1^, respectively. The title compounds **3c**, **3m**, and **3n** exhibited broad peaks for a phenolic hydroxyl group at 3357, 3396, and 3355 cm^−1^, respectively. Stretching of the cyano group was observed at 2223 cm^−1^ for compound **3b**. Compound **3d** exhibited a peak at 1163 cm^−1^ for carbon–fluorine stretching. Proton NMR spectra of the title compounds (**3a–n**) revealed a singlet signal corresponding to methine proton (–CH–) of the six-membered heterocyclic rings in the range of *δ* = 5.57–6.08 ppm. Compounds **3a–j** and **3l–n** revealed a singlet for an amide –CONH– functional group in the range of *δ* = 8.10–8.57 ppm, whereas derivative **3k** revealed a singlet at *δ* = 8.09 ppm for a secondary amine in the imidazole ring system. Title compounds **3c** and **3n** as mono-hydroxy derivatives exhibited a singlet at *δ* = 9.53 ppm, whereas compound **3m** exhibited a singlet for a hydroxy group at *δ* = 11.66 ppm. For all the title compounds, aromatic protons were observed as a singlet, doublet, doublet of a doublet, and multiplet depending on the substitution on the phenyl ring in the range of *δ* = 8.18–5.57. Title compound **3a** exhibited a singlet signal attributed to methoxy group at 3.75 ppm, whereas compound **3g** exhibited two signals for methoxy group at 3.75 and 3.77 ppm. The dimethylamino group was observed as a singlet for compound **3h** at 2.88 ppm. Compound **3l** exhibited a singlet signal for methoxy at 3.98 ppm. ^13^C-NMR spectra of compounds **3a**–**n** exhibited a singlet signal corresponding to a carbonyl signal in the range of *δ* = 163.39–163.86 ppm. The elemental analysis results were within ±0.4% of the calculated values. ChemDraw 16.0.1.4v professional was used to calculate the *c*Log*P* values of the title compounds (**3a–n**), and it was found to be in the range of 0.1488–3.4428.

### 2.2. Crystallography

#### Crystal Structure Analysis of 2-(3,4-Dimethoxyphenyl)-2,3-dihydroquinazolin-4(1*H*)-one (**3g**)

**Figure 2:**
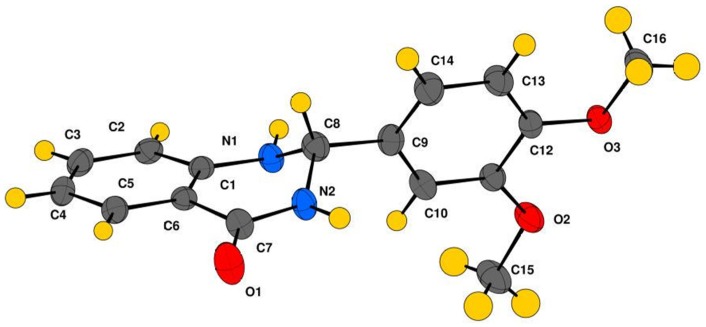
ORTEP representation (with 50% displacement ellipsoids) of the title compound (**3g**) showing the atom numbering scheme.

**Figure 3:**
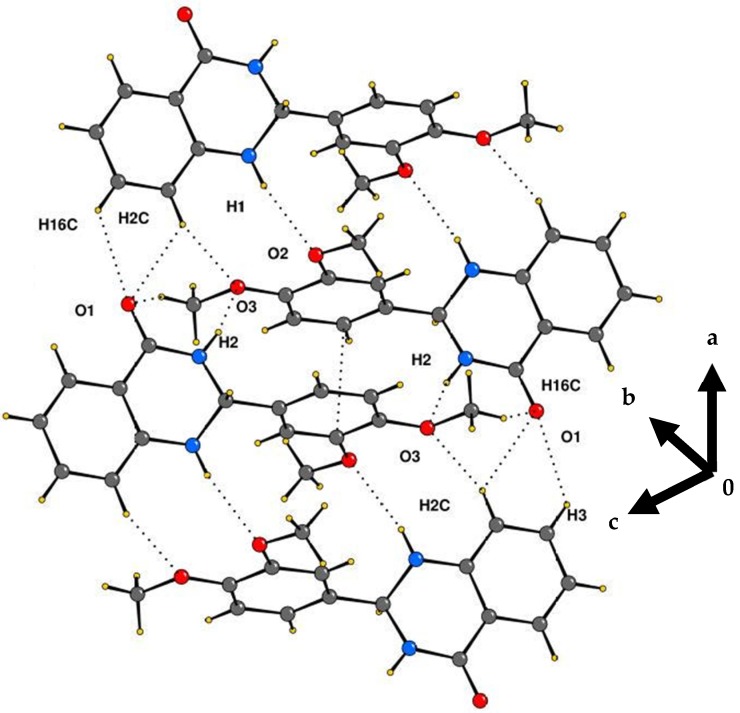
Packing arrangement of molecules in the crystal structure via formation of tetramers involving N–H···O hydrogen bonds (H-bonds), C–H···O interactions, and π–π stacking interactions.

The compound **3g** crystallized in the monoclinic centrosymmetric space group *P*2_1_/*n* with *Z* = 4 (Table 2). Figure 2 gives the Oak Ridge Thermal Ellipsoid Plot (ORTEP) view of the title compound and crystal data was deposited in Cambridge Crystallographic Data Centre (CCDC) with its number 1983734. The molecular conformation of the central ring exists in the envelope conformation, with the displacement of the atom C8 from the mean plane passing through C1–C6–C7–N2–N1 being around 0.624 (3) Å. The torsion angle N1–C8–C9–C10 is 44.3(3) indicating conformational flexibility of the molecule with respect to the C8–C9 bond. The crystal structure is connected via strong N–H···O hydrogen bonds (H-bonds) (involving H2/O3) along with C–H···O interactions (involving H16C/O1) forming a dimer (Table 3). This dimer is further supported via additional N–H···O H-bonds (involving H1/O2) and C–H···O intermolecular interactions (involving H2C/H3 with O1 and H2C with O3), forming a tetramer which is further connected with similar tetramers via van der Waals interactions (Figure 3).

### 2.3. Larvicidal Activity

The larvicidal test of the racemic title compounds (**3a–n**) was conducted at a concentration of 4 µg/mL, where the positive control Temephos demonstrated 99% to 100% mortality. The larvicidal action of the title compounds (**3a–n**) is described in Table 4. Significant larvicidal effects of the compounds were observed (F = 26.48, *p* < 0.0001), with all the compounds resulting in a higher larval mortality than exposure to the negative control. Moreover, there was no significant effect of exposure time (F = 3.73, *p* = 0.06) on larval mortality, i.e., mortality did not significantly increase from 24 h to 48 h. Compound **3d** was the most lethal, resulting in 93% mortality, which was not statistically different from exposure to the positive control Temephos (99% to 100% mortality). Compounds **3b**, **3e**, **3f,** and **3j** were not statistically different from **3d**, resulting in larval mortalities ranging from 87% to 91% mortality after 48 h. The least lethal compounds (**3c**, **3i**, **3h**, **3k,** and **3n**) resulted in mortalities ranging from 43% to 57%. On the basis of the functionalization of aryl ring and the observed larvicidal effect, it would be possible to establish the structure–activity relationships (SAR) of the title 2,3-dihydroquinazolin-4(1*H*)-one analogues (**3a–n**). The SAR indicated that the presence of halogen at the *para* and *meta* position of aryl was favorable for the bioactivity (**3e** (3-I), **3f** (4-Cl), **3j** (4-F)). The presence of the cyano group (**3b**) and methoxy group (**3a**) at the 4-position of the aryl exhibited interesting insecticide action. The replacement of the methoxy at the 4-position by a trifluoromethoxy group considerably enhanced the larvicidal activity. However, the replacement of trifluoromethoxy by a hydroxyl group at the 4-position of aryl (**3n**) was detrimental to the activity. Similarly, weak activity was observed for the compound **3c** with a hydroxyl group at the *meta* position. When 3-OH was replaced by the nitro group (**3i**), no bioactivity improvement was identified. Disubstituted aryl compounds **3g**, **3l**, and **3m** showed moderate activity against larvae. The presence of the dimethylamino group at the *para* position (**4h**) was the least favorable substituent for the larvicidal activity. Lastly, the introduction of an imidazole ring at the 2-position of quinazoline was unfavorable for the activity, showing only 46% mortality. Additional information, such as ADMET properties and binding interactions, is needed in order to explain the observed activity of the compound.

### 2.4. In Silico Absorption, Distribution, Metabolism, Excretion, and Toxicity (ADMET) Analysis

The ADMET properties of the derivatives (**3a–n**) were predicted using Accelrys Discovery Studio 4.0 client, and they are reported in Table 5. This study revealed that many compounds were predicted to possess good to excellent drug-likeness properties with the exception of compounds **3d** (4-OCF_3_), **3e** (3-I), and **3f** (4-Cl). Blood–brain barrier penetration prediction indicated that only **3d**, **3e**, and **3f** might have the ability to readily cross the brain cell membrane; hence, they may interact with the central nervous system (CNS). The remaining compounds also showed favorable cell brain permeability with the exception of derivatives **3i** (2-OCH_3_, 4-NO_2_), **3k** (imidazole), and **3m** (2-OH, 5-NO_2_). Moreover, these compounds demonstrated elevated polar surface area (PSA) 2D, indicating poor cell membrane penetration ability. All compounds were predicted to be absorbed through the intestine to reach the bloodstream circulation and be transported through the plasma-binding carrier protein to reach the desired molecular target with the exception of compound **3k** (imidazole). As for the drug metabolism aspect, no inhibition against Cytochrome P450 (CYP450) was predicted for all the compounds, indicating favorable metabolic stability against CYP450 enzymes. However, all compounds might demonstrate some toxicity toward liver cells, as indicated from the hepatotoxicity prediction. In general, most of the compounds were observed to have a favorable ADMET profile. As for the insecticide Temephos ADMET profile, it showed that Temephos was predicted to be a highly penetrant molecule towards the cell brain membrane, explaining why Temephos provides its action to the CNS as a potent acetylcholesterase inhibitor. On the basis of ADMET analysis, this study revealed a clear correlation between the larvicidal activity and the blood–brain permeability of the compounds. Indeed, the most potent derivatives **3d** (4-OCF_3_), **3e** (3-I), and **3f** (4-Cl) were predicted to be highly permeable towards the brain cell membrane, and the permeability of less active compounds was predicted to be low and medium. This study indicates that the quinazolinones are more likely to act in the CNS of larvae.

### 2.5. Molecular Modeling

On the basis of the preliminary structure–activity relationship of the 2,3-dihydroquinazolin-4(1H)-one derivatives, we explored a variety of enzyme molecular targets in an attempt to identify the mode of action via which the compounds exert their larvicidal effect. It is well known that AChE represents the main molecular target of insecticides [51,52,53,54,55] and can be associated with the larvicidal action of our compounds. However, some insecticide enzyme targets other than AChE were reported. For instance, larvicidal inhibitors have the ability to bind to sterol carrier protein-2 [56], juvenile hormone-binding protein [57], D7r4 a salivary biogenic amine-binding protein [58], calcium-dependent protein kinase-1 [59], or purine nucleoside phosphorylase targets [60]. The binding investigation of the dihydroquinazolines against the above-mentioned molecular targets allowed us to identify AChE as the most plausible molecular target which corroborated with the predicted brain cell permeability [51,52,53,54,55,56,57,58,59,60]. No crystal structures of *Anopheles arabienesis* AChE from mosquito are deposited in the Protein Data Bank (PDB) so far. A crystal structure of *Anopheles gambiae* AChE of the malaria mosquito was recently reported with a low structure resolution of 3.4 Å that may not be exploitable for docking study [61]. Another crystal structure of *Anopheles gambiae* AChE of the malaria insecticide-resistant mosquito harboring a G119S mutation was deposited with a better resolution (2.26 Å) [54]. This mutation does not appear to perturb the other amino acids in the catalytic site. Knutsson et al. reported the synthesis and the larvicidal activity of some phenoxyacetamide derivatives [55]. An enzymatic assay of these derivatives was conducted against four AChE1 enzymes (*Anopheles gambiae* mosquito, G119S mutation *Anopheles gambiae* mosquito, mouse, and human), and it demonstrated good selectivity in favor of both mosquito AChE1 enzymes as compared to that of human AChE. Moreover, the tested derivatives showed a similar inhibition profile between the mosquito AChE1 and insecticide-resistant mutant mosquito AChE1, as well as between the mouse and human AChE. In light of the enzymatic assay, it appeared that the structure of insecticide resistant mutant mosquito AChE1 showed high structural similarity to the non-mutant one. Hence, the insecticide-resistant mutant receptor would appear to be a suitable receptor that can be used as a template for providing a structural understanding of the title compounds’ potency.

The computational study was then conducted using the co-crystal enzyme G199S mutant AChE1 mosquito (PDB code: 6ARY), with Table 6 presenting the binding energy and the residue interactions. It should be noted that the dihydroquinazoline derivatives **3a–n,** possessing a chiral center in the 2-position of the quinazoline ring, exist as *R* and *S* stereoisomer. Therefore, the docking interaction against the AChE target was conducted for each stereoisomer. The molecular modeling study revealed that the binding energies of some dihydroquinazolines were greater than the binding energy of the native ligand found in the co-crystal AChE 1. As for Temephos, we were unable to dock it in the AChE active site of the insecticide-resistant mosquito, which corroborates its resistance to insecticide. It was also predicted that, for the majority of derivatives, the *R* form was more tightly bound in the receptor than the *S* form, with the exception of compounds **3a**, **3i**, and **3n**. This may indicate that the *R* stereoisomer would be more potent than its enantiomer. The docking analysis revealed that the hydrophilic and the hydrophobic interactions contributed more or less equally to the observed larvicidal effect of the derivatives with the exception of quinazoline **3f** *R* (4-Cl). The potency of this compound might be explained by its involvement in π–π interactions with residues Tyr 282 and Tyr 489, as well as the chlorine interaction involvement with the amino acid Trp 245. Another halogen interaction was also observed for quinazoline **3e** *R* (3-I) with residue Asn 246 and for **3d** *R* (4-OCF_3_) with residues Gly 278 and Glu 359. The quinazoline ring appeared to be an important pharmacophore due to its capability of forming hydrogen bond donors from the NH and acceptors from the carbonyl, as well as a hydrophobic interaction through π–π stacking with the fused aryl ring of quinazoline. The most potent compound **3d** (4-OCF_3_) displayed very good binding energy of −110.82 kJ/mol for the *R* enantiomer, displaying three hydrogen bonding interactions with residues Gly 278 (CF_3_), Tyr 291 (CF_3_), and Tyr 489 (NH quinazoline), as well as stacking interactions with residues Trp 245 and Tyr 282. In general, the amino-acid residues Trp 245, Tyr 282, and Tyr 489 were implicated in π–π interactions with the quinazoline ring and the aromatic ring at the 2-position of the derivatives. The halogenated compounds **3e** (3-I), **3f** (4-Cl), and **3j** (4-F), exhibiting high larvicidal action, showed π–π interactions with residues Trp 245, Tyr 282, and Tyr 489 of the AChE active site. They also demonstrated hydrogen bonding involvement with residues Tyr 489 (NH) for **3e** (3-I), **3f** (4-Cl), and **3j** (4-F), and Ser 283 (NHCO) for **3f** (4-Cl) and **3j** (4-F). The importance of the hydrogen bonding contribution to the bioactivity of the compounds can also be observed. For instance, the couple **3a** (4-OCH_3_)/**3d** (4-OCF_3_) as an *R* stereoisomer adopted a similar binding mode in which the pendant aryl ring was located facing the edge of residue Trp 245 in which OCF_3_ and OCH_3_ groups were oriented toward the backbone of Gly 278 participating in H-bonding with only quinazoline **3d** (4-OCF_3_). Other residues were also involved in H-bonding with the ligands, such as Gly 279, Gly 359, Asp 233, Ser 280, Tyr 281, and Tyr 291.

It can be noted that compounds **3i** S (3-NO_2_) and **3m** R (2-OH, 5-NO_2_) exhibited a very strong binding affinity to the receptor. However, **3i** and **3m** demonstrated modest larvicidal activities of 0.52 and 0.59, respectively. The lack of correlation between the bioactivity and the docking results may be associated with the lipophilicity of the compounds. Indeed, from the ADMET properties, both compounds were predicted to weakly cross the brain cell membrane, preventing their full action toward the CNS of the larvae. It is interesting to note that, for all other compounds, the docking results for the R enantiomer were in agreement with the larvicidal effect of the compounds with the exception of **3n** (4-OH), showing a high predicted binding energy of −121.58 for the S stereoisomer. The less potent compounds, **3h** (4-N(CH_3_)_2_) and **3k** (imidazole), demonstrated weaker binding energies as compared to their congeners.

**Figure 4:**
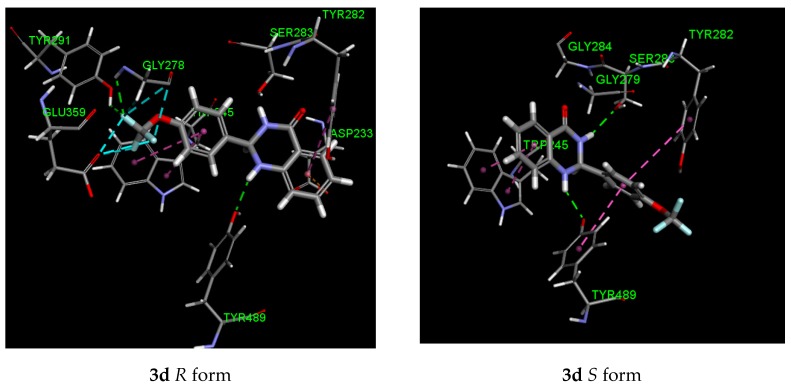
Predicted binding mode of both enantiomers of **3d** in the G199S mutant AChE receptor (PDB: 6ARY). The molecular interactions are represented as green dotted lines for H-bonds, magenta dotted lines for π–π stacking interactions, and cyan dotted lines for fluorine contacts.

The binding interactions of the most active compound **3d** with the G199S mutant AChE receptor are illustrated in Figure 4, showing a different binding mode for each enantiomer. The binding mode of **3d** *R* demonstrated three hydrogen bonding interactions with amino-acid residues Tyr 291 and Gly 278 with the fluorine atom, and residue Tyr 489 with the NH of the quinazoline ring. Additionally, several fluorine interactions were also observed with residues Gly 278 and Glu 359. Furthermore, π–π hydrophobic interactions were predicted between the aryl of the quinazoline at the 2-position and both rings of tryptamine residue Trp 245 facing its edge, and the quinazoline moiety was involved in a T-shaped π–π interaction with Tyr 282 and a π–ion interaction with amino acid Asp 233. In contrast, the binding mode of enantiomer **3d** *S* was predicted to be inverted, in which the quinazoline ring interacted with residue Trp 245 facing its edge via π–π interaction, while the aryl ring showed two π–π interactions with residues Tyr 282 and Tyr 489. Two hydrogen bonding interactions were also predicted with the two NH groups of the quinazoline ring and residues Gly 279 and Tyr 489.

Our molecular modeling input revealed that the AChE enzyme target might be the plausible enzyme target and the *R* enantiomer would be the principal active compound for larvicidal activity contribution. This study may also indicate that our compounds might be potentially active against insecticide-resistant mosquitos due to the good fitting observed with the active site of the G199S mutant AChE receptor. However, future work should address the identification of the enzyme target, the enantiomer responsible for the insecticidal effect, and the potency against the insecticide-resistant mosquito.

## 3. Materials and Methods

### 3.1. General

The chemicals reported here were obtained from Sigma-Aldrich Co. (St. Louis, MO, USA), while the solvents were obtained from Millipore Sigma (Burlington, MA, USA). Thin-layer chromatography (TLC) using silica gel (Sigma-Aldrich Co.) on aluminum foil was employed to observe the chemical reactions; *n*-hexane and ethyl acetate (4:6) were used as solvents. The reactions were visualized under an ultraviolet (UV)-light/iodine chamber. B-545 was used to measure the melting points (Büchi, Labortechnik, Flawil, Switzerland). Fourier-transform infrared (FT-IR) spectra were recorded on a Shimadzu FT-IR spectrophotometer. Furthermore, ^1^H- and ^13^C-NMR spectra were recorded on Bruker AVANCE III 400 MHz instruments using dimethyl sulfoxide (DMSO)-*d*6 as a solvent. Chemical shifts (*δ*) were recorded in parts per million (ppm) downfield from tetramethylsilane, while the coupling constants (*J*) were recorded in Hz. The splitting pattern was documented as follows: s, singlet; d, doublet; q, quartet; m, multiplet. Liquid chromatography–mass spectrometry (LC–MS; Agilent 1100 series) was used to measure the mass spectra in conjunction with the mass selective detector (MSD), as well as 0.1% aqueous trifluoroacetic acid in an acetonitrile system on the C18-BDS column. Then, elemental analysis was carried out using the analyzer FLASH EA 1112 CHN (Thermo Finnigan LLC, New York, NY, USA). A single-crystal X-ray diffraction study was performed using a Bruker KAPPA APEX II DUO diffractometer equipped with a charge coupled device (CCD) detector; monochromated Mo K*α* radiation (λ = 0.71073 Å) was used. Data collection was carried out at 173(2) K using an Oxford Cryostream cooling system featuring the Bruker Apex II software.

### 3.2. General Procedure for the Synthesis of 2-(Substituted phenyl)-2,3-dihydroquinazolin-4(1H)-ones (3**a**–**n**)

A mixture of aryl aldehyde (1.5 mmoL) and 2-aminobenzamide (1 mmoL) in 10 mL of water was stirred in a round-bottom flask with graphene oxide (2.5%) at room temperature, as depicted in Scheme 1. The progress of the reaction was monitored with TLC. After completion of the reaction, the insoluble precipitate obtained was separated and filtrated, and the crude product was washed with 50% cold ethanol. The crude product was recrystallized from 75% ethyl alcohol to obtain 65% of the title compound 2-(4-fluorophenyl)-2,3-dihydroquinazolin-4(1*H*)-one at 98% purity [44]. Physicochemical characteristics of the title compounds are tabulated in Table 1. FT-IR, ^1^H and ^13^C-NMR of the title compounds (**3a–n**) are available as electronic supplementary information.

#### 3.2.1. 2-(4-Methoxyphenyl)-2, 3-dihydroquinazolin-4(1*H*)-one (**3a**)

FT-IR (KBr, cm^−1^) 3298, 3182, 2862, 1658, 1610, 1508, 1483, 1298, 1174, 802, 611. ^1^H-NMR (400 MHz, DMSO-*d*_6_) *δ* = 8.20 (s, 1H), 7.64–7.62 (dd, *J* = 8 Hz, 1.6 Hz,1H), 7.45–7.42 (m, 2H), 7.27–7.23 (m, 1H), 7.02 (s, 1H), 6.97–6.94 (m, 2H), 6.77–6.70 (m,1H), 6.68–6.66 (m, 1H), 5.72 (s, 1H), 3.75 (s, 3H); ^13^C-NMR (100 MHz, DMSO-d_6_) δ = 163.70, 159.41, 147.99, 133.42, 133.22, 128.19, 127.32, 117.07, 114.97, 114.39, 113.60, 66.29, 55.13. Analytically calculated for C_15_H_14_N_2_O_2_: C, 70.85; H, 5.55; N, 11.02; found: C, 70.75; H, 5.50; N, 10.98.

#### 3.2.2. 4-(4-Oxo-1,2,3,4-tetrahydroquinazolin-2-yl)benzonitrile (**3b**)

FT-IR (KBr, cm^−1^) 3440, 2223, 1664, 1610, 1502, 1373, 1153, 858, 771, 619. ^1^H-NMR (400 MHz, DMSO-*d*_6_) *δ* = 8.52 (s, 1H), 7.88–7.86 (d, *J* = 8 Hz, 2H), 7.71–7.69 (d, *J* = 8.4 Hz, 2H), 7.66–7.64 (d, *J* = 7.6 Hz, 1H), 7.30–7.25 (m, 2H), 6.81–6.79 (d, *J* = 8.4 Hz, 1H), 6.72–6.70 (d, *J* = 8.0 Hz,1H), 5.89 (s, 1H); ^13^C-NMR (100 MHz, DMSO-*d*_6_) *δ* = 163.40, 147.31, 147.24, 133.55, 132.36, 132.36, 127.68, 127.68, 127.41, 118.62, 117.45, 114.87, 114.52, 111.10, 65.59. Analytically calculated for C_15_H_11_N_3_O: C, 72.28; H, 4.45; N, 16.86; found: C, 72.15; H, 4.40; N, 16.78.

#### 3.2.3. 2-(3-Hydroxyphenyl)-2,3-dihydroquinazolin-4(1*H*)-one (3**c**)

FT-IR (KBr, cm^−1^) 3357, 3296, 1631, 1606, 1506, 1483, 1305, 1155, 1035, 865, 752. ^1^H-NMR (400 MHz, DMSO-*d*_6_) *δ* = 9.53 (s, 1H), 8.28 (s, 1H), 7.66–7.64 (d, *J* = 7.6 Hz, 1H), 7. 27–7.17 (m, 2H), 7.10 (s, 1H), 6.95–6.92 (m, 2H), 6.78–6.76 (br d, *J* = 8.4 Hz, 2H), 6.71–6.67 (m, 1H), 5.69 (s, 1H); ^13^C-NMR (100 MHz, DMSO-*d*_6_) *δ* = 163.57, 157.34, 147.80, 143.17, 133.30, 129.32, 127.34, 117.44, 117.01, 115.36, 114.81, 114.34, 113.63, 66.47. Analytically calculated for C_14_H_12_N_2_O_2_: C, 69.99; H, 5.03; N, 11.66; found: C, 69.85; H, 4.96; N, 11.58.

#### 3.2.4. 2-(4-(Trifluoromethoxy)phenyl)-2,3-dihydroquinazolin-4(1*H*)-one (3**d**)

FT-IR (KBr, cm^−1^) 3296, 3184, 3074, 1652, 1614, 1510, 1440, 1278, 1163, 1018, 813, 792, 675. ^1^H-NMR (400 MHz, DMSO-*d*_6_) *δ* = 8.36 (s, 1H), 7.66–7.63 (m, 3H), 7.42–7.40 (d, *J* = 8 Hz, 2H), 7.29–7.24 (m, 1H), 7.17 (s, 1H), 6.78–6.76 (br d, *J* = 8.4 Hz, 1H), 6.72–6.68 (m,1H), 5.84 (s, 1H); ^13^C-NMR (100 MHz, DMSO-*d*_6_) *δ* = 163.49, 148.30, 148.28, 147.93, 133.38, 128.91, 127.36, 121.30, 118.76, 117.29, 114.88, 114.42, 65.82. Analytically calculated for C_15_H_11_F_3_N_2_O_2_: C, 58.45; H, 3.60; N, 9.09; found: C, 58.05; H, 3.46; N, 8.88.

#### 3.2.5. 2-(3-Iodophenyl)-2,3-dihydroquinazolin-4(1*H*)-one (3**e**)

FT-IR (KBr, cm^−1^) 3282, 3078, 2925, 1649, 1614, 1514, 1446, 1296, 1159, 811, 790, 754, 696. ^1^H-NMR (400 MHz, DMSO-*d*_6_) *δ* = 8.39 (s, 1H), 7.88 (s, 1H), 7.72–7.70 (d, *J* = 8.0 Hz, 1H), 7.65–7.63 (dd, *J* = 8.0, 1.6 Hz, 1H), 7.54–7.51 (d, *J* = 7.2 Hz, 1H), 7.29–7.17 (m, 3H), 6.79–6.77(d, *J* = 6.8 Hz, 1H), 6.72–6.68 (m, 1H), 5.77 (s, 1H); ^13^C-NMR (100 MHz, DMSO-*d*_6_) *δ* = 163.45, 147.51, 144.35, 137.02, 135.50, 133.45, 130.54, 127.38, 126.17, 117.31, 114.84, 114.45, 94.76, 65.51. Analytically calculated for C_14_H_11_IN_2_O: C, 48.02; H, 3.17; N, 8.00; found: C, 47.90; H, 3.06; N, 7.88.

#### 3.2.6. 2-(4-Chlorophenyl)-2,3-dihydroquinazolin-4(1*H*)-one (**3f**)

FT-IR (KBr, cm^−1^) 3450, 3307, 3190, 3064, 2933, 1658, 1608, 1485, 1386, 1153, 838, 795, 752, 667. ^1^H-NMR (400 MHz, DMSO-*d*_6_) *δ* = 8.37 (s, 1H), 7.66–7.64 (d, *J* = 7.6 Hz, 1H), 7.55–7.53 (m, 2H), 7.48–7.45 (m, 2H), 7.29–7.24 (m, 1H), 7.16 (s, 1H), 6.79–6.77 (d, *J* = 8.0 Hz, 1H), 6.72–6.68 (m,1H), 5.80 (s, 1H); ^13^C-NMR (100 MHz, DMSO-*d*_6_) *δ* = 163.53, 147.64, 140.61, 133.39, 133.00, 128.74, 128.29, 127.37, 117.29, 114.92, 114.46, 65.59. Analytically calculated for C_14_H_11_ClN_2_O: C, 65.00; H, 4.29; N, 10.83; found: C, 64.90; H, 4.16; N, 10.68.

#### 3.2.7. 2-(3,4-Dimethoxyphenyl)-2,3-dihydroquinazolin-4(1*H*)-one (**3g**)

FT-IR (KBr, cm^−1^) 3450, 3330, 3299, 2933, 1660, 1610, 1510, 1488, 1369 1262, 1232, 1140, 1091, 810. ^1^H-NMR (400 MHz, DMSO-*d*_6_) *δ* = 8.21 (s, 1H), 7.65–7.63 (d, *J* = 7.0 Hz,1H), 7.28–7.26 (m, 1H), 7.16–7.15 (d, *J* = 1.6 Hz, 1H), 7.03–7.01 (m, 2H), 6.96–6.94 (m, 1H), 6.79–6.77 (d, *J* = 8.0 Hz,1H), 6.71–6.68 (m, 1H), 5.72 (s, 1H), 3.77 (s, 3H), 3.75 (s, 3H); ^13^C-NMR (100 MHz, DMSO-*d*_6_) *δ* = 163.76, 148.99, 148.58, 148.05, 133.55, 133.22, 127.33, 119.20, 117.14, 115.03, 114.43, 111.24, 110.60, 66.55, 55.54, 55.43. Analytically calculated for C_16_H_16_N_2_O_3_: C, 67.59; H, 5.67; N, 9.85; found: C, 66.90; H, 5.36; N, 09.68.

#### 3.2.8. 2-(4-(Dimethylamino)phenyl)-2,3-dihydroquinazolin-4(1*H*)-one (**3h**)

FT-IR (KBr, cm^−1^) 3450, 3296, 3193, 3060, 2932, 1658, 1614, 1485, 1357, 1296, 1161, 1064, 817, 754. ^1^H-NMR (400 MHz, DMSO-*d*_6_) *δ* = 8.10 (s, 1H), 7.66–7.64 (dd, *J* = 8.4, 1.2 Hz, 1H), 7.34–7.32 (d, *J* = 8.8 Hz, 2H), 7.27–7.22 (m, 1H), 6.94 (s, 1H), 6.78–6.67 (m, 4H), 5.67 (s, 1H), 2.88 (s, 6H); ^13^C-NMR (100 MHz, DMSO-*d*_6_) *δ* = 163.86, 150.67, 148.21, 133.12, 128.59, 127.70, 127.34, 116.94, 115.03, 114.38, 111.90, 111.90, 66.68, 40.12. Analytically calculated for C_16_H_17_N_3_O: C, 71.89; H, 6.41; N, 15.72; found: C, 71.40; H, 6.36; N, 15.48.

#### 3.2.9. 2-(3-Nitrophenyl)-2,3-dihydroquinazolin-4(1*H*)-one (**3i**)

FT-IR (KBr, cm^−1^) 3296, 3193, 3074, 2923, 1652, 1610, 1530, 1485, 1352, 1257, 1153, 1033, 904, 752. ^1^H-NMR (400 MHz, DMSO-*d*_6_) *δ* = 8.57 (s, 1H), 8.39 (s, 1H), 8.22–8.19 (m, 1H), 7.97–7.95 (d, *J* = 8.0 Hz, 1H), 7.71–7.63 (m, 2H), 7.36 (s, 1H), 7.30–7.26 (m, 1H), 6.82–6.80 (d, *J* = 8.0 Hz, 1H), 6.72–6.69 (m, 1H), 5.98 (s, 1H); ^13^C-NMR (100 MHz, DMSO-*d*_6_) *δ* = 163.39, 147.68, 147.27, 144.24, 133.57, 133.32, 129.97, 127.41, 123.23, 121.55, 117.53, 114.91, 114.58, 65.19. Analytically calculated for C_14_H_11_N_3_O_3_: C, 62.45; H, 4.12; N, 15.61; found: C, 62.06; H, 4.06; N, 15.37.

#### 3.2.10. 2-(4-Fluorophenyl)-2,3-dihydroquinazolin-4(1*H*)-one (**3j**)

FT-IR (KBr, cm^−1^) 3400, 3299, 3184, 3130, 2931, 1658, 1610, 1508, 1485, 1389, 1234, 1159, 836, 750, 675. ^1^H-NMR (400 MHz, DMSO-*d*_6_) *δ* = 8.31 (s, 1H), 7.65–7.63 (dd, *J* = 8.8, 1.2 Hz, 1H), 7.58–7.55 (m, 2H), 7.26–7.21 (m, 3H), 7.12 (s, 1H), 6.78–6.76 (d, *J* = 8.0 Hz, 1H), 6.72–6.68 (m, 1H), 5.80 (s, 1H); ^13^C-NMR (100 MHz, DMSO-*d*_6_) *δ* = 163.57, 147.78, 137.76, 137.73, 133.34, 129.06, 128.97, 127.35, 117.24, 115.17, 114.96, 114.92, 114.43, 65.93. Analytically calculated for C_14_H_11_FN_2_O: C, 69.41; H, 4.58; N, 11.56; found: C, 69.06; H, 4.46; N, 11.37.

#### 3.2.11. 2-(1*H*-Imidazol-4-yl)-2,3-dihydroquinazolin-4(1*H*)-one (**3k**)

FT-IR (KBr, cm^−1^) 3247, 3130, 1614, 1579, 1515, 1440; ^1^H-NMR (400 MHz, DMSO-*d*_6_) *δ* = 8.09 (s, 1H), 7.49–7.48 (dd, *J* = 6.4, 1.2 Hz, 2H), 7.09–7.05 (m, 1H), 6.89, (s, 1H), 6.85, (s, 1H), 6.64–6.62 (d, *J* = 8.0, 1H), 6.53–6.50 (m, 1H), 5.57 (s, 1H); ^13^C-NMR (100 MHz, DMSO-*d*_6_) *δ* = 163.84, 147.96, 135.37, 133.08, 127.30, 117.07, 115.19, 114.59, 61.67. Analytically calculated for C_11_H_10_N_4_O: C, 61.67; H, 4.71; N, 26.15; found: C, 61.06; H, 4.56; N, 25.37.

#### 3.2.12. 2-(2-Methoxy-4-nitrophenyl)-2,3-dihydroquinazolin-4(1*H*)-one (**3l**)

FT-IR (KBr, cm^−1^) 3371, 3022, 2929, 1649, 1616, 1519, 1485, 1348, 1253, 1163, 1089, 887, 765, 740. ^1^H-NMR (400 MHz, DMSO-*d*_6_) *δ* = 8.23 (s, 1H), 7.87–7.85 (dd, *J* = 8.4, 2.4 Hz, 1H), 7.81–7.80 (m, 1H), 7.66–7.60 (m, 2H), 7.27–7.23 (m, 1H), 6.99 (s, 1H), 6.80–6.78 (d, *J* = 8.0 Hz, 1H), 6.71–6.67 (m, 1H), 6.08 (s, 1H) 3.98 (s, 3H); ^13^C-NMR (100 MHz, DMSO-*d*_6_) *δ* = 163.53, 156.76, 148.37, 147.32, 136.36, 133.46, 127.56, 127.32, 117.35, 115.38, 114.54, 114.49, 105.84, 60.71, 56.33. Analytically calculated for C_15_H_13_N_3_O_4_: C, 60.20; H, 4.38; N, 14.04; found: C, 60.06; H, 4.16; N, 13.87.

#### 3.2.13. 2-(2-Hydroxy-5-nitrophenyl)-2,3-dihydroquinazolin-4(1*H*)-one (**3m**)

FT-IR (KBr, cm^−1^) 3396, 3195, 2925, 1645, 1623, 1515, 1392, 1336, 1284, 1164, 1089, 833, 757, 628. ^1^H-NMR (400 MHz, DMSO-*d*_6_) *δ* = 11.66 (s, 1H), 8.24–8.23 (d, *J* = 7.2 Hz, 1H), 8.18–8.12 (m, 2H), 7.67–7.65 (d, *J* = 7.6 Hz, 1H), 7.28–7.24 (m, 1H), 7.07–7.05 (d, *J* = 9.2 Hz, 1H), 6.95 (s, 1H), 6.82–6.80 (d, *J* = 8.0 Hz,1H), 6.73–6.69 (m, 1H) 6.05–6.04 (m, 1H); ^13^C-NMR (100 MHz, DMSO-*d*_6_) *δ* = 163.69, 161.30, 147.60, 139.19, 133.44, 128.28, 127.37, 125.78, 123.31, 117.44, 115.95, 114.60, 114.60, 60.80. Analytically calculated for C_14_H_11_N_3_O_4_: C, 58.95; H, 3.89; N, 14.73; found: C, 58.77; H, 3.77; N, 14.43.

#### 3.2.14. 2-(4-Hydroxyphenyl)-2,3-dihydroquinazolin-4(1*H*)-one (3**n**)

FT-IR (KBr, cm^−1^) 3355, 3296, 1631, 1606, 1506, 1481, 1463, 1278, 1157, 1130, 865, 752. ^1^H-NMR (400 MHz, DMSO-*d*_6_) *δ* = 9.53 (s, 1H), 8.12 (s, 1H), 7.65–7.63 (d, *J* = 7.2 Hz, 1H), 7.34–7.32 (d, *J* = 8.4 Hz, 2H), 7.27–7.25 (m, 1H), 6.95–6,92 (m, 1H), 6.80–6.75 (m, 4H), 5.68 (s, 1H); ^13^C-NMR (100 MHz, DMSO-*d*_6_) *δ* = 163.78, 157.67, 148.14, 133.23, 131.56, 128.28, 127.34, 117.04, 114.92, 114.37, 66.65. Analytically calculated for C_14_H_12_N_2_O_2_: C, 69.99; H, 5.03; N, 11.66; found: C, 69.56; H, 5.87; N, 11.43.

### 3.3. Crystallographic Studies

Single-crystal X-ray diffraction data were collected on a Bruker KAPPA APEX II DUO diffractometer using graphite monochromated Mo Kα radiation (χ = 0.71073 Å). Data collection was carried out at 173(2) K. Temperature was controlled by an Oxford Cryostream cooling system (Oxford Cryostat). Cell refinement and data reduction were performed using the program SAINT [62]. The data were scaled, and absorption correction was performed using SADABS [62].

The structure was solved via direct methods using SHELXS-18 [63] and refined via the full-matrix least-squares method based on F^2^ using SHELXL-2018 [63]. The program WinGx [64] was used to prepare molecular graphic images. All non-hydrogen atoms were refined anisotropically. All hydrogen atoms, except H1 and H2, were placed in idealized positions and refined in riding models with U_iso_ assigned 1.2 or 1.5 times the U_eq_ of their parent atoms, and the C–H bond distances were constrained to 0.95 Å for aromatic hydrogen and 1.00 Å for methyl hydrogen. The amino hydrogens H1 and H2 were located in the difference electron density maps and refined independently. There was a solvent-accessible void of 411.3 Å^3^ per unit cell volume of 2587.0 Å^3^ (~15.9%). Attempts to model the solvent molecule as discrete atomic sites failed. PLATON SQUEEZE [65] was employed to calculate the contribution to the diffraction from the missing solvent molecules, and it produced a set of partial solvent-free diffraction intensities. This set of intensities was used for final refinements. SQUEEZE estimated a total count of 99 electrons per unit cell, which were contributed by the missing solvents. The structure was refined to an R factor of 0.0544. The parameters for crystal data collection and structure refinement and the list of intermolecular interactions in the crystal structure 2-(3,4-dimethoxyphenyl)-2,3-dihydroquinazolin-4(1*H*)-one (**3g**) are given in Table 2 and Table 3, respectively.

### 3.4. Larvicidal Activity

*Anopheles arabiensis* was used in the study according to the protocol described by WHO (1975) guidelines [66] in an insectary, simulating the temperature (27.5 °C), humidity (70%), and lighting (12/12) of a malaria-endemic environment. One milliliter of test compound (1 mg/mL) was added to distilled water (250 mL) to obtain a final concentration of 4 µg/mL. Thirty instar larvae were introduced into a container. A negative control was set up using the solvent (Acetone), and distilled water, as well as a positive control Temephos, which is an active emulsified organophosphate larvicidal drug in malaria control programs. Larval mortality was examined for each container separately for 24 h and 48 h, and larvae were fed specially made cat food that contained less oil/fat content. The percentage mortality was determined relative to the initial number of larvae exposed. The bioassay was performed in triplicate, and the results are tabulated in Table 4.

### 3.5. Data Analysis

Differences in larval mortality between treatments were assessed with generalized linear models using a binomial link function [67]. The dependent variable was *A. arabiensis* mortality, while fixed effects were test compound (test compounds **3a**–**n**, and both controls) and observation period (24 and 48 h). A *p*-value <0.05 was considered statistically significant. Throughout the text, the results are presented as the adjusted mean ±the standard error.

### 3.6. In silico ADMET Prediction

ADMET properties of the derivatives (**3a–n**) were predicted using the software Accelry’s Discovery Studio 4.0 Client [68]. ADMET-related descriptors such as drug-likeness, permeability, intestine absorption, plasma binding, liver toxicity, and CYP450 inhibition were investigated. The in silico ADMET properties of quinazolines (**3a–n**) and Temephos are tabulated in Table 5.

### 3.7. Molecular Modeling

Docking simulation was conducted with the software Accelry’s Discovery Studio 4.0 client employing the CHARMm force field algorithm following our earlier reported procedure [69,70]. In this study, the X-ray co-crystal acetylcholinesterase receptor (PDB: 6ARY) was retrieved from the Protein Data Bank (PDB) [71] and subjected to the following steps: removal of all ligands and water; adding hydrogen and missing amino acids. The binding domain was defined by inserting the original ligand into the prepared protein. To validate the active site, the original ligand was re-docked to ensure proper binding interactions with respect to those reported for PDB 6FSD. CDocker protocol was used to predict the binding mode of compounds. The active amino-acid residues of the receptor were kept rigid during the docking run. The conformation of the best 10 binding poses was ranked according to the CDocker energy score. The most proper binding mode was selected according to the highest-scoring functions PLP1, PLP2, Jain, and PMF [70]. The binding energy of the selected ligand binding pose was determined by applying the in situ ligand minimization energy step in the CDocker protocol.

## 4. Conclusions

The 2,3-dihydroquinazolin-4(1*H*)-one represents a privileged scaffold exhibiting various pharmacological activities. The functionalization of the quinazoline at the 2-position with aryl led to potent medicinal agents with anti-tubulin, antifungal, anti-tuberculosis, and antiviral activities, among others. Thus, the 2-aryl-2,3-dihydroquinazolin-4-one scaffold makes compounds attractive in terms of exploring novel activity. The novel series of 2-aryl-2,3-dihydroquinazolin-4-ones were identified as potent insecticides against larvae *Anopheles arabiensis.* These compounds exhibited potent larvicidal effects for the control of malaria vectors due to their toxicity towards larvae, when exposed to a concentration of 4 µg/mL. The most promising compound was found to be **3d**, substituted in the *para* position with an OCF_3_ group, which displayed a similar toxicity to that of Temephos, an active emulsified organophosphate larvicidal used by malaria control programs. The next two potent compounds were **3j** and **3f,** which resulted in over 90% larval mortalities after 24- to 48-h exposure. The ADMET profile for the majority of the compounds was acceptable. The docking input revealed that AChE would be the appropriate enzyme molecular target upon demonstrating a favorable binding interaction, and the title compounds may find application for treating insecticide-resistant mosquitos. In general, the *R* enantiomer was predicted to strongly bind to the AChE receptor. Based on ADMET and docking analysis, the title compounds are investigated as potential AChE inhibitors, and the brain cell permeability was an important parameter for the activity. However, elucidation of the molecular target and the enantiomer responsible for larvicidal activity remains to be addressed in order to design more potent and safer 2,3-dihydroquinazolin-4(1*H*)-ones. Therefore, the dihydroquinazolinone scaffold having a *para* trifluoromethoxy functionality on the phenyl ring was identified as the lead compound, which can serve as the starting point for the development of novel larvicidal agents.

## Figures and Tables

**Table 1:** Physicochemical characteristics of 2-(substituted phenyl)-2,3-dihydroquinazolin-4(1*H*)-ones (**3a–n**).

Compound Code	Ar	Molecular Formula (Molecular Mass)	Yield (%)	m.p (°C) Reported	mp (°C) Found	cLog*P*	References	CAS Number
**3a**	4-OCH_3_-Ph	C_15_H_14_N_2_O_2_ (254)	95	182–183	181–182	2.2388	[45]	61195-16-2
**3b**	4-CN-Ph	C_15_H_11_N_3_O (249)	92	178–180	178–180	1.7528	[44]	219619-59-7
**3c**	3-OH-Ph	C_14_H_12_N_2_O_2_ (240)	91	184–186	185–187	1.6528	[46]	107922-06-5
**3d**	4-OCF_3_-Ph	C_15_H_11_F_3_N_2_O_2_ (308)	94	194–196	195–196	3.3478	[47]	685505-75-3
**3e**	3-I-Ph	C_14_H_11_IN_2_O (349)	92	--	153–155	3.4428	Novel Compound	
**3f**	4-Cl-Ph	C_14_H_11_ClN_2_O (258)	87	197–198	198–199	3.0328	[44]	13165-11-2
**3g**	3,4-diOCH_3_-Ph	C_16_H_16_N_2_O_3_ (284)	88	214–216	213–215	1.9778	[46]	126492-17-9
**3h**	4-*N*,*N* dimethyl-Ph	C_16_H_17_N_3_O (267)	93	209–211	210–211	2.4848	[48]	66181-66-6
**3i**	3-NO_2_-Ph	C_14_H_11_N_3_O_3_ (269)	91	192–194	192–1193	2.0628	[44]	26029-30-1
**3j**	4-F-Ph	C_14_H_11_FN_2_O (242)	91	196–198	197–198	2.4628	[48]	359605-44-0
**3k**	Imidazole	C_11_H_10_N_4_O (214)	87	--	156–158	0.1488	Novel Compound	
**3l**	2-OCH_3_-4-NO_2_-Ph	C_15_H_13_N_3_O_4_ (299)	93	--	149–151	2.2818	Novel Compound	
**3m**	2-OH, 5-NO_2_-Ph	C_14_H_11_N_3_O_4_ (285)	89	244–246	245–246	1.9818	[49]	1794721-69-9
**3n**	4-OH-Ph	C_14_H_12_N_2_O_2_ (240)	95	209–211	210–211	1.6528	[50]	107920-18-3

Ar: Aromatic; m.p: Melting point; CAS: Chemical Abstracts Service.

**Table 2:** Single-crystal X-ray data of title compound 2-(3,4-dimethoxyphenyl)-2,3-dihydroquinazolin-4(1*H*)-one (**3g**).

CCDC Number	1983734
Molecular Formula	C_16_H_16_N_2_O_3_
Molecular weight	284.31
Temperature	173(2)
Crystal Size (mm)	0.2, 0.110, 0.040
Absorption coefficient (mm^−1^)	0.096
T_min_, T_max_	0.981, 0.996
Crystal system	Monoclinic
Lattice parameters: a (Å), b (Å), c (Å)	7.8881(6), 20.2194 (13), 8.9638 (6)
*α, β, γ* (°)	90, 104.887(3), 90
Space Group, Density, Z, Z′	*P*2_1_/*n*, 1.367, 4, 1
h_min, max_; k_min, max_; l_min, max_;	−8, 10; −26, 26; −11, 11
Number of total/unique/observed reflections	13,538, 3403, 2321
No. of parameters	200
Independent reflections, R_int_	3403, 0.0482
R_all_, R_obs_	0.0879, 0.0544
wR2_all_, wR2_obs_	0.1425, 0.1221
Δρ_max, min_ (eÅ^−3^)	0.541, −0.269
goodness-of-fit (G.o.F)	1.029

**Table 3:** List of intermolecular interactions in the crystal structure of 2-(3,4-dimethoxyphenyl)-2,3-dihydroquinazolin-4(1*H*)-one (**3g**)**.**

Interaction	H···A (Å)	D···A (Å)	∠D–H···A (°)	Symmetry Code
N1–H1···O2	2.30	3.176	170	−x + 1, −y, −z + 2
N2–H2···O3	2.30	3.070	148	−x, −y, −z + 2
C16–H16C···O1	2.66	3.441	137	−x, −y, −z + 2
C3–H3···O1	2.54	3.167	124	x + 1,+y,+z
C2–H2C···O1	2.57	3.180	122	x + 1,+y,+z
C2–H2C···O3	2.64	3.421	140	−x + 1, −y, −z + 2

**Table 4:** Mortality of *Anopheles arabiensis* larvae exposed to test compounds **3a**–**n** at 4 µg/mL.

Compound	24-h Mortality	48-h Mortality
**3a** (4-OCH_3_)	0.77 ± 0.04 ^d,e,f^	0.83 ± 0.04 ^c,d,e^
**3b** (4-CN)	0.86 ± 0.04 ^b,c,d,e^	0.88 ± 0.03 ^b,c,d^
**3c** (3-OH)	0.51 ± 0.05 ^h,i,j,k,l,m^	0.57 ± 0.05 ^h,i,j,k,l^
**3d** (4-OCF_3_)	0.91 ± 0.03 ^b,c^	0.93 ± 0.03 ^a,b^
**3e** (3-I)	0.82 ± 0.04 ^c,d,e^	0.87 ± 0.04 ^b,c,d^
**3f** (4-Cl)	0.87 ± 0.04 ^b,c,d^	0.91 ± 0.03 ^b,c^
**3g** (3,4-diOCH_3_)	0.62 ± 0.05 ^g,h,i,j^	0.66 ± 0.05 ^f,g,h^
**3h** (4-N(CH_3_)_2_)	0.41 ± 0.05 ^m^	0.43 ± 0.05 ^l,m^
**3i** (3-NO_2_)	0.52 ± 0.05 ^h,i,j,k,l,m^	0.54 ± 0.05 ^h,i,j,k,l,m^
**3j** (4-F)	0.89 ± 0.03 ^b,c^	0.91 ± 0.03 ^b,c^
**3k** (imidazole)	0.46 ± 0.05 ^k,l,m^	0.48 ± 0.05 ^j,k,l,m^
**3l** (2-OCH_3_, 4-NO_2_)	0.74 ± 0.05 ^e,f,g^	0.77 ± 0.04 ^d,e,f^
**3m** (2-OH, 5-NO_2_)	0.59 ± 0.05 ^h,i,j,k^	0.64 ± 0.05 ^f,g,h,i^
**3n** (4-OH)	0.50 ± 0.05 ^i,j,k,l,m^	0.52 ± 0.05 ^h,i,j,k,l,m^
Temephos	0.99 ± 0.01 ^a^	1.00 ± 0.00 ^a^
Acetone	0.01 ± 0.01 ^m^	0.04 ± 0.02 ^n^

Means ± standard errors are shown. ^a–m^ Compounds sharing a letter do not differ significantly (*p* > 0.05).

**Table 5:** In silico ADMET properties of quinazolines (**3a–n)** and Temephos.

Entry	Solubility Level	Blood–Brain Barrier Penetration (BBB) Level	CYP450 Inhibition	Hepatotoxicity	Intestinal Absorption Level	Plasma Protein Binding	AlogP	PSA 2D
Temephos	2	0	-	+	0	+	5.656	53.58
**3a** (4-OCH_3_)	3	2	-	+	0	+	2.432	51.851
**3b** (4-CN)	3	2	-	+	0	+	2.327	65.856
**3c** (3-OH)	3	2	-	+	0	+	2.206	63.736
**3d** (4-OCF_3_)	1	1	-	+	0	+	4.568	51.851
**3e** (3-I)	2	1	-	+	0	+	3.026	42.921
**3f** (4-Cl)	2	1	-	+	0	+	3.113	42.921
**3g** (3,4-diOCH_3_)	3	2	-	+	0	+	2.415	60.781
**3h** (4-N(CH_3_)_2_)	3	2	-	+	0	+	2.610	46.273
**3i** (3-NO_2_)	3	3	-	+	0	+	2.343	85.744
**3j** (4-F)	3	2	-	+	0	+	2.654	42.921
**3k** (imidazole)	4	3	-	+	0	-	0.632	69.237
**3l** (2-OCH_3_, 4-NO_2_)	3	2	-	+	0	+	2.326	94.674
**3m** (2-OH, 5-NO_2_)	3	3	-	+	0	+	2.101	106.559
**3n** (4-OH)	3	2	-	+	0	+	2.206	63.736

Criteria: solubility level/drug-likeness: 0, extremely low/no; 1, very low/possible; 2, low/yes; 3, good/yes; 4, optimal/yes. BBB level (blood–brain barrier): 0, very high penetrant; 1, high; 2, medium; 3, low; 4, undefined. HIA level (human intestinal absorption): 0, good; 1, moderate; 2, poor; 3, very poor. PBB: plasma protein binding.

**Table 6:** Binding energy and residue interactions of 2,3-dihydroquinazolin-4(1*H*)-one derivatives (**3a**–**n)** against G199S mutant acetylcholinesterase 1 (AChE1) (Protein Data Bank (PDB) code: 6ARY).

Entry	Larvicidal Activity	Stereoisomer	Binding Energy (kJ/mol)	Residues Interaction	
				H-Bond (Interacting Atom)	Hydrophobic (π–π)
Native ligand	-	-	−46.68	Ser 280, Tyr 282	Gly 279, Trp 285, Tyr 489
Temephos	-	-	0	-	-
**3a** (4-OCH_3_)	0.77	*R*	−93.21	Trp 245 (NH), Tyr 489 (NHCO)	Trp 245, His 600
		*S*	−114.50	Gly 279 (NHCO), Ser 280 (NHCO) Tyr 489 (NH)	Tyr 282, Trp 245, Tyr 489
**3b** (4-CN)	0.86	*R*	−98.03	Ser 283 (NHCO), Tyr 489 (NH) Tyr 291 (CN)	Trp 245, Tyr 282, Asp 233 (ion-pi)
		*S*	−81.74	Trp 245 (NH), Tyr 291 (CN) Gly 278 (CN), Tyr 282 (NHCO) Tyr 489 (NHCO)	Trp 245
**3c** (3-OH)	0.51	*R*	−99.16	Ser 289 (NHCO), Tyr 291 (OH) Glu 359 (OH)	Trp 245, Tyr 489
		*S*	−89.42	Ser 280 (OH), Tyr 282 (OH) Ser 283 (NHCO), Tyr 289 (NH)	Trp 245, Tyr 489
**3d** (4-OCF_3_)	0.91	*R*	−110.82	Gly 278 (CF_3_), Tyr 291 (CF_3_) Tyr 489 (NH)	Trp 245, Tyr 282, Asp 233 (ion-pi), Glu 359 (F), Gly 278 (F)
		*S*	−106.47	Ser 283 (NHCO) Tyr 489 (NH)	Trp 245, Tyr 282, Tyr 489
**3e** (3-I)	0.82	*R*	−105.04	Tyr 489 (NH)	Trp 245, Tyr 282, Tyr 489, Asn 246 (I)
		*S*	−75.88	Trp 245 (NH)	Trp 245
**3f** (4-Cl)	0.87	*R*	−108.19	Ser 283 (NHCO) Tyr 489 (NH)	Tyr 282, Trp 245, Asp 233 (ion-pi)
		*S*	−81.59	Trp 245 (NH), Tyr 282 (NHCO) Tyr 489 (NHCO), Glu 359 (Cl)	Tyr 245
**3g** (3,4-diOCH_3_)	0.62	*R*	−95.90	Ser 283 (NHCO) Tyr 489 (NH)	Trp 245, Tyr 282, Tyr 489
		*S*	−91.29	Trp 245 (NH), Tyr 282 (NHCO) Tyr 489 (NHCO)	Tyr 245
**3h** (4-N(CH_3_)_2_)	0.41	*R*	−92.98	Tyr 489 (NH)	Trp 245, Tyr 282, Tyr 489
		*S*	−91.01	Ser 283 (NHCO) Tyr 489 (NH)	Trp 245, Tyr 489
**3i** (3-NO_2_)	0.52	*R*	−108.38	Asp 233 (NO_2_), Ser 283 (NHCO) Tyr 489 (NH)	Trp 245, Tyr 282, Tyr 489
		*S*	−131.24	Ser 280 (NHCO) Tyr 489 (NH)	Trp 245, Tyr 282
**3j** (4-F)	0.89	*R*	−101.15	Ser 283 (NHCO) Tyr 489 (NH)	Trp 245, Tyr 489
		*S*	−76.85	Tyr 291 (NHCO) Tyr 489 (NH)	Trp 245, Tyr 489
**3k** (imidazole)	0.46	*R*	−91.64	Ser 283 (NHCO) Tyr 489 (NH)	Trp 245
		*S*	−80.14	Ser 283 (NHCO) Tyr 489 (NH)	Trp 245, Tyr 489
**3l** (2-OCH_3_, 4-NO_2_)	0.74	*R*	−120.40	Gly 279 (NHCO), Ser 289 (NHCO) Ser 289 (OCH_3_)	Trp 245, Tyr 489, His 600, Glu 359 (ion-pi)
		*S*	−89.15	Ser 283 (NHCO) Tyr 489 (NH)	Trp 245, Tyr 282, Tyr 489, Tyr 493 (ion-pi)
**3m** (2-OH, 5-NO_2_)	0.59	*R*	−127.01	Ser 283 (NHCO), Tyr 489 (NH) Tyr 489 (OH)	Trp 245, Tyr 282, Tyr 489
		*S*	−113.14	Trp 245 (NH) Ser 283 (OH)	Trp 245, Glu 359 (ion-pi), His 600 (ion-pi)
**3n** (4-OH)	0.50	*R*	−91.36	Ser 280 (NHCO), Tyr 291 (OH) Glu 359 (OH)	Trp 245, Tyr 282, Tyr 489
		*S*	−121.58	Ser 280 (NHCO), Tyr 291 (OH) Glu 359 (OH)	Trp 245

## Supplementary Materials

The following are available online at https://www.mdpi.com/1420-3049/25/6/1316/s1: Figure S1: FT-IR of 2-(4-methoxyphenyl)-2, 3-dihydroquinazolin-4(1*H*)-one (3**a**); Figure S2: ^1^H-NMR of 2-(4-methoxyphenyl)-2, 3-dihydroquinazolin-4(1*H*)-one (3**a**); Figure S3: ^13^C-NMR of 2-(4-methoxyphenyl)-2, 3-dihydroquinazolin-4(1*H*)-one (3**a**); Figure S4: FT-IR of 4-(4-oxo-1,2,3,4-tetrahydroquinazolin-2-yl)benzonitrile (3**b**); Figure S5: ^1^H-NMR of 4-(4-oxo-1,2,3,4-tetrahydroquinazolin-2-yl)benzonitrile (3**b**); Figure S6: ^13^C-NMR of 4-(4-oxo-1,2,3,4-tetrahydroquinazolin-2-yl)benzonitrile (3**b**); Figure S7: FT-IR of 2-(3-hydroxyphenyl)-2,3-dihydroquinazolin-4(1*H*)-one (3**c**); Figure S8: ^1^H-NMR of 2-(3-hydroxyphenyl)-2,3-dihydroquinazolin-4(1*H*)-one (3**c**); Figure S9: ^13^C-NMR of 2-(3-hydroxyphenyl)-2,3-dihydroquinazolin-4(1*H*)-one (3**c**); Figure S10: FT-IR of 2-(4-(trifluoromethoxy)phenyl)-2,3-dihydroquinazolin-4(1*H*)-one (3**d**); Figure S11: ^1^H-NMR of 2-(4-(trifluoromethoxy)phenyl)-2,3-dihydroquinazolin-4(1*H*)-one (3**d**); Figure S12: ^13^C-NMR of 2-(4-(trifluoromethoxy)phenyl)-2,3-dihydroquinazolin-4(1*H*)-one (3**d**); Figure S13: FT-IR of 2-(3-iodophenyl)-2,3-dihydroquinazolin-4(1*H*)-one (3**e**); Figure S14: ^1^H-NMR of 2-(3-iodophenyl)-2,3-dihydroquinazolin-4(1*H*)-one (3**e**); Figure S15: ^13^C-NMR of 2-(3-iodophenyl)-2,3-dihydroquinazolin-4(1*H*)-one (3**e**); Figure S16: FT-IR of 2-(4-chlorophenyl)-2,3-dihydroquinazolin-4(1*H*)-one (3**f**); Figure S17: ^1^H-NMR of 2-(4-chlorophenyl)-2,3-dihydroquinazolin-4(1*H*)-one (3**f**); Figure S18: ^13^C-NMR of 2-(4-chlorophenyl)-2,3-dihydroquinazolin-4(1*H*)-one (3**f**); Figure S19: FT-IR of 2-(3,4-dimethoxyphenyl)-2,3-dihydroquinazolin-4(1*H*)-one (3**g**); Figure S20: ^1^H-NMR of 2-(3,4-dimethoxyphenyl)-2,3-dihydroquinazolin-4(1*H*)-one (3**g**); Figure S21: ^13^C-NMR of 2-(3,4-dimethoxyphenyl)-2,3-dihydroquinazolin-4(1*H*)-one (3**g**); Figure S22: FT-IR of 2-(4-(dimethylamino)phenyl)-2,3-dihydroquinazolin-4(1*H*)-one (3**h**); Figure S23: ^1^H-NMR of 2-(4-(dimethylamino)phenyl)-2,3-dihydroquinazolin-4(1*H*)-one (3**h**); Figure S24: ^13^C-NMR of 2-(4-(dimethylamino)phenyl)-2,3-dihydroquinazolin-4(1*H*)-one (3h); Figure S25: FT-IR of 2-(3-nitrophenyl)-2,3-dihydroquinazolin-4(1*H*)-one (3**i**); Figure S26: ^1^H-NMR of 2-(3-nitrophenyl)-2,3-dihydroquinazolin-4(1*H*)-one (3**i**); Figure S27: ^13^C-NMR of 2-(3-nitrophenyl)-2,3-dihydroquinazolin-4(1*H*)-one (3**i**); Figure S28: FT-IR of 2-(4-fluorophenyl)-2,3-dihydroquinazolin-4(1*H*)-one (3**j**); Figure S29: ^1^H-NMR of 2-(4-fluorophenyl)-2,3-dihydroquinazolin-4(1*H*)-one (3**j**); Figure S30: ^13^C-NMR of 2-(4-fluorophenyl)-2,3-dihydroquinazolin-4(1*H*)-one (3**j**); Figure S31: FT-IR of 2-(1H-imidazol-4-yl)-2,3-dihydroquinazolin-4(1*H*)-one (3**k**); Figure S32: ^1^H-NMR of 2-(1H-imidazol-4-yl)-2,3-dihydroquinazolin-4(1*H*)-one (3**k**); Figure S33: ^13^C-NMR of 2-(1H-imidazol-4-yl)-2,3-dihydroquinazolin-4(1*H*)-one (3**k**); Figure S34: FT-IR of 2-(2-methoxy-4-nitrophenyl)-2,3-dihydroquinazolin-4(1*H*)-one (3**l**); Figure S35: ^1^H-NMR of 2-(2-methoxy-4-nitrophenyl)-2,3-dihydroquinazolin-4(1*H*)-one (3**l**); Figure S36: ^13^C-NMR of 2-(2-methoxy-4-nitrophenyl)-2,3-dihydroquinazolin-4(1*H*)-one (3**l**); Figure S37: FT-IR of 2-(2-hydroxy-5-nitrophenyl)-2,3-dihydroquinazolin-4(1*H*)-one (3**m**); Figure S38: ^1^H-NMR of 2-(2-hydroxy-5-nitrophenyl)-2,3-dihydroquinazolin-4(1*H*)-one (3**m**); Figure S39: ^13^C-NMR of 2-(2-hydroxy-5-nitrophenyl)-2,3-dihydroquinazolin-4(1*H*)-one (3**m**); Figure S40: FT-IR of 2-(4-hydroxyphenyl)-2,3-dihydroquinazolin-4(1*H*)-one (3**n**); Figure S41: ^1^H-NMR of 2-(4-hydroxyphenyl)-2,3-dihydroquinazolin-4(1*H*)-one (3**n**); Figure S42: ^13^C-NMR of 2-(4-hydroxyphenyl)-2,3-dihydroquinazolin-4(1*H*)-one (3**n**).
