# Supplementary material for: Larvicidal Activities of 2-Aryl-2,3-Dihydroquinazolin -4-ones against Malaria Vector Anopheles arabiensis, In Silico ADMET Prediction and Molecular Target Investigation
Source: Molecules. 2020 Mar 13;25(6):1316. doi: 10.3390/molecules25061316 (PMC7144721; doi:10.3390/molecules25061316)

Larvicidal Activities of 2-Aryl-2,3-Dihydroquinazolin-4-ones Against Malaria Vector *Anopheles arabiensis*, In Silico ADMET Prediction and Molecular Target Investigation

Katharigatta N. Venugopala [1,2,*], Pushpalatha Ramachandra [3], Christophe Tratrat [1], Raquel M. Gleiser [4], Subhrajyoti Bhandary [5], Deepak Chopra [5], Mohamed A. Morsy [1,6], Bandar E. Aldhubiab[1], Mahesh Attimarad [1], Anroop B. Nair [1], Nagaraja Sreeharsha [1], Rashmi Venugopala [7], Pran Kishore Deb [8], Sandeep Chandrashekharappa [9], Hany Ezzat Khalil [1,10], Osama I. Alwassil [11], Sara Nidal Abed [8], Yazan A. Bataineh [8], Ramachandra Palenge [3], Michelyne Haroun [1], Shinu Pottathil [13], Meravanige B. Girish [14], Sabah H. Akrawi [1] and Viresh Mohanlall [2]

[1] Department of Pharmaceutical Sciences, College of Clinical Pharmacy, King Faisal University, Al-Ahsa 31982, Kingdom of Saudi Arabia; momorsy@kfu.edu.sa (M.A.M.); baldhubiab@kfu.edu.sa (B.E.A.); mattimarad@kfu.edu.sa (M.A.); anair@kfu.edu.sa (A.B.N.); sharsha@kfu.edu.sa (N.S.); heahmed@kfu.edu.sa (H.E.K.); mharoun@kfu.edu.sa (M.H.); sakrawi@kfu.edu.sa (S.H.A.)

[2] Department of Biotechnology and Food Technology, Durban University of Technology, Durban 4001, South Africa; vireshm@dut.ac.za (V.M.)

[3] Department of Chemistry, School of Applied Sciences, REVA University, Bangalore 560 064, India; pushpalatha@reva.edu.in (P.R.); ramachandrap@reva.edu.in (R.P.)

[4] CREAN-IMBIV (CONICET-UGC), AV Valparaiso s.n., and FCEFyN, AV. Sarsfield 299, Universidad Nacional de Cordoba, Cordoba 5000, Argentina; raquel.gleiser@unc.edu.ar (R.M.G.)

[5] Department of Chemistry, Indian Institute of Science Education and Research Bhopal, Indore By-pass Road, Bhauri, Bhopal 462 066, Madhya Pradesh, India; bhandarysj@gmail.com (S.B.); dchopra@iiserb.ac.in (D.C.)

[6] Department of Pharmacology, Faculty of Medicine, Minia University, El-Minia 61511, Egypt

[7] Department of Public Health Medicine, University of KwaZulu-Natal, Howard College Campus, Durban 4001, South Africa; venugopalar@ukzn.ac.za (R.V.)

[8] Department of Pharmaceutical Sciences, Faculty of Pharmacy, Philadelphia University, PO Box 1, Amman 19392, Jordan; pdeb@philadelphia.edu.jo (P.K.D.); sousanidal95@gmail.com (S.N.A.); YBatineh@philadelphia.edu.jo (Y.A.B.)

[9] Institute for Stem Cell Biology and Regenerative Medicine, NCBS, TIFR, GKVK, Bellary Road, Bangalore 560 065, India; sandeepc@instem.res.in (S.C.)

[10] Department of Pharmacognosy, Faculty of Pharmacy, Minia University, Minia 61519, Egypt

[11] Department of Pharmaceutical Sciences, College of Pharmacy, King Saud bin Abdulaziz University for Health Sciences, Riyadh 11481, Kingdom of Saudi Arabia; wassilo@ksau-hs.edu.sa

[12] Department of Pharmaceutical Chemistry, Shri Vishnu College of Pharmacy, Vishnupur, Bhimavaram 534 202, West Godavari Dist., Andhra Pradesh, India; raghumrp@svcp.edu.in

[13] Department of Biomedical Sciences, College of Clinical Pharmacy, King Faisal University, Al-Ahsa 31982, Kingdom of Saudi Arabia; spottathail@kfu.edu.sa (S.P.)

[14] Department of Biomedical Sciences, College of Medicine, King Faisal University, Al-Ahsa 31982, Kingdom of Saudi Arabia; gmeravanige@kfu.edu.sa (M.B.G.)

* Correspondence: kvenugopala@kfu.edu.sa (K.N.V.)

TABLE OF CONTENTS

Figures	Contents
**S1**	FT-IR of 2-(4-methoxyphenyl)-2, 3-dihydroquinazolin-4(1*H*)-one (**3a**)
**S2**	^{1}H-NMR of 2-(4-methoxyphenyl)-2, 3-dihydroquinazolin-4(1*H*)-one (**3a**)
**S3**	^{13}C-NMR of 2-(4-methoxyphenyl)-2, 3-dihydroquinazolin-4(1*H*)-one (**3a**)
**S4**	FT-IR of 4-(4-oxo-1,2,3,4-tetrahydroquinazolin-2-yl)benzonitrile (**3b**)
**S5**	^{1}H-NMR of 4-(4-oxo-1,2,3,4-tetrahydroquinazolin-2-yl)benzonitrile (**3b**)
**S6**	^{13}C-NMR of 4-(4-oxo-1,2,3,4-tetrahydroquinazolin-2-yl)benzonitrile (**3b**)
**S7**	FT-IR of 2-(3-hydroxyphenyl)-2,3-dihydroquinazolin-4(1*H*)-one (**3c**)
**S8**	^{1}H-NMR of 2-(3-hydroxyphenyl)-2,3-dihydroquinazolin-4(1*H*)-one (**3c**)
**S9**	^{13}C-NMR of 2-(3-hydroxyphenyl)-2,3-dihydroquinazolin-4(1*H*)-one (**3c**)
**S10**	FT-IR of 2-(4-(trifluoromethoxy)phenyl)-2,3-dihydroquinazolin-4(1*H*)-one (**3d**)
**S11**	^{1}H-NMR of 2-(4-(trifluoromethoxy)phenyl)-2,3-dihydroquinazolin-4(1*H*)-one (**3d**)
**S12**	^{13}C-NMR of 2-(4-(trifluoromethoxy)phenyl)-2,3-dihydroquinazolin-4(1*H*)-one (**3d**)
**S13**	FT-IR of 2-(3-iodophenyl)-2,3-dihydroquinazolin-4(1*H*)-one (**3e**)
**S14**	^{1}H-NMR of 2-(3-iodophenyl)-2,3-dihydroquinazolin-4(1*H*)-one (**3e**)
**S15**	^{13}C-NMR of 2-(3-iodophenyl)-2,3-dihydroquinazolin-4(1*H*)-one (**3e**)
**S16**	FT-IR of 2-(4-chlorophenyl)-2,3-dihydroquinazolin-4(1*H*)-one (**3f**)

**S17**	^{1}H-NMR of 2-(4-chlorophenyl)-2,3-dihydroquinazolin-4(1*H*)-one (**3f**)
**S18**	^{13}C-NMR of 2-(4-chlorophenyl)-2,3-dihydroquinazolin-4(1*H*)-one (**3f**)
**S19**	FT-IR of 2-(3,4-dimethoxyphenyl)-2,3-dihydroquinazolin-4(1*H*)-one (**3g**)
**S20**	^{1}H-NMR of 2-(3,4-dimethoxyphenyl)-2,3-dihydroquinazolin-4(1*H*)-one (**3g**)
**S21**	^{13}C-NMR of 2-(3,4-dimethoxyphenyl)-2,3-dihydroquinazolin-4(1*H*)-one (**3g**)
**S22**	FT-IR of 2-(4-(dimethylamino)phenyl)-2,3-dihydroquinazolin-4(1*H*)-one (**3h**)
**S23**	^{1}H-NMR of 2-(4-(dimethylamino)phenyl)-2,3-dihydroquinazolin-4(1*H*)-one (**3h**),
**S24**	^{13}C-NMR of 2-(4-(dimethylamino)phenyl)-2,3-dihydroquinazolin-4(1*H*)-one (**3h**)
**S21**	FT-IR of 2-(3-nitrophenyl)-2,3-dihydroquinazolin-4(1*H*)-one (**3i**)
**S26**	^{1}H-NMR of 2-(3-nitrophenyl)-2,3-dihydroquinazolin-4(1*H*)-one (**3i**)
**S27**	^{13}C-NMR of 2-(3-nitrophenyl)-2,3-dihydroquinazolin-4(1*H*)-one (**3i**)
**S28**	FT-IR of 2-(4-fluorophenyl)-2,3-dihydroquinazolin-4(1*H*)-one (**3j**)
**S29**	^{1}H-NMR of 2-(4-fluorophenyl)-2,3-dihydroquinazolin-4(1*H*)-one (**3j**)
**S30**	^{13}C-NMR of 2-(4-fluorophenyl)-2,3-dihydroquinazolin-4(1*H*)-one (**3j**)
**S31**	FT-IR of 2-(1*H*-imidazol-4-yl)-2,3-dihydroquinazolin-4(1*H*)-one (**3k**)
**S32**	^{1}H-NMR of 2-(1*H*-imidazol-4-yl)-2,3-dihydroquinazolin-4(1*H*)-one (**3k**)

**S33**	^{13}C-NMR of 2-(1*H*-imidazol-4-yl)-2,3-dihydroquinazolin-4(1*H*)-one (**3k**)
**S34**	FT-IR of 2-(2-methoxy-4-nitrophenyl)-2,3-dihydroquinazolin-4(1*H*)-one (**3l**)
**S35**	^{1}H-NMR of 2-(2-methoxy-4-nitrophenyl)-2,3-dihydroquinazolin-4(1*H*)-one (**3l**)
**S36**	^{13}C-NMR of 2-(2-methoxy-4-nitrophenyl)-2,3-dihydroquinazolin-4(1*H*)-one (**3l**)
**S37**	FT-IR of 2-(2-hydroxy-5-nitrophenyl)-2,3-dihydroquinazolin-4(1*H*)-one (**3m**)
**S38**	^{1}H-NMR of 2-(2-hydroxy-5-nitrophenyl)-2,3-dihydroquinazolin-4(1*H*)-one (**3m**)
**S39**	^{13}C-NMR of 2-(2-hydroxy-5-nitrophenyl)-2,3-dihydroquinazolin-4(1*H*)-one (**3m**)
**S40**	FT-IR of 2-(4-hydroxyphenyl)-2,3-dihydroquinazolin-4(1*H*)-one (**3n**)
**S41**	^{1}H-NMR of 2-(4-hydroxyphenyl)-2,3-dihydroquinazolin-4(1*H*)-one (**3n**)
**S42**	^{13}C-NMR of 2-(4-hydroxyphenyl)-2,3-dihydroquinazolin-4(1*H*)-one (**3n**)

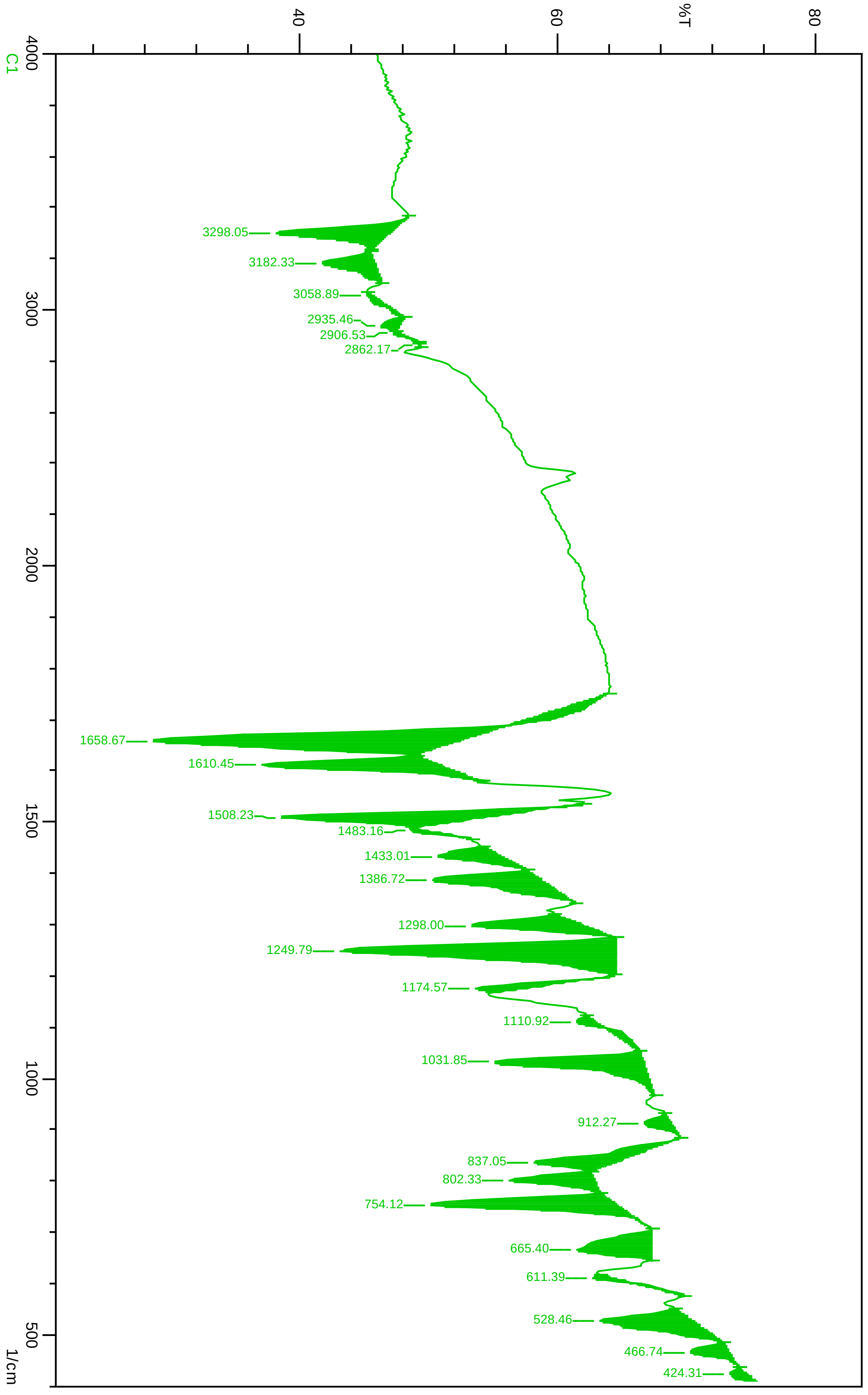

C1
%T
40
60
80
4000
3000
2000
1500
1000
500
1/cm
3298.05
3182.33
3058.89
2935.46
2906.53
2862.17
1658.67
1610.45
1508.23
1483.16
1433.01
1386.72
1298.00
1249.79
1174.57
1110.92
1031.85
912.27
837.05
802.33
754.12
665.40
611.39
528.46
466.74
424.31

PROTON DMSO {C:\Bruker\TOPSPIN\KFUCCP} nmr 12

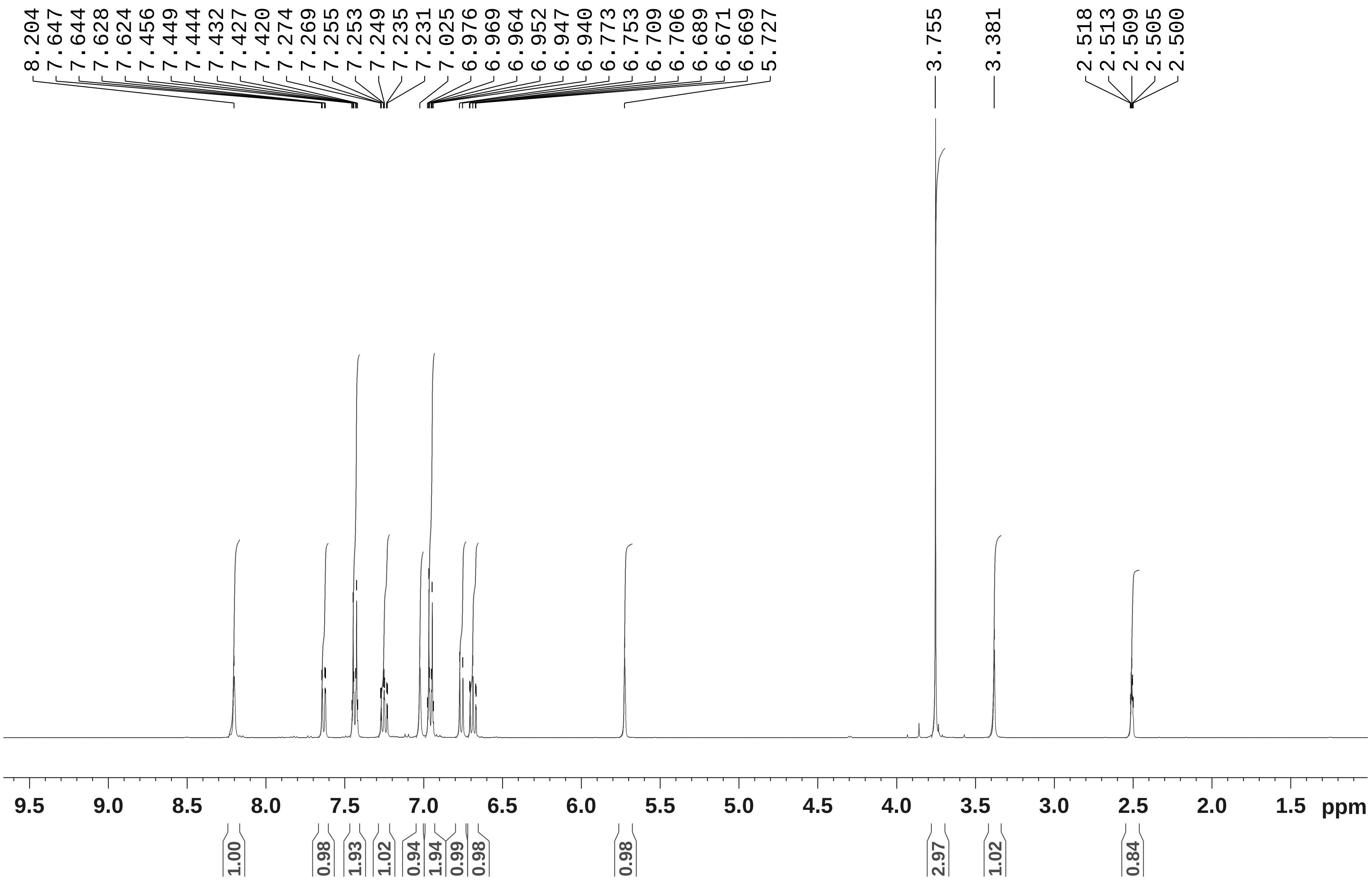

C13CPD DMSO {C:\Bruker\TOPSPIN\KFUCCP} nmr 12

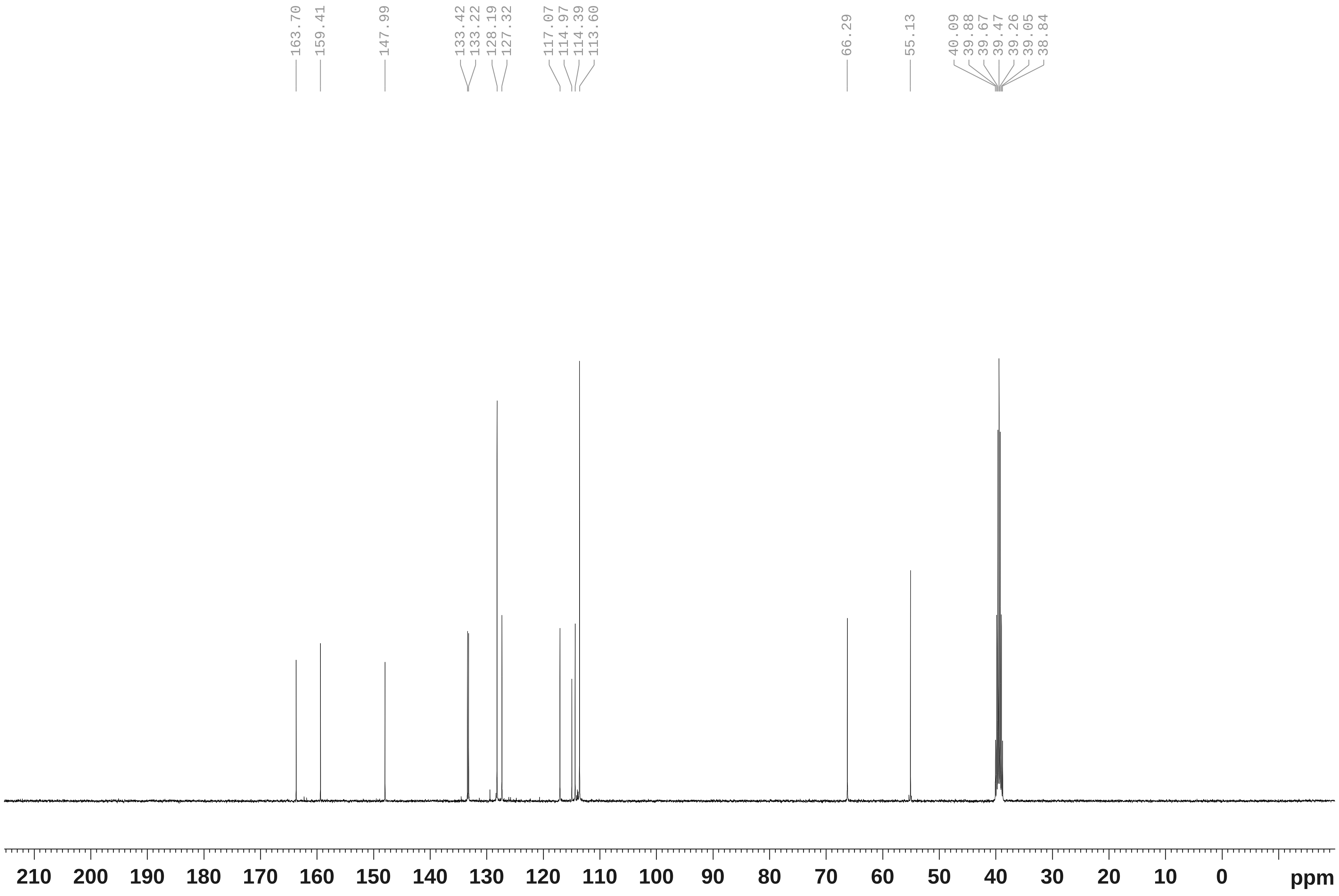

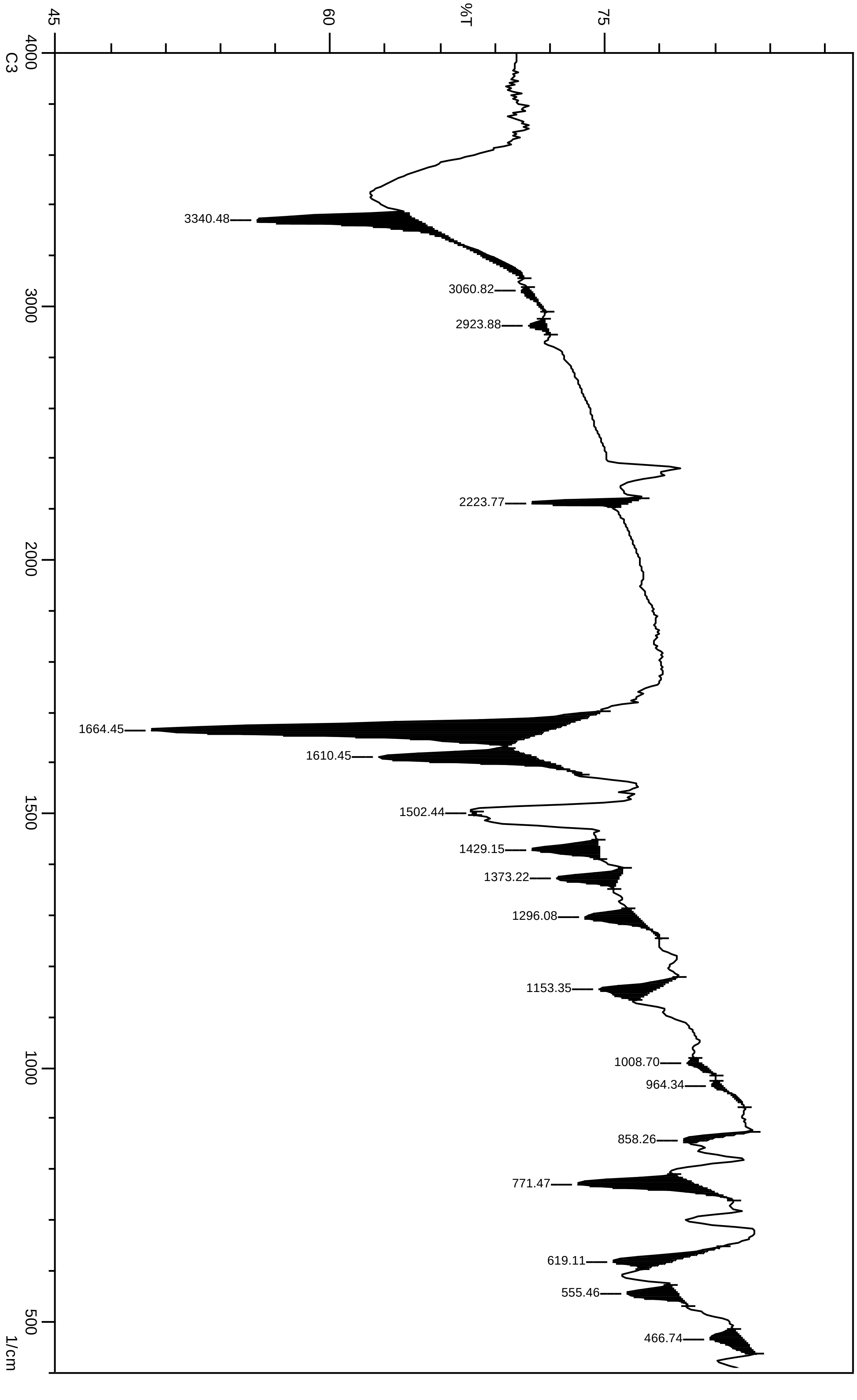

C3
%T
45
60
75
4000
3000
2000
1500
1000
500
1/cm
3340.48
3060.82
2923.88
2223.77
1664.45
1610.45
1502.44
1429.15
1373.22
1296.08
1153.35
1008.70
964.34
858.26
771.47
619.11
555.46
466.74

PROTON DMSO {C:\Bruker\TOPSPIN\KFUCCP} nmr 6

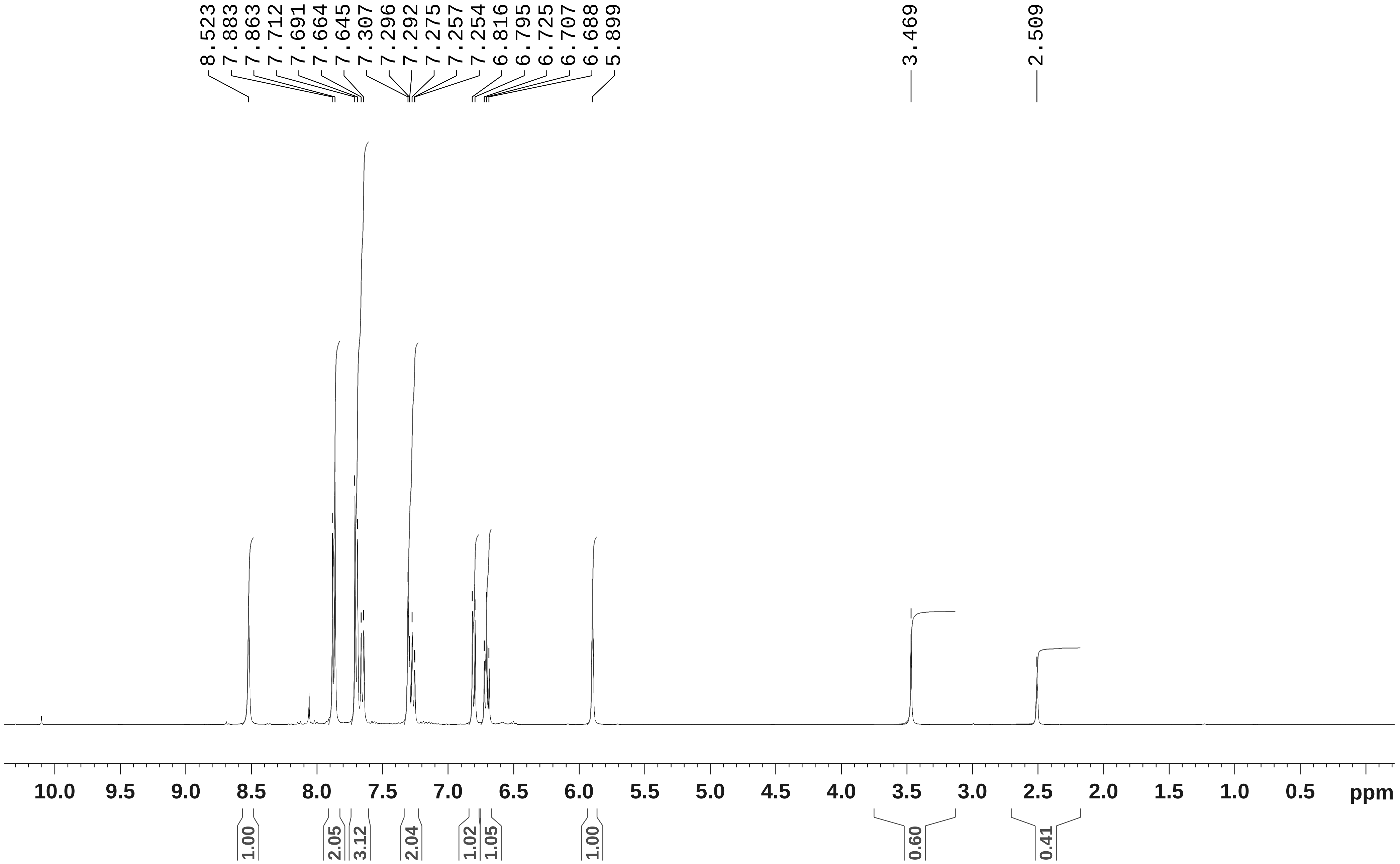

C13CPD DMSO {C:\Bruker\TOPSPIN\KFUCCP} nmr 6

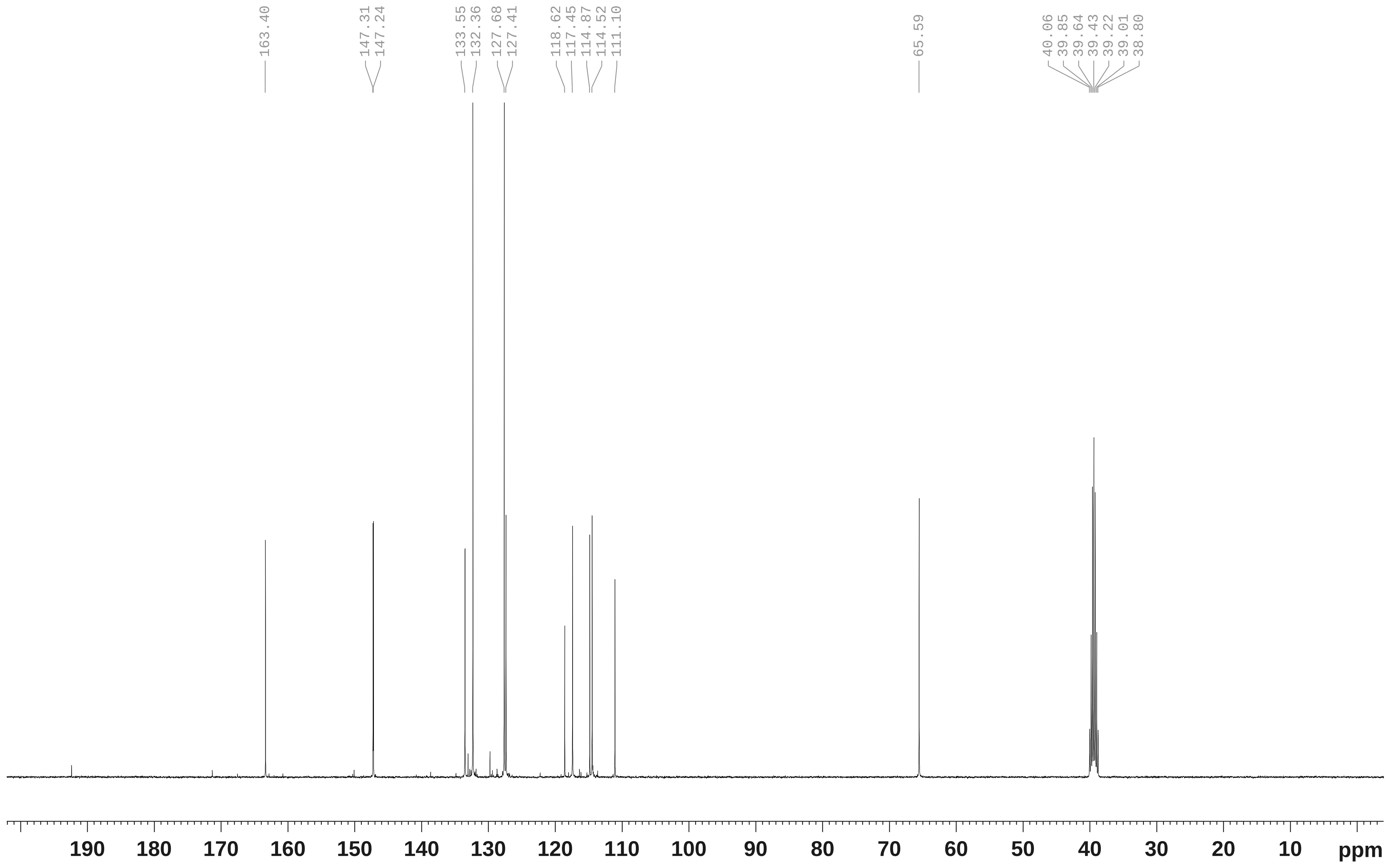

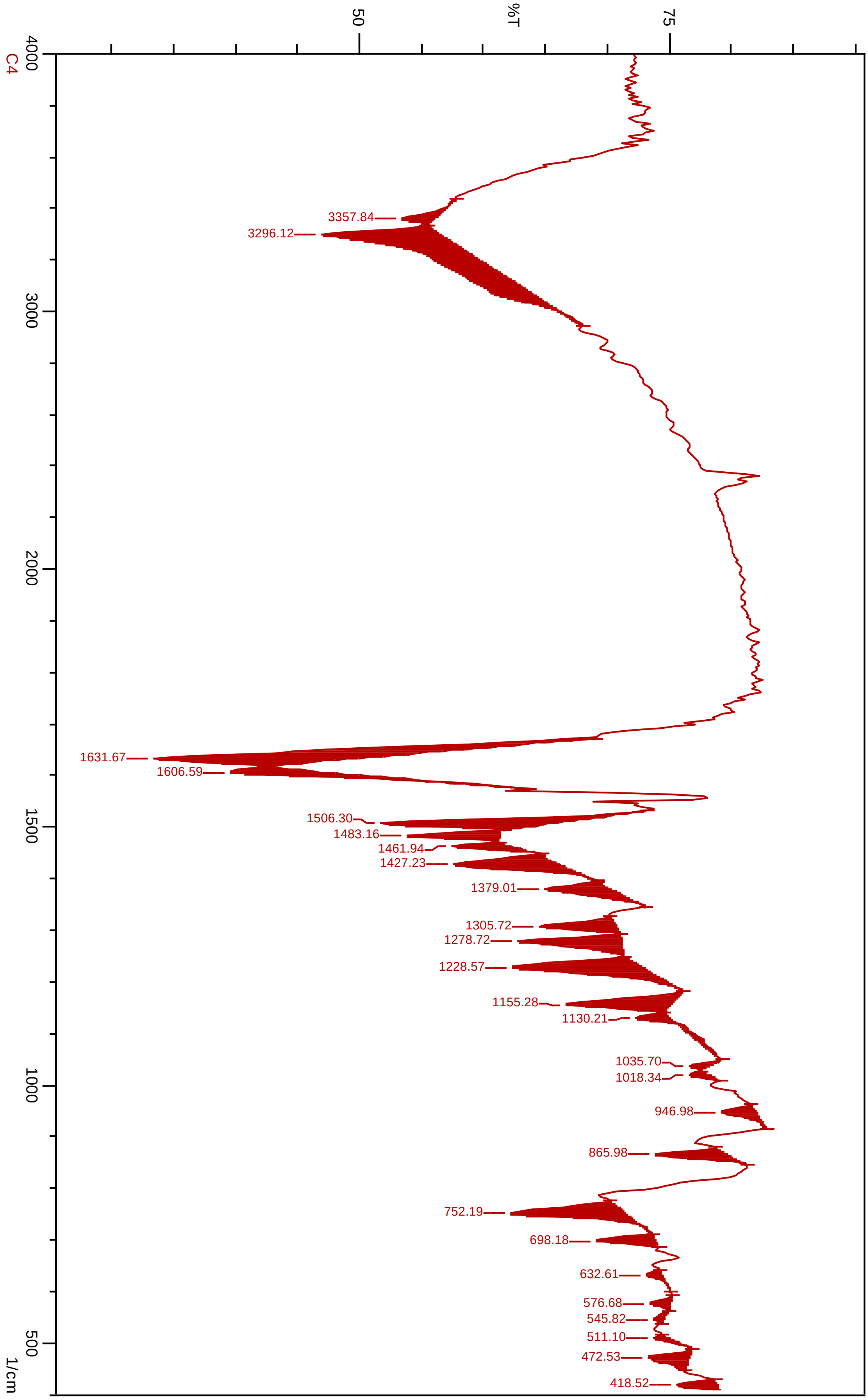
C4
%T
50
75
4000
3000
2000
1500
1000
500
1/cm
3357.84
3296.12
1631.67
1606.59
1506.30
1483.16
1461.94
1427.23
1379.01
1305.72
1278.72
1228.57
1155.28
1130.21
1035.70
1018.34
946.98
865.98
752.19
698.18
632.61
576.68
545.82
511.10
472.53
418.52

PROTON DMSO {C:\Bruker\TOPSPIN\KFUCCP} nmr 10

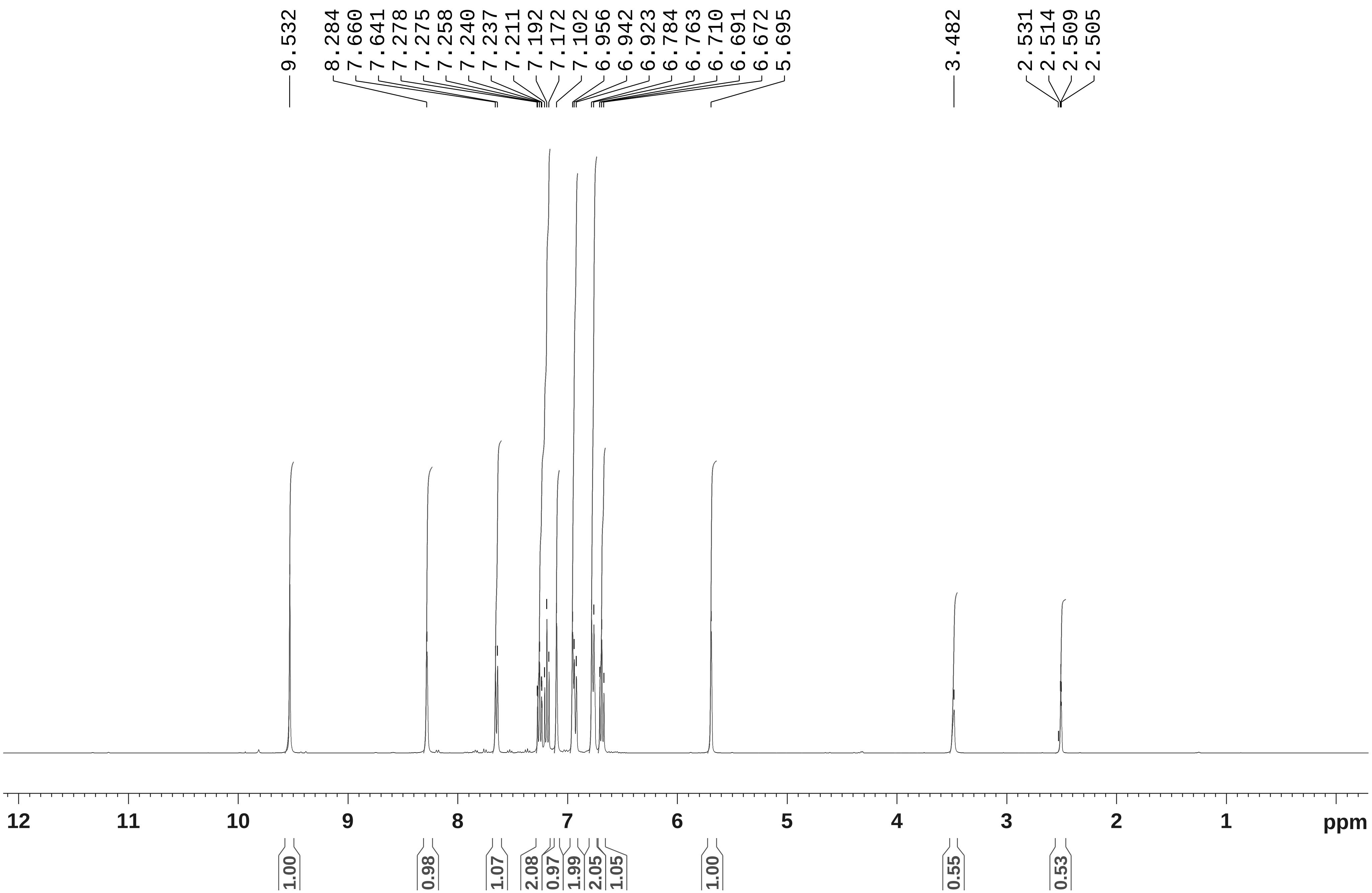

C13CPD DMSO {C:\Bruker\TOPSPIN\KFUCCP} nmr 10

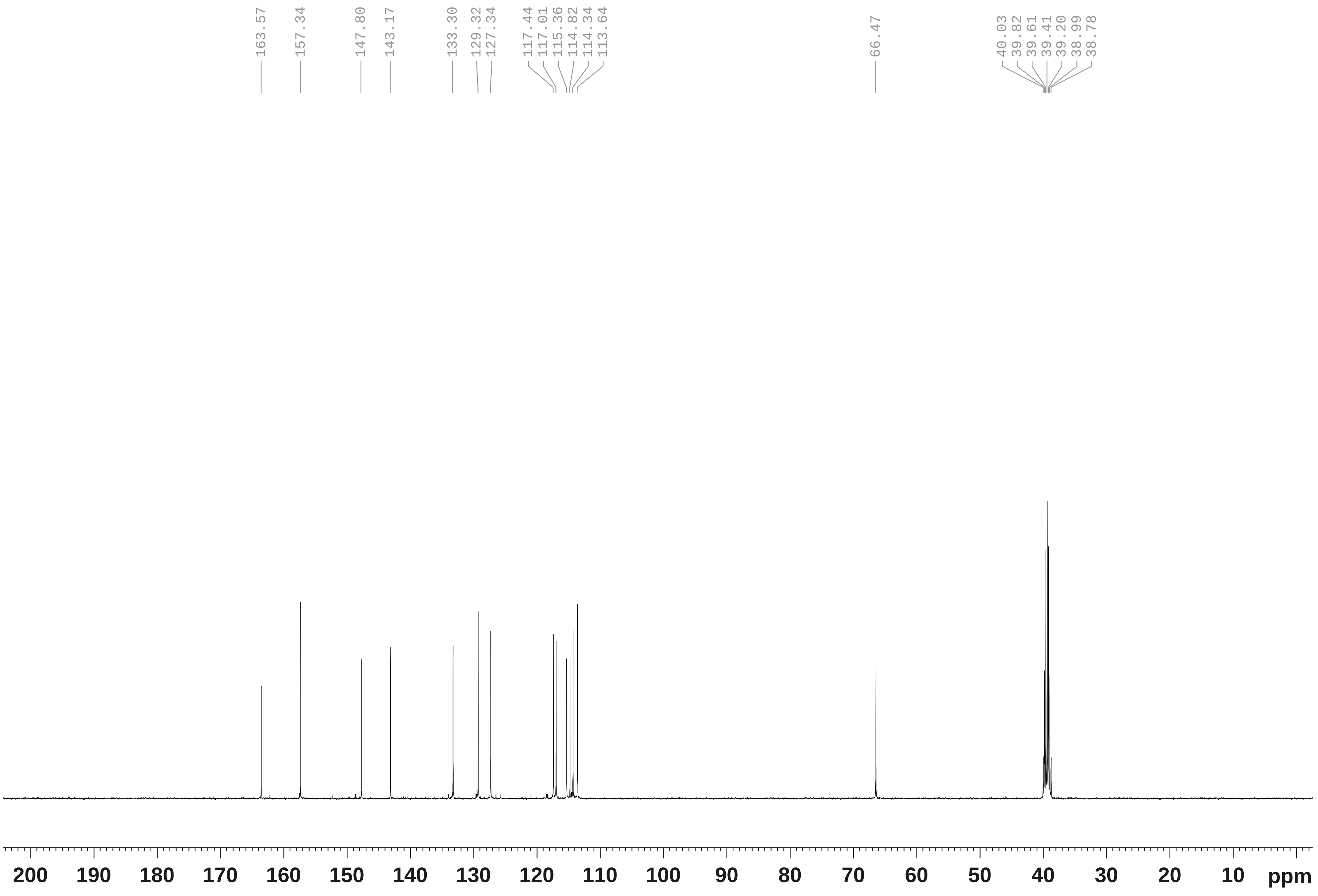

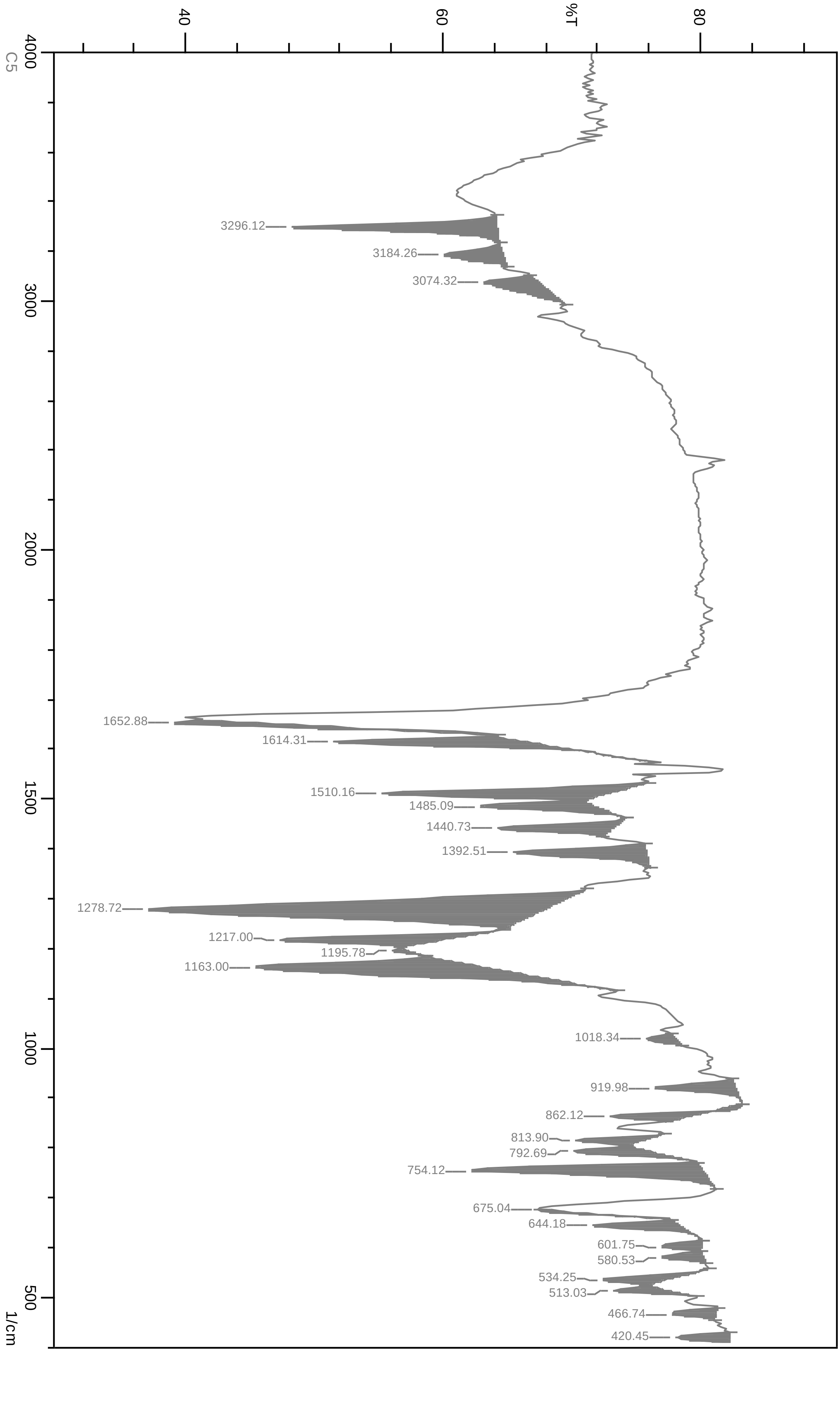
C5
%T
40
60
80
4000
3000
2000
1500
1000
500
1/cm
3296.12
3184.26
3074.32
1652.88
1614.31
1510.16
1485.09
1440.73
1392.51
1278.72
1217.00
1195.78
1163.00
1018.34
919.98
862.12
813.90
792.69
754.12
675.04
644.18
601.75
580.53
534.25
513.03
466.74
420.45

PROTON DMSO {C:\Bruker\TOPSPIN\KFUCCP} nmr 11

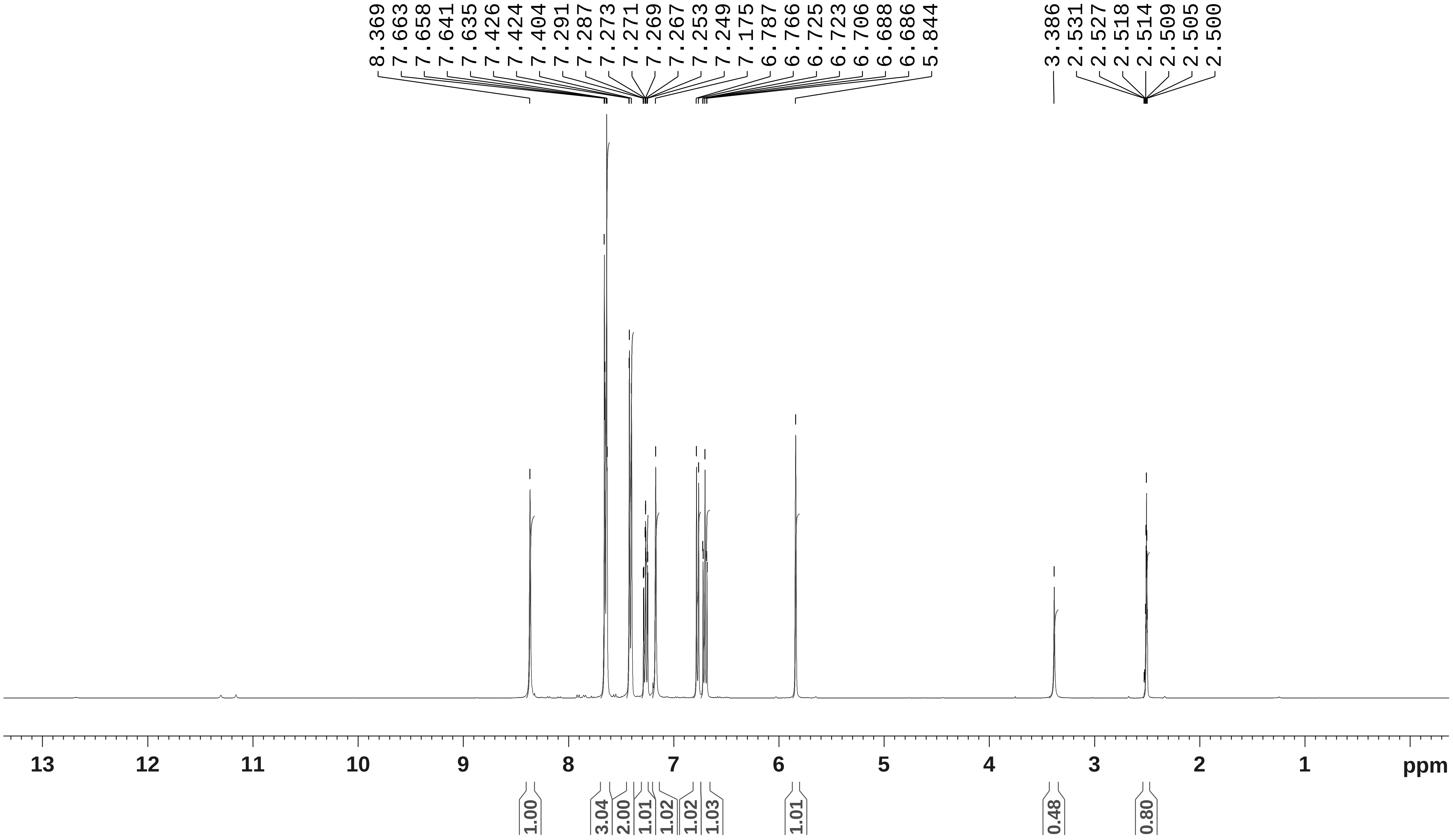

C13CPD DMSO {C:\Bruker\TOPSPIN\KFUCCP} nmr 11

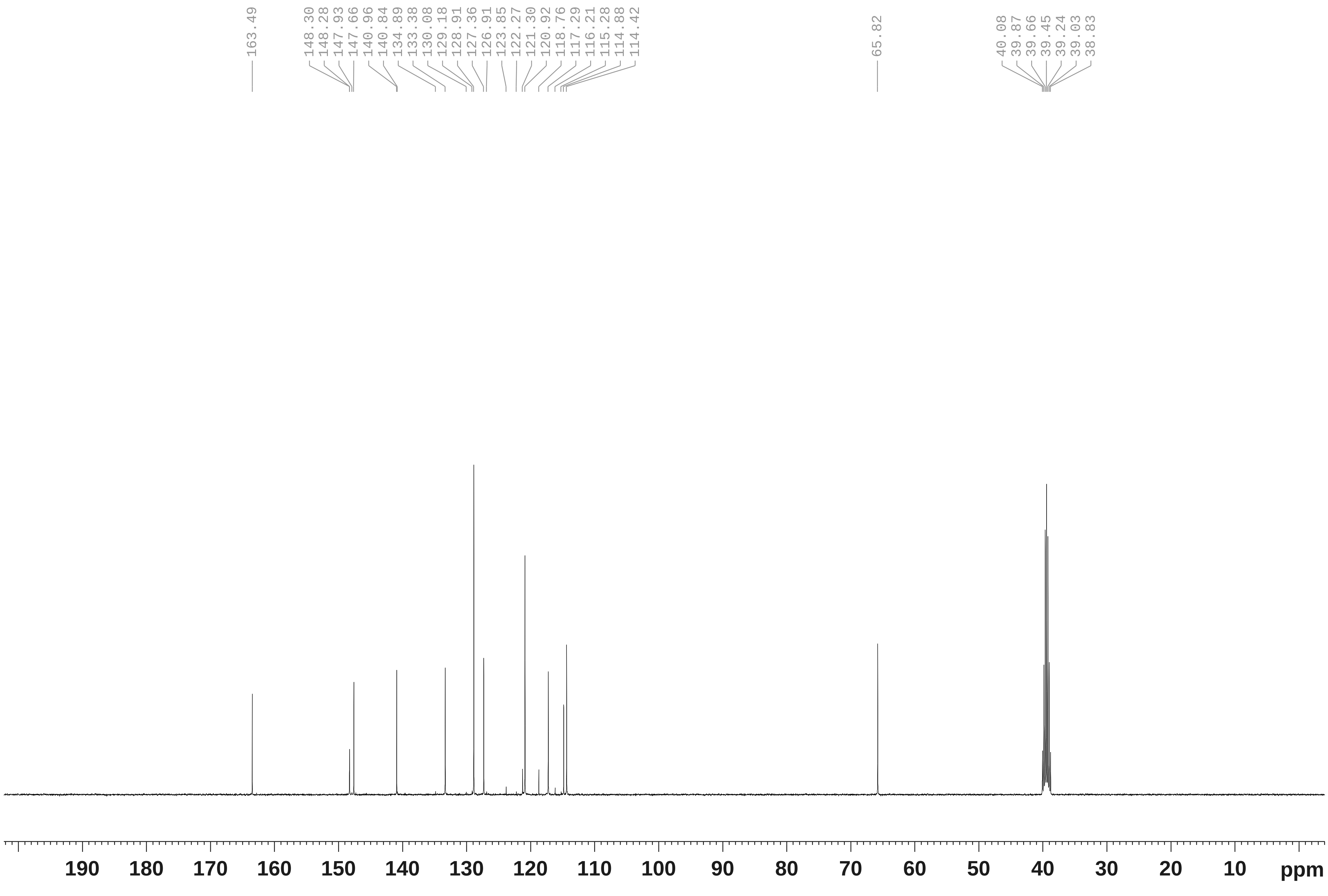

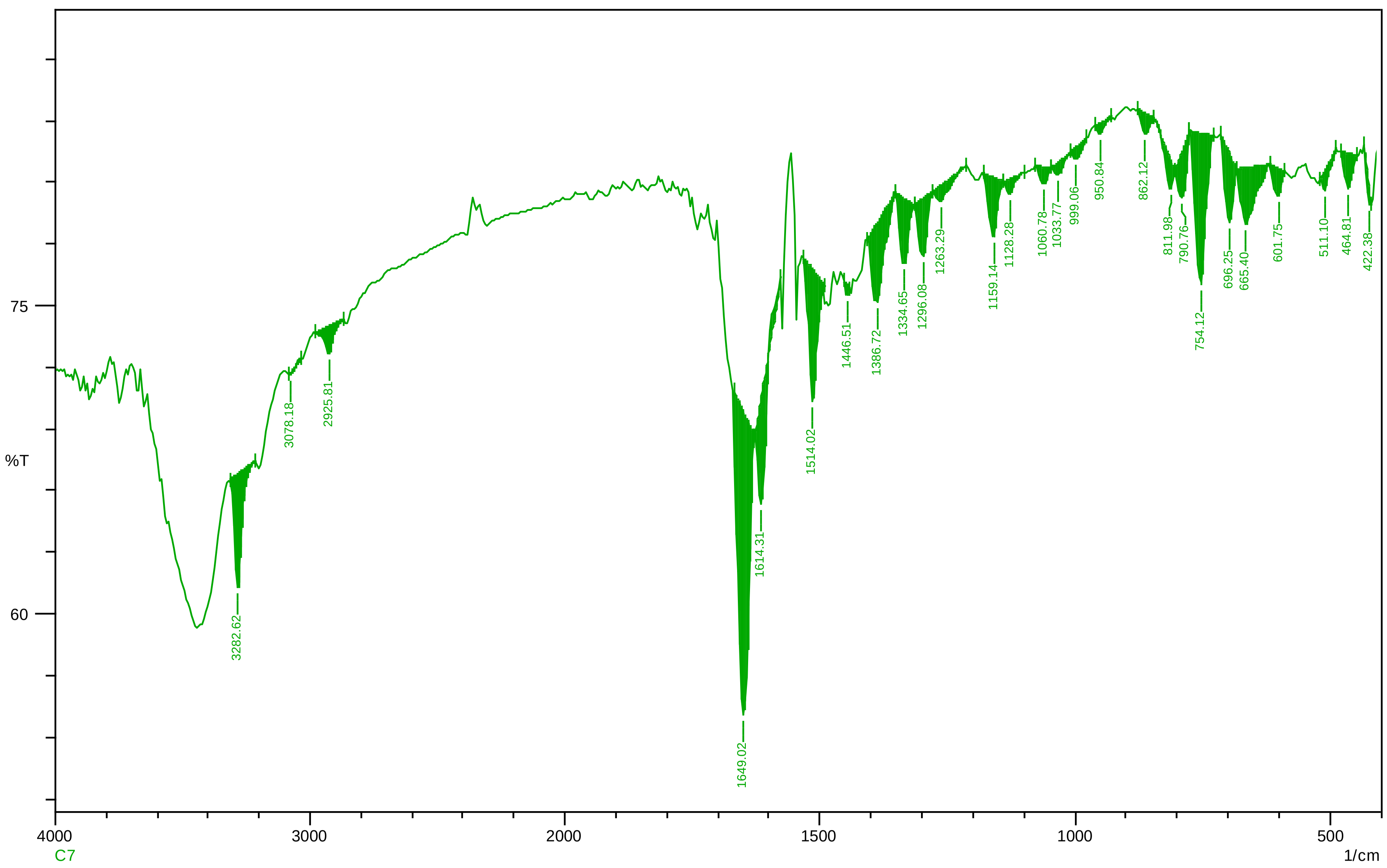

%T
75
60
4000
3000
2000
1500
1000
500
1/cm
C7
3282.62
3078.18
2925.81
1649.02
1614.31
1514.02
1446.51
1386.72
1334.65
1296.08
1263.29
1159.14
1128.28
1060.78
1033.77
999.06
950.84
862.12
811.98
790.76
754.12
696.25
665.40
601.75
511.10
464.81
422.38

PROTON DMSO {C:\Bruker\TOPSPIN\KFUCCP} nmr 16

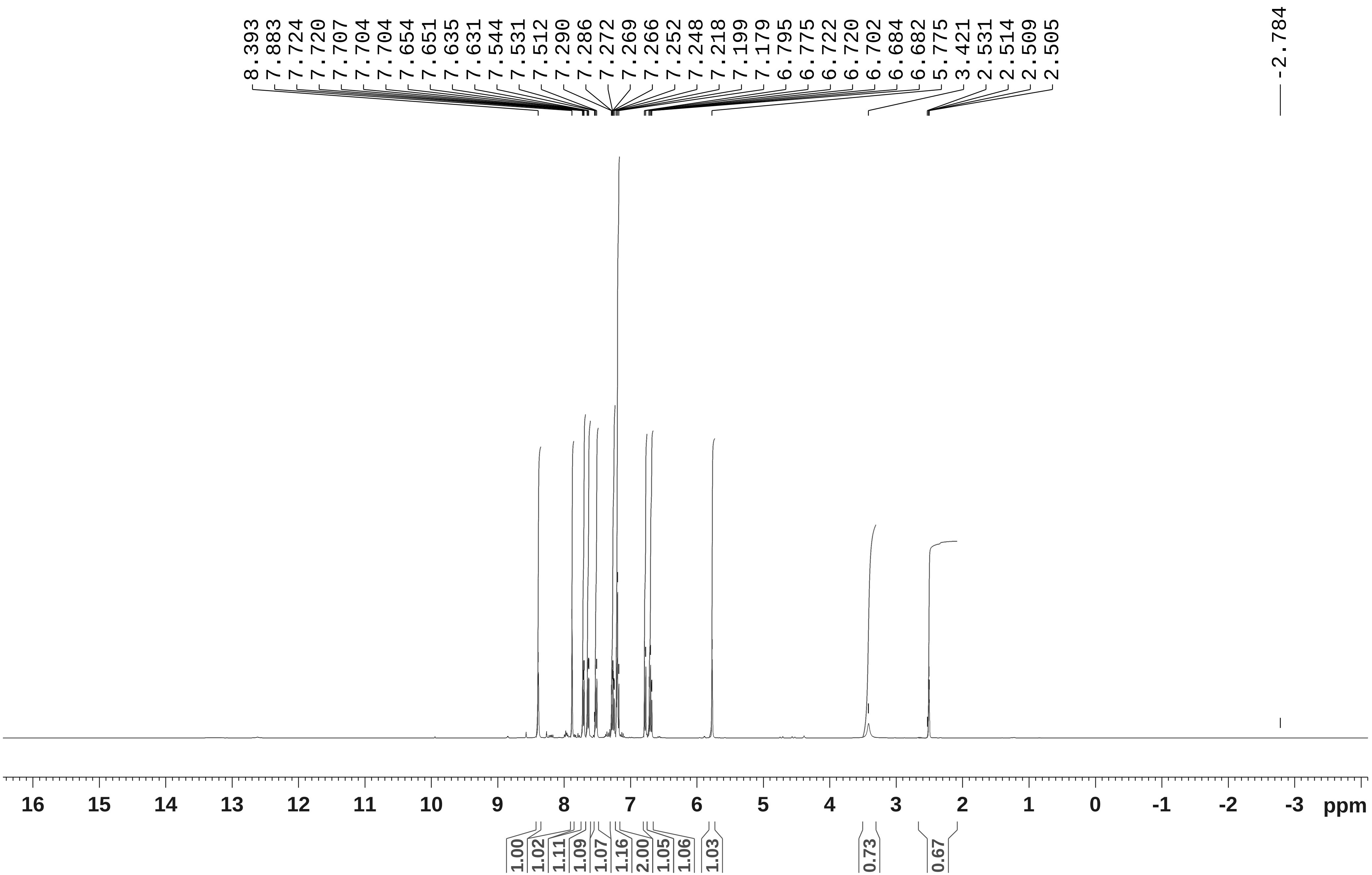

C13CPD DMSO {C:\Bruker\TOPSPIN\KFUCCP} nmr 16

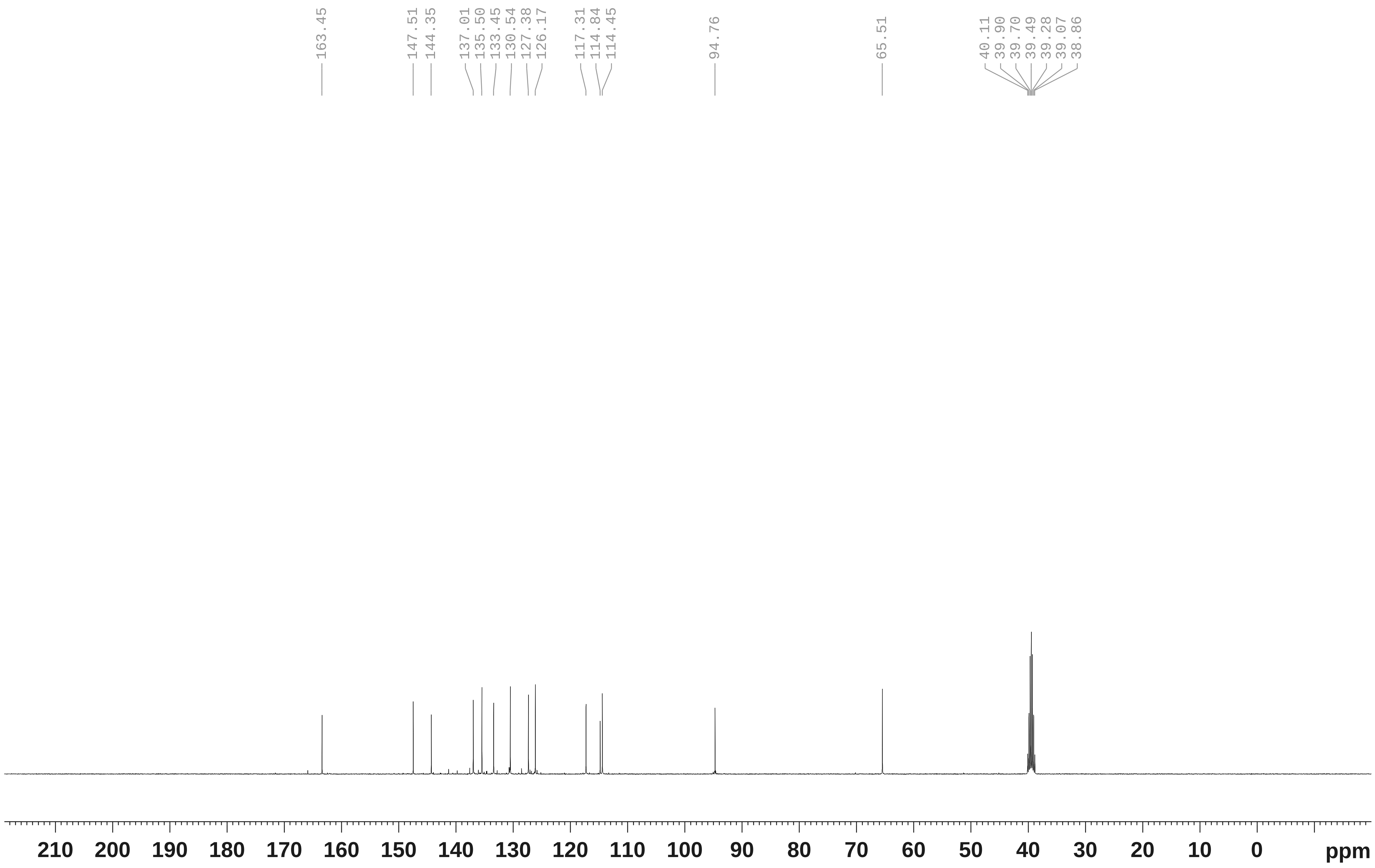

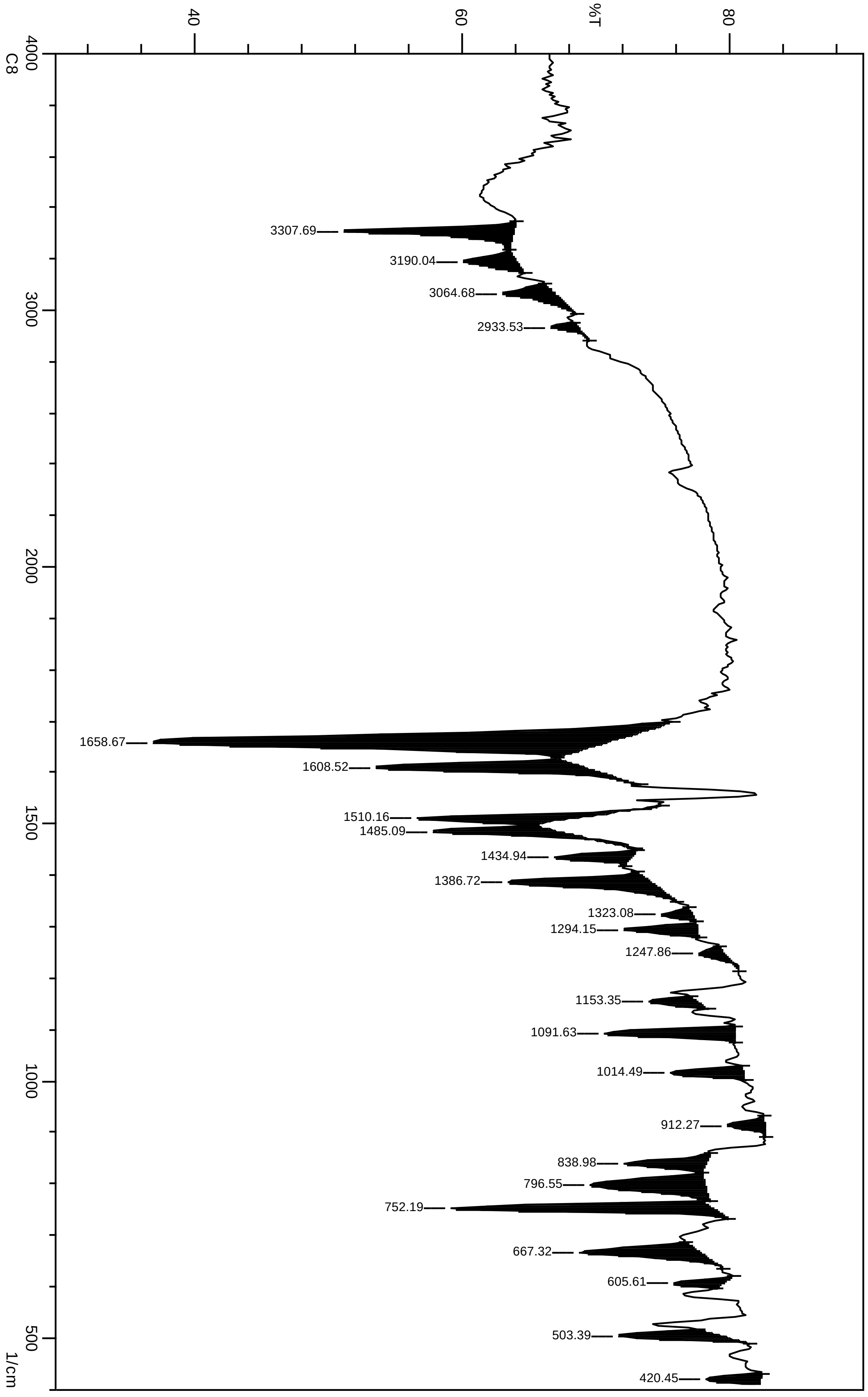
%T
40
60
80
4000
3000
2000
1500
1000
500
1/cm
C8
3307.69
3190.04
3064.68
2933.53
1658.67
1608.52
1510.16
1485.09
1434.94
1386.72
1323.08
1294.15
1247.86
1153.35
1091.63
1014.49
912.27
838.98
796.55
752.19
667.32
605.61
503.39
420.45

PROTON DMSO {C:\Bruker\TOPSPIN\KFUCCP} nmr 8

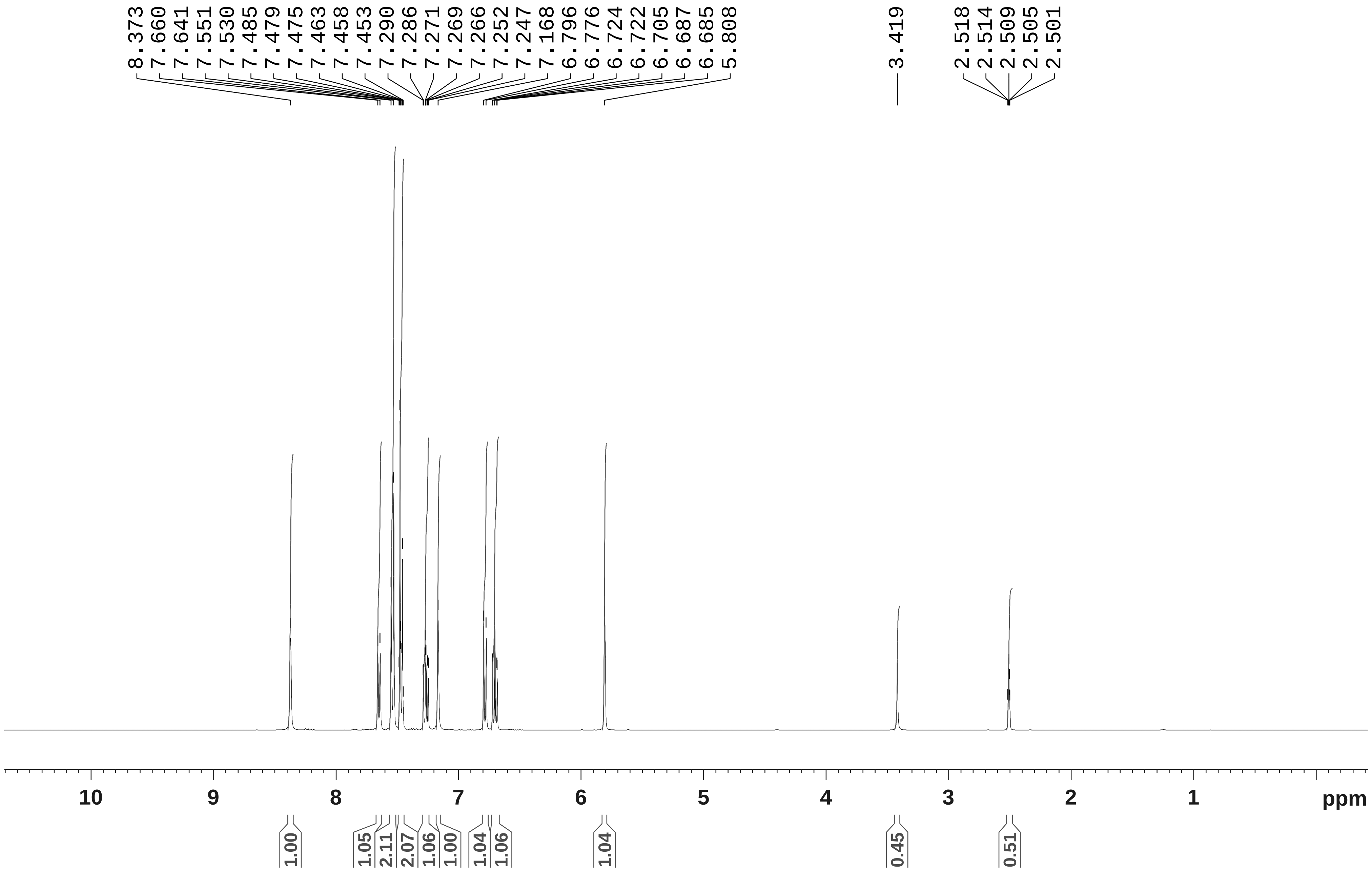

C13CPD DMSO {C:\Bruker\TOPSPIN\KFUCCP} nmr 8

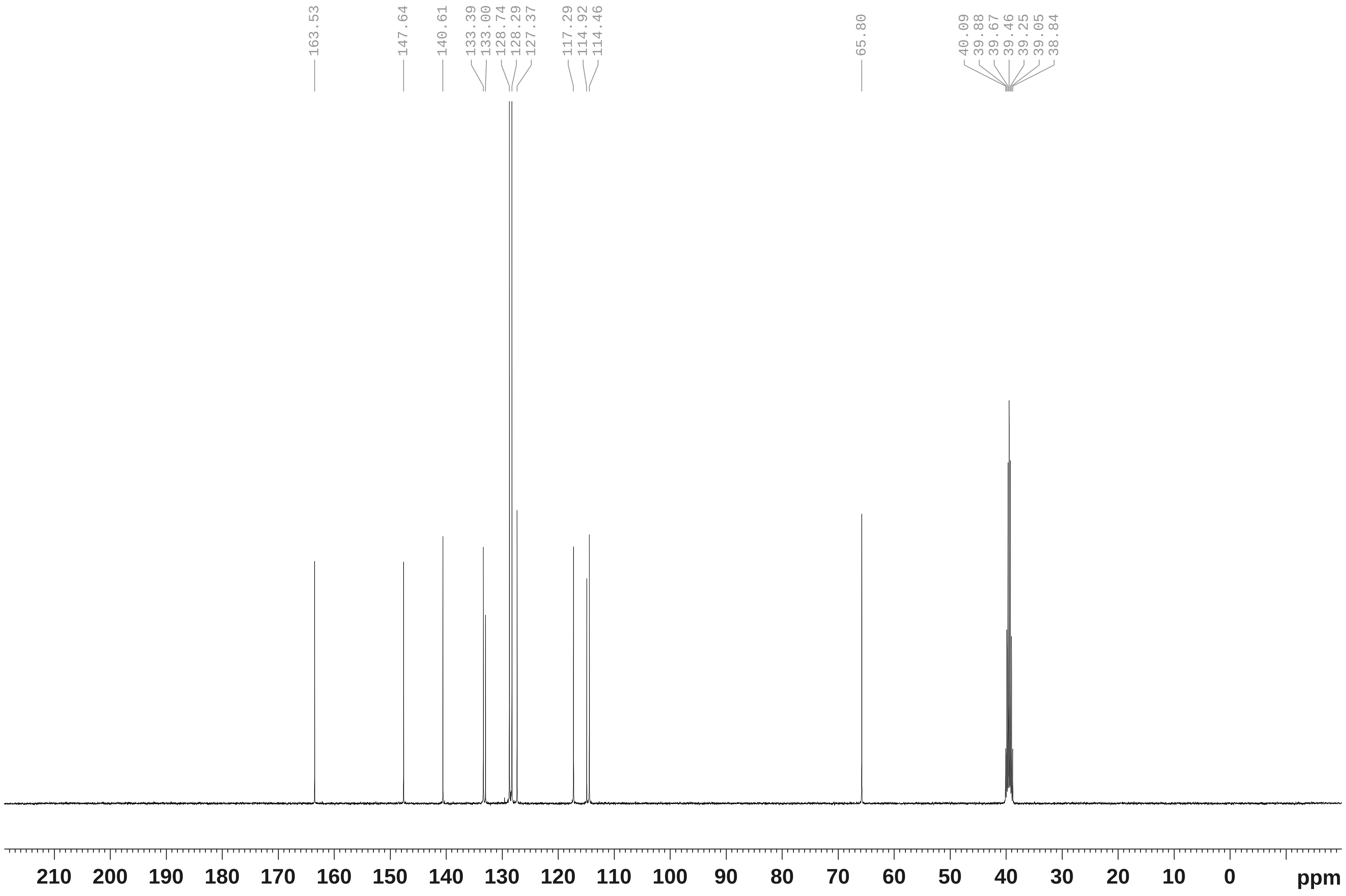

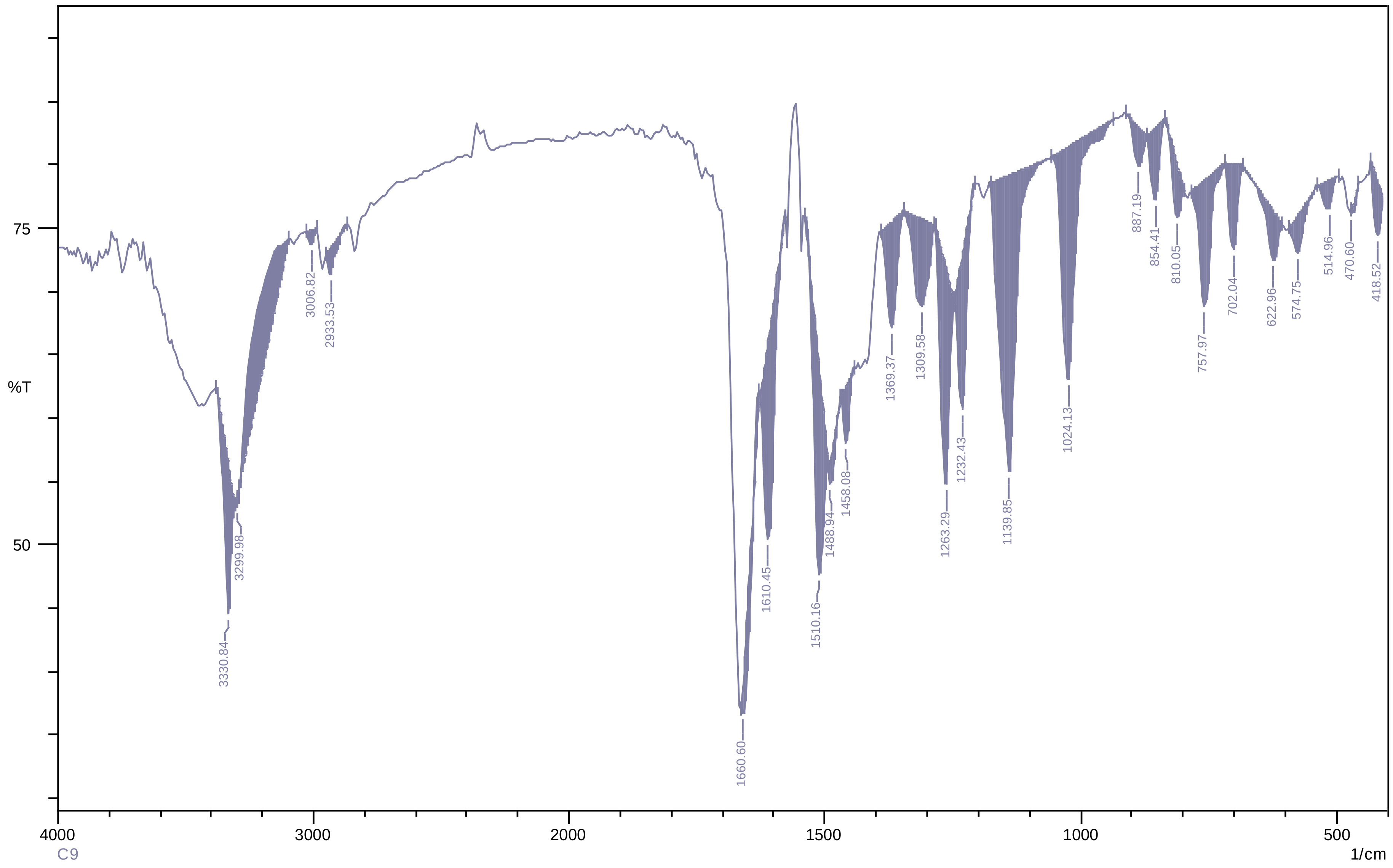

%T
75
50
3330.84
3299.98
3006.82
2933.53
1660.60
1610.45
1510.16
1488.94
1458.08
1369.37
1309.58
1263.29
1232.43
1139.85
1024.13
887.19
854.41
810.05
757.97
702.04
622.96
574.75
514.96
470.60
418.52
4000
3000
2000
1500
1000
500
1/cm
C9

PROTON DMSO {C:\Bruker\TOPSPIN\KFUCCP} nmr 7

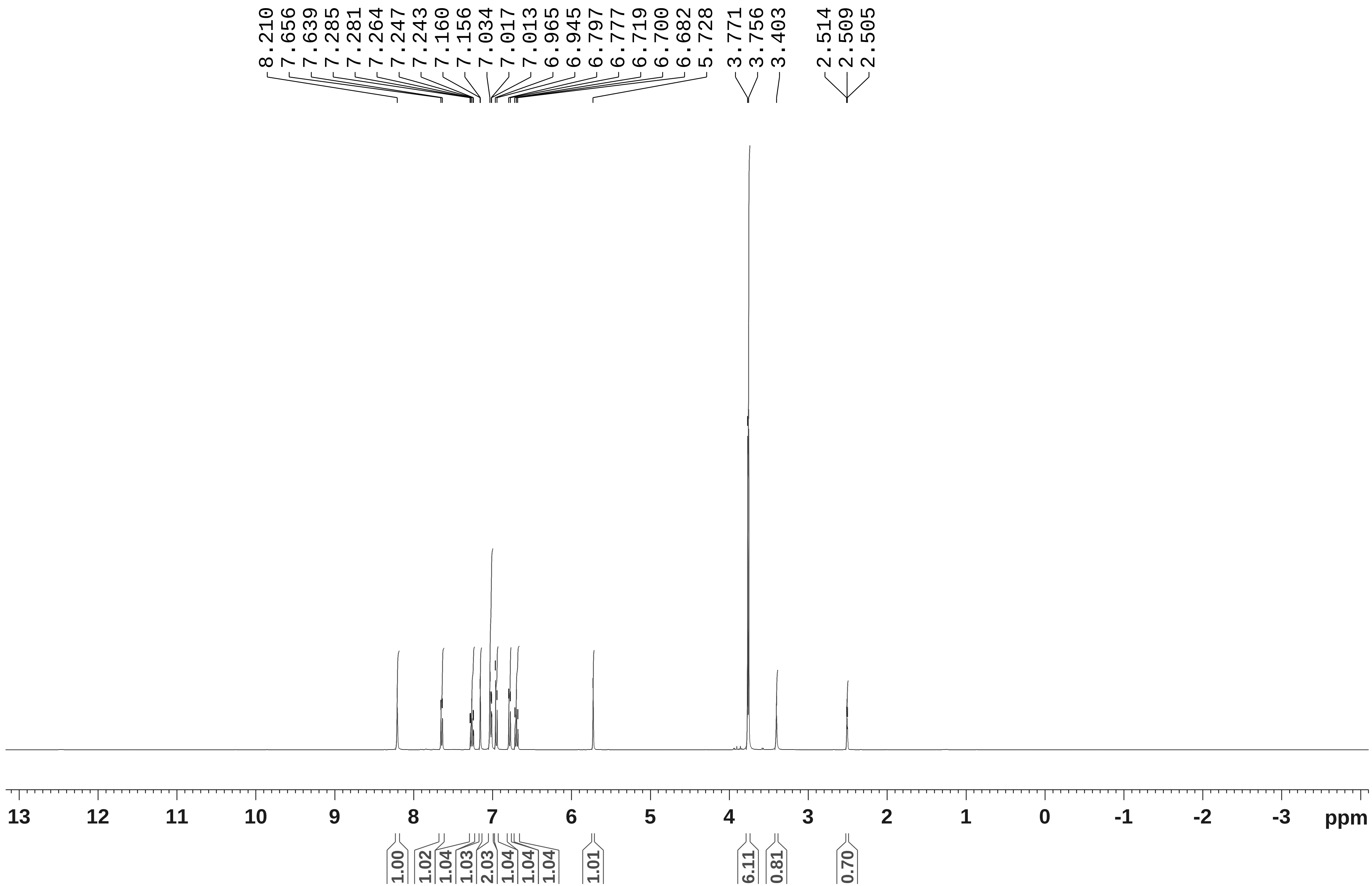

C13CPD DMSO {C:\Bruker\TOPSPIN\KFUCCP} nmr 7

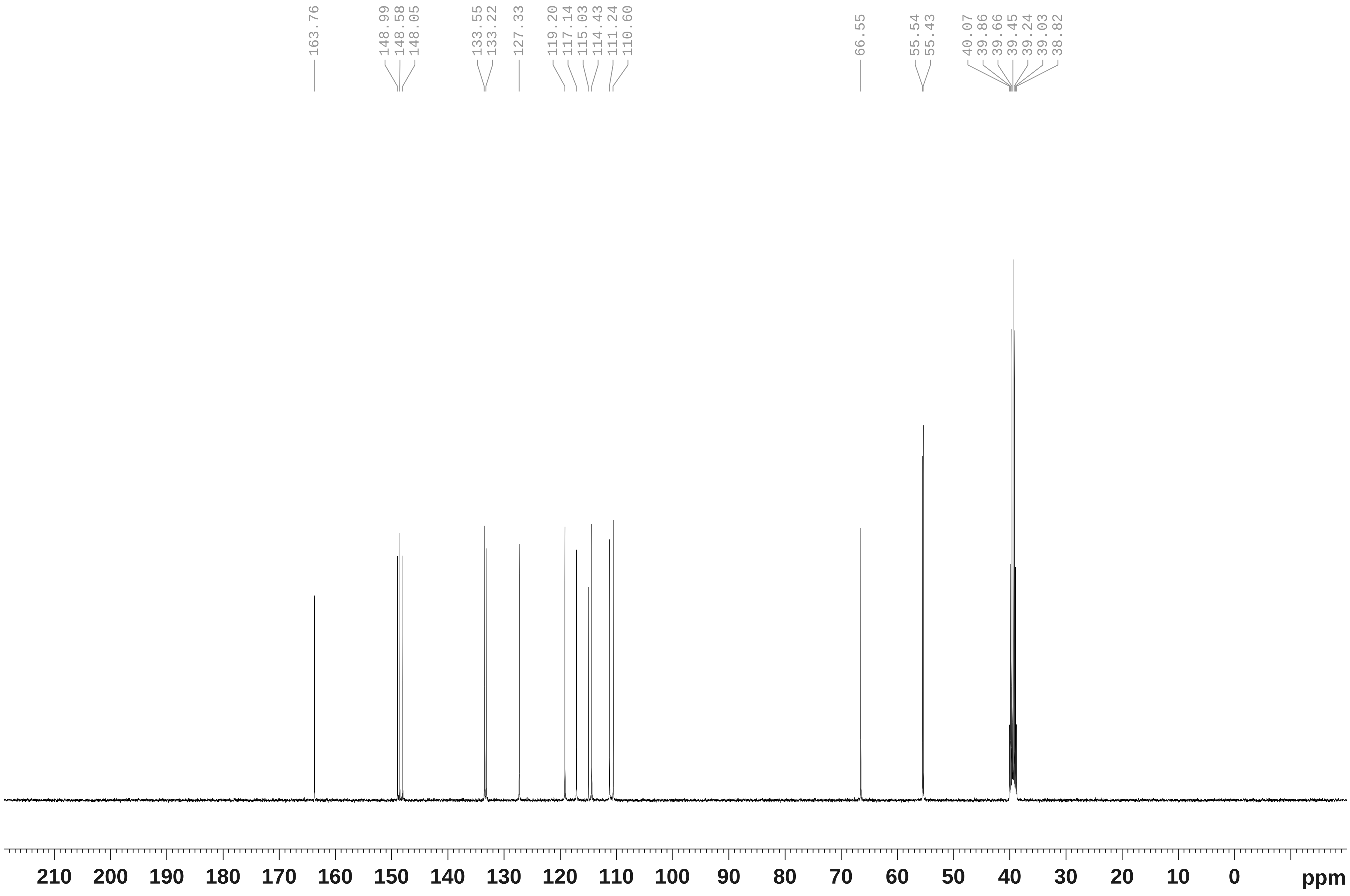

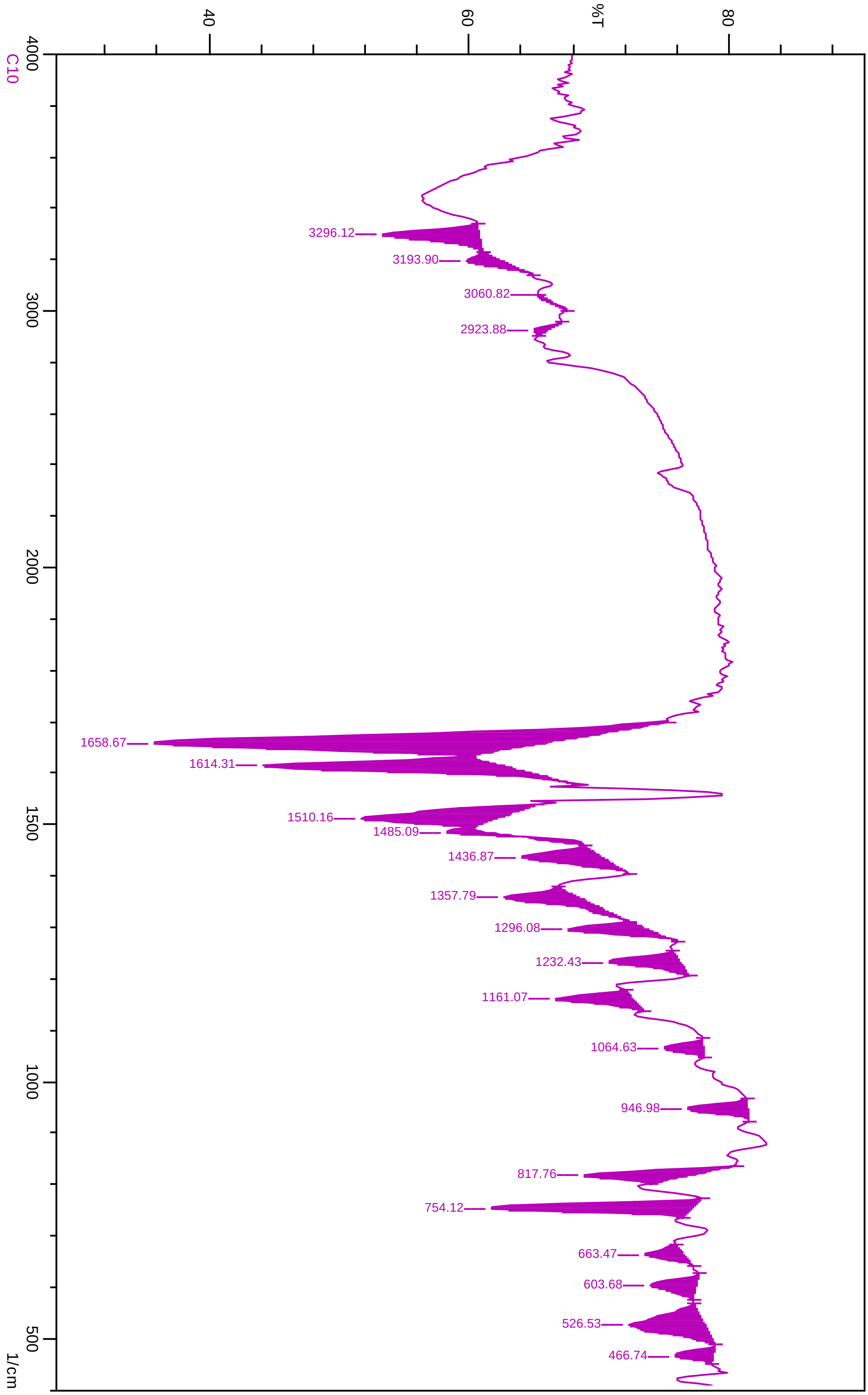

C10
%T
40
60
80
4000
3000
2000
1500
1000
500
1/cm
3296.12
3193.90
3060.82
2923.88
1658.67
1614.31
1510.16
1485.09
1436.87
1357.79
1296.08
1232.43
1161.07
1064.63
946.98
817.76
754.12
663.47
603.68
526.53
466.74

PROTON DMSO {C:\Bruker\TOPSPIN\KFUCCP} nmr 14

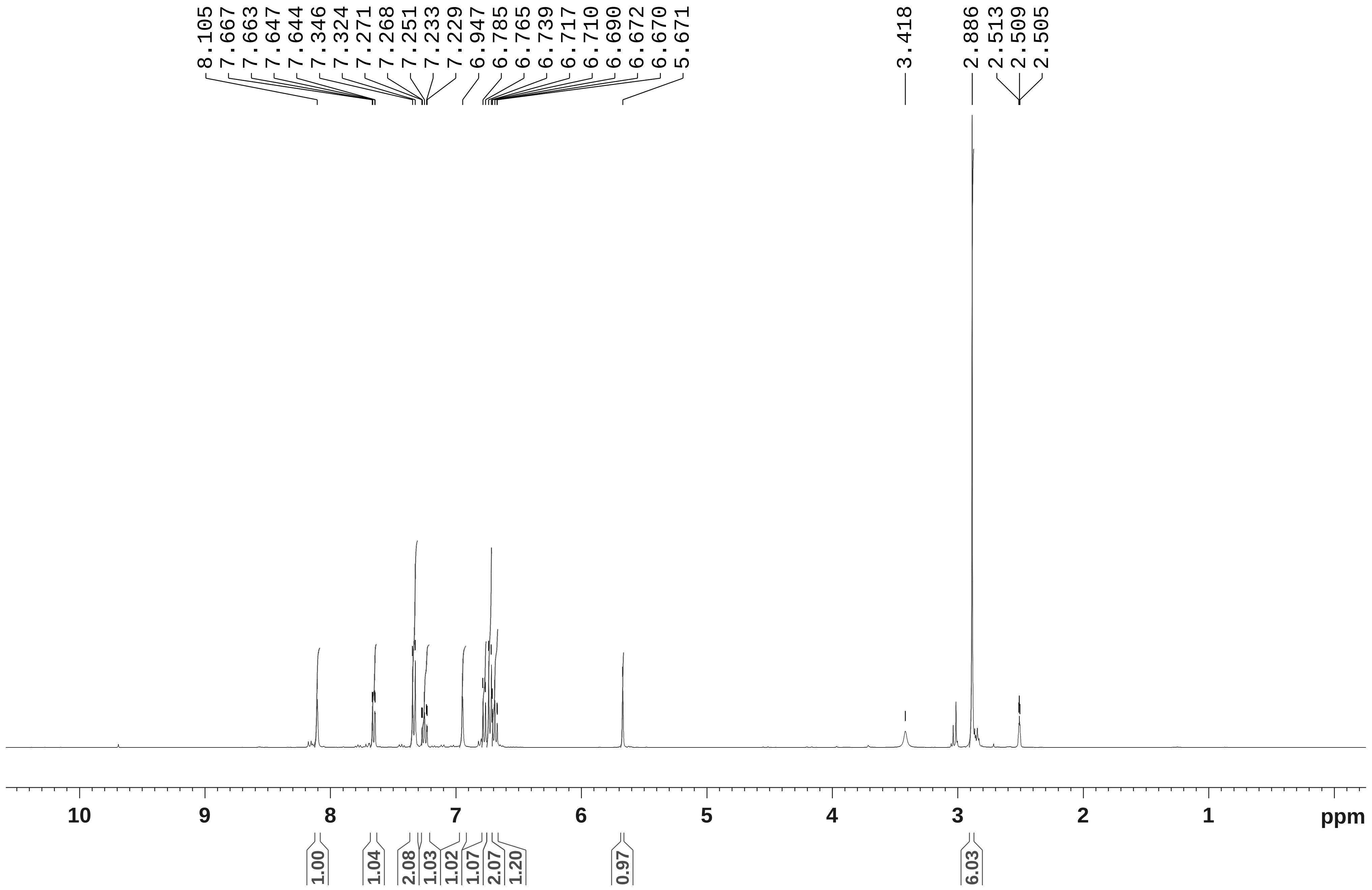

C13CPD DMSO {C:\Bruker\TOPSPIN\KFUCCP} nmr 14

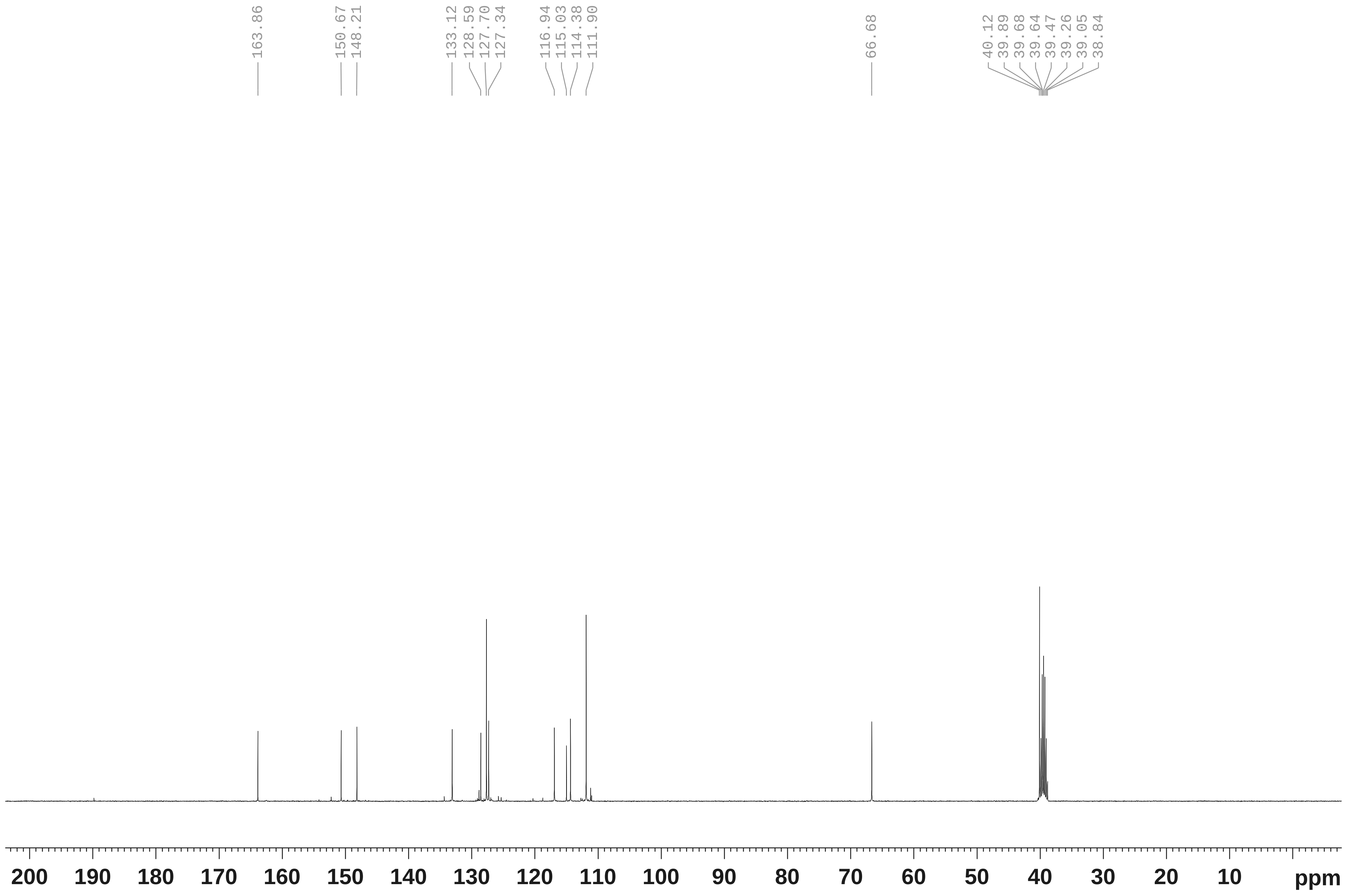

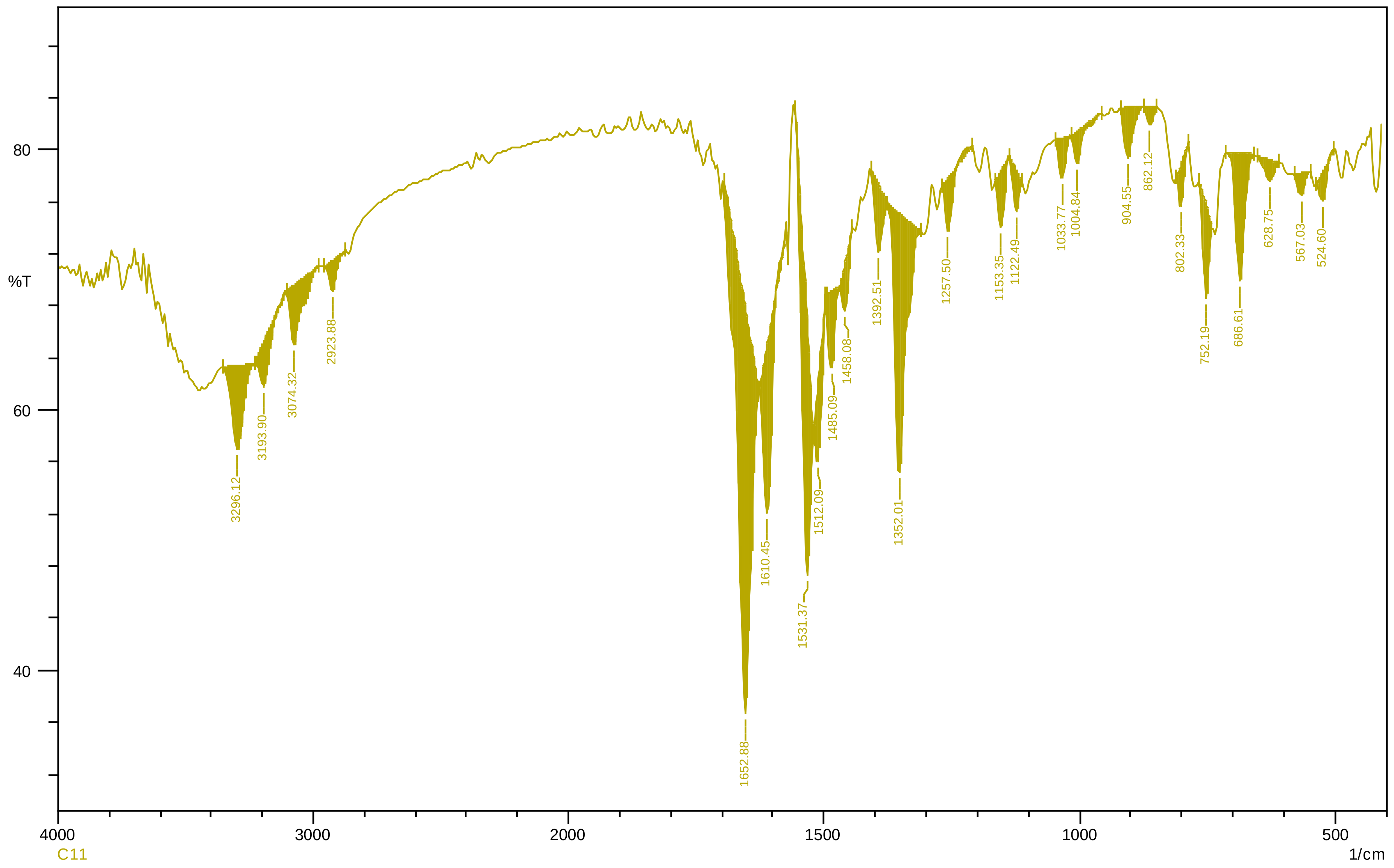
%T
80
60
40
4000
3000
2000
1500
1000
500
1/cm
C11
3296.12
3193.90
3074.32
2923.88
1652.88
1610.45
1531.37
1512.09
1485.09
1458.08
1392.51
1352.01
1257.50
1153.35
1122.49
1033.77
1004.84
904.55
862.12
802.33
752.19
686.61
628.75
567.03
524.60

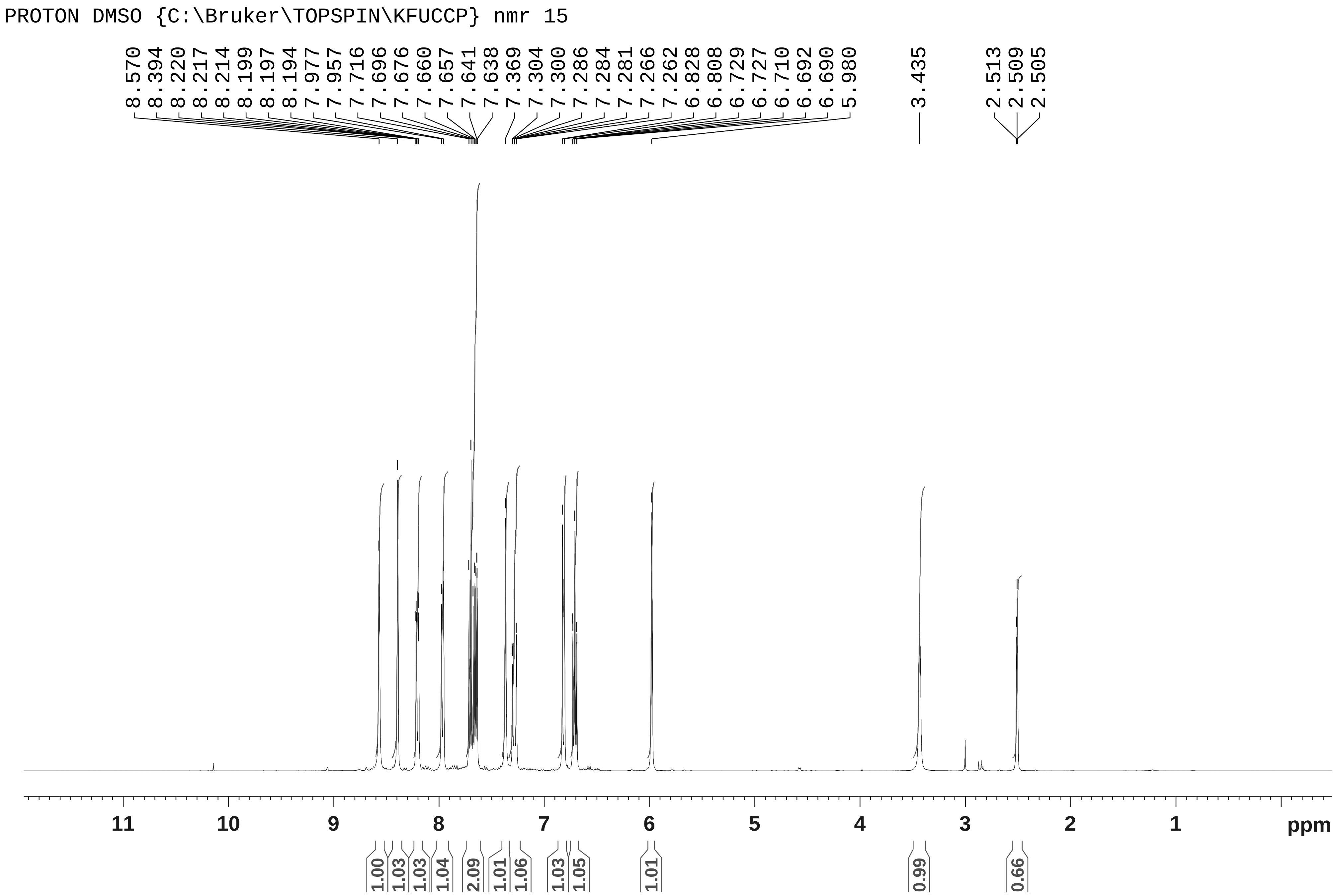

PROTON DMSO {C:\Bruker\TOPSPIN\KFUCCP} nmr 15
8.570
8.394
8.220
8.217
8.214
8.199
8.197
8.194
7.977
7.957
7.716
7.696
7.676
7.660
7.657
7.641
7.638
7.369
7.304
7.300
7.286
7.284
7.281
7.266
7.262
6.828
6.808
6.729
6.727
6.710
6.692
6.690
5.980
3.435
2.513
2.509
2.505
1.00
1.03
1.03
1.04
2.09
1.01
1.06
1.03
1.05
1.01
0.99
0.66
11
10
9
8
7
6
5
4
3
2
1
ppm

C13CPD DMSO {C:\Bruker\TOPSPIN\KFUCCP} nmr 15

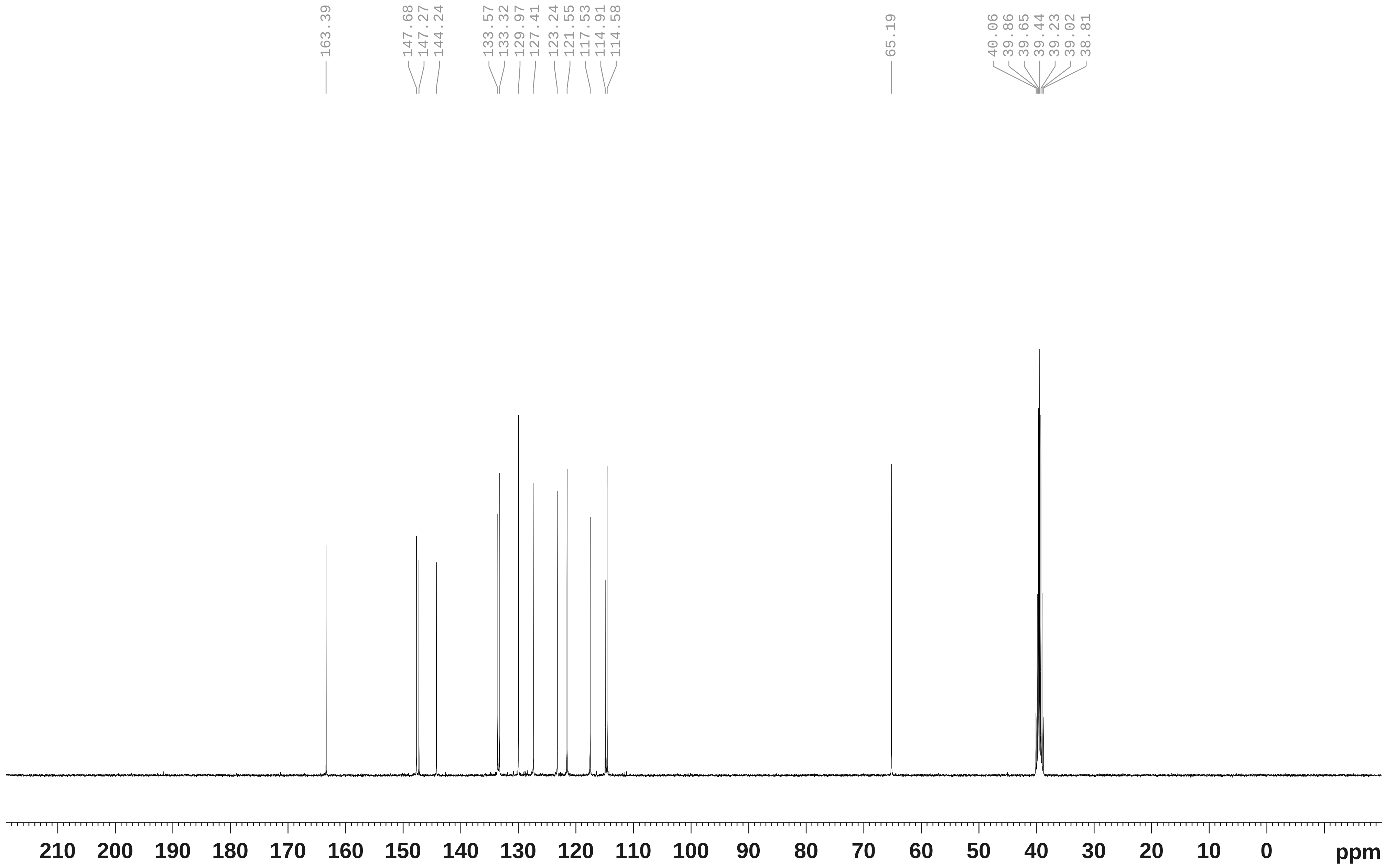

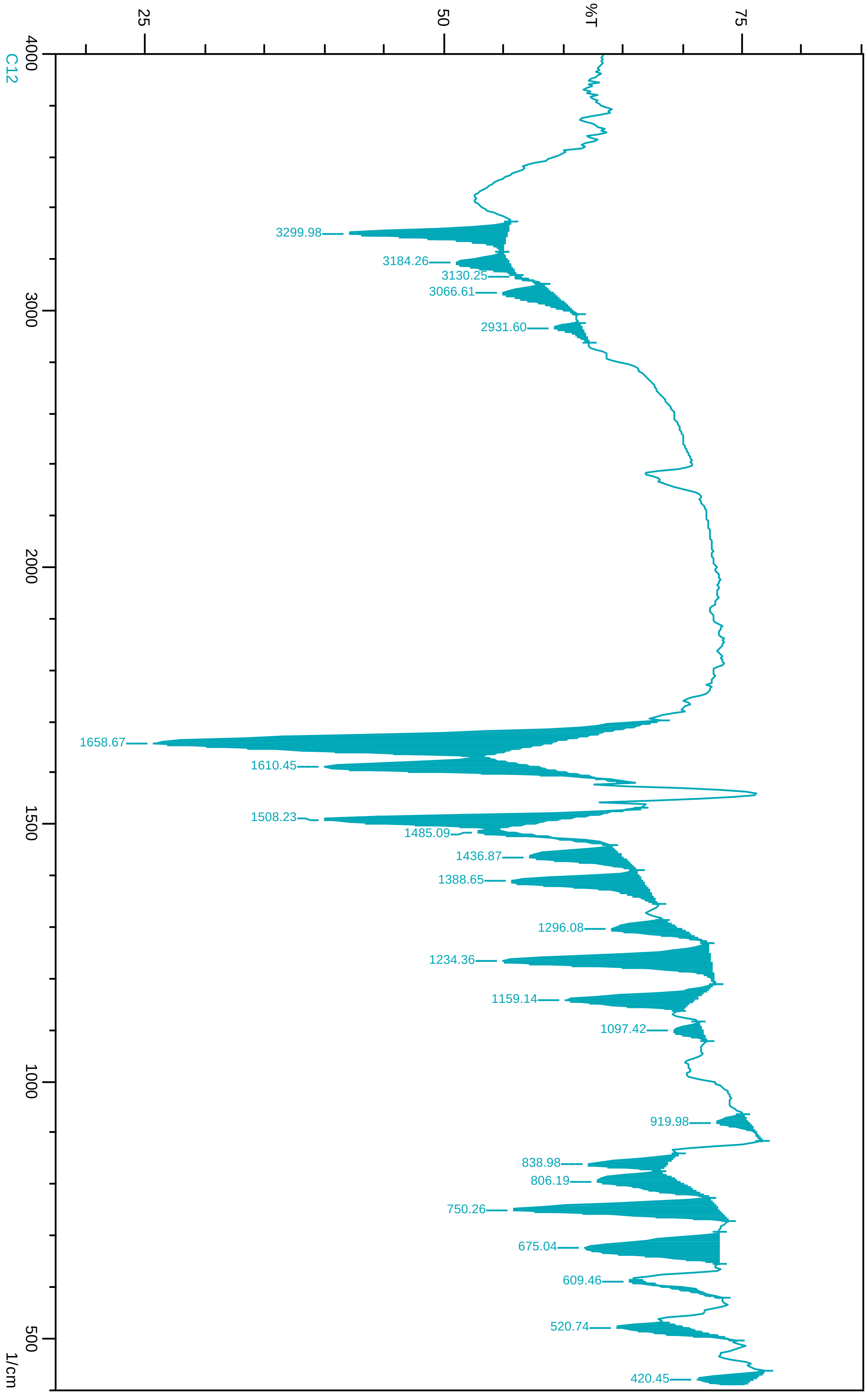
C12
%T
25
50
75
4000
3000
2000
1500
1000
500
1/cm
3299.98
3184.26
3130.25
3066.61
2931.60
1658.67
1610.45
1508.23
1485.09
1436.87
1388.65
1296.08
1234.36
1159.14
1097.42
919.98
838.98
806.19
750.26
675.04
609.46
520.74
420.45

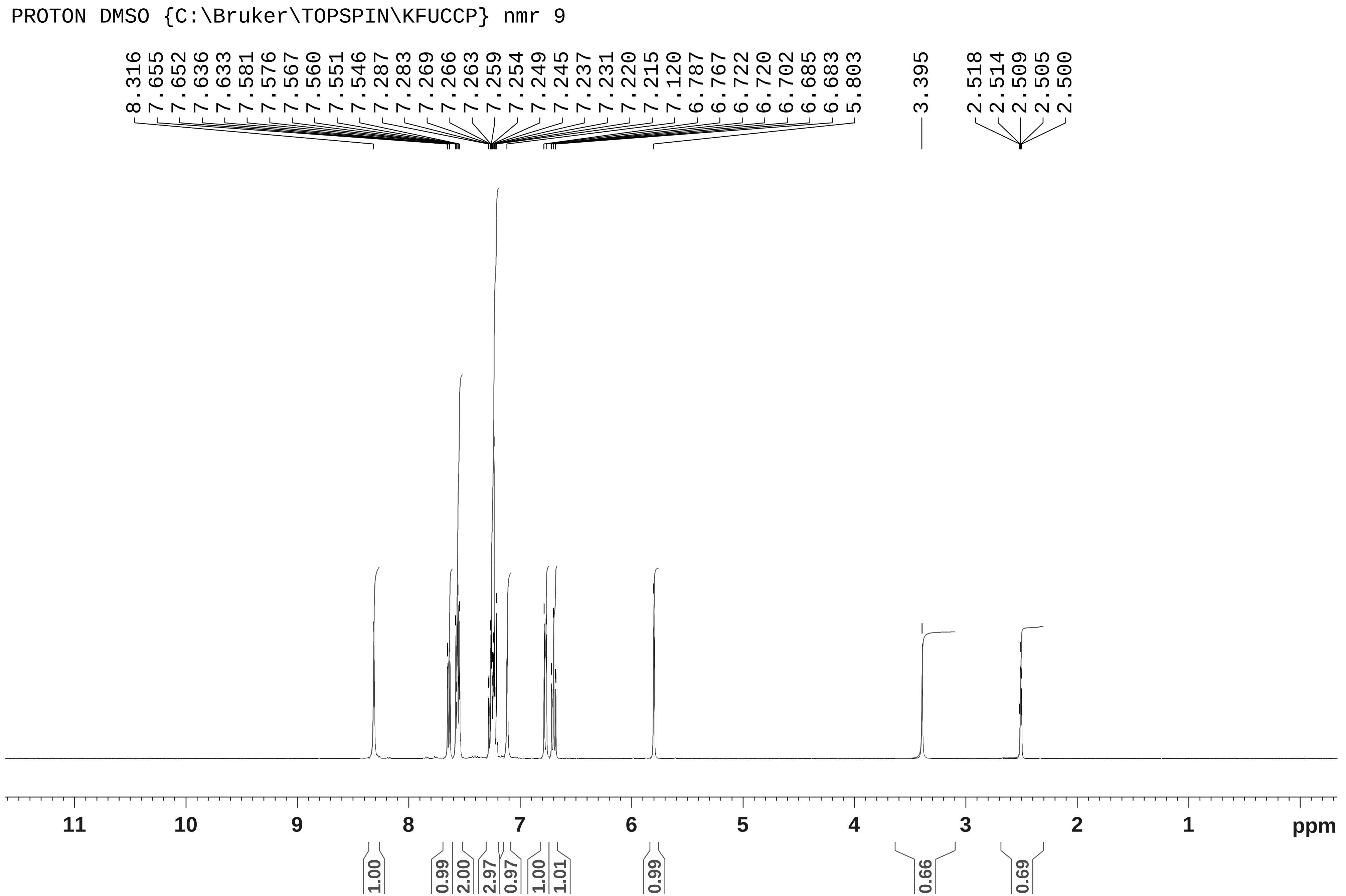
PROTON DMSO {C:\Bruker\TOPSPIN\KFUCCP} nmr 9
8.316
7.655
7.652
7.636
7.633
7.581
7.576
7.567
7.560
7.551
7.546
7.287
7.283
7.269
7.266
7.263
7.259
7.254
7.249
7.245
7.237
7.231
7.220
7.215
7.120
6.787
6.767
6.722
6.720
6.702
6.685
6.683
5.803
3.395
2.518
2.514
2.509
2.505
2.500
11
10
9
8
7
6
5
4
3
2
1
ppm
1.00
0.99
2.00
2.97
0.97
1.00
1.01
0.99
0.66
0.69

C13CPD DMSO {C:\Bruker\TOPSPIN\KFUCCP} nmr 9

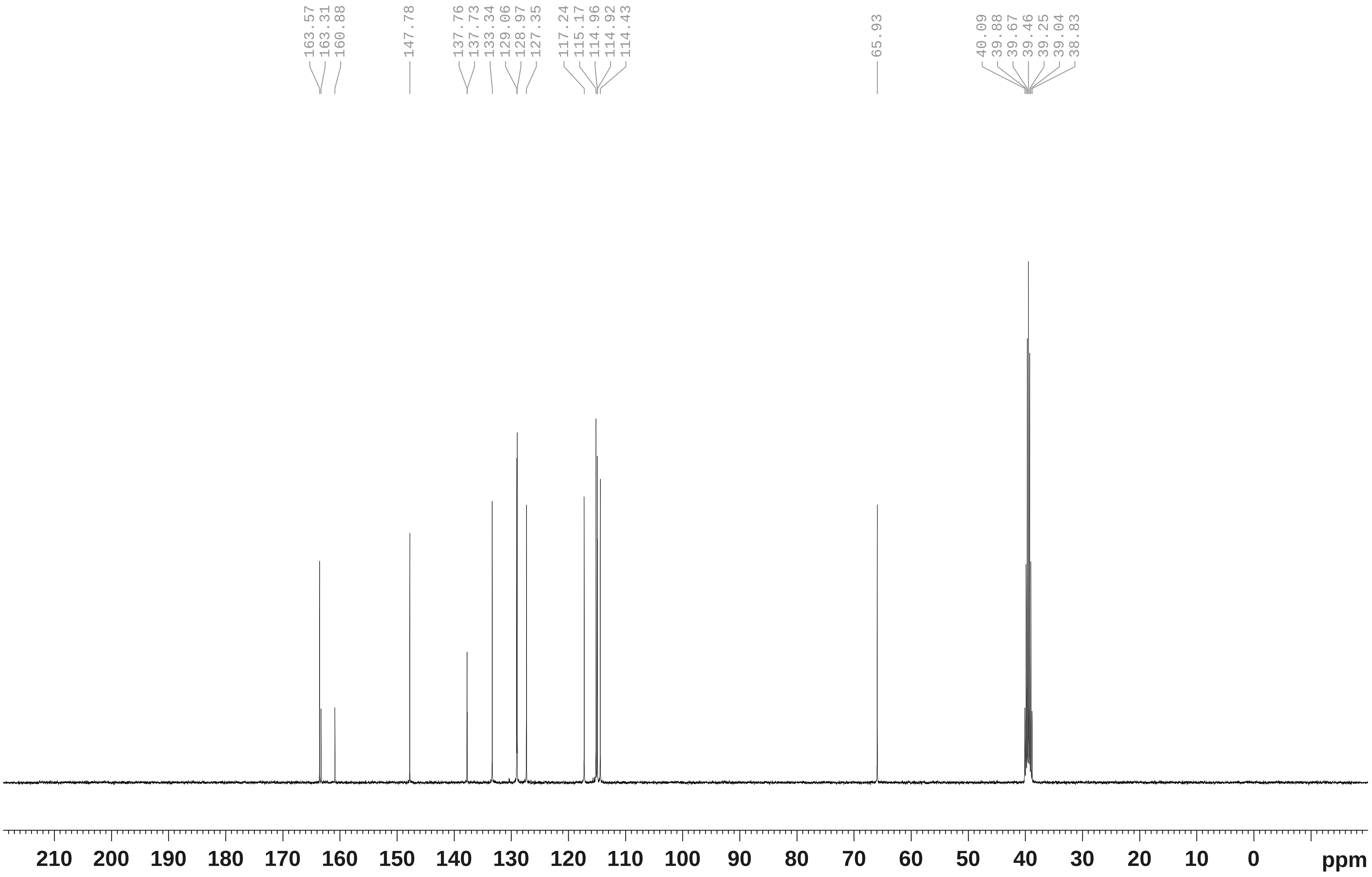

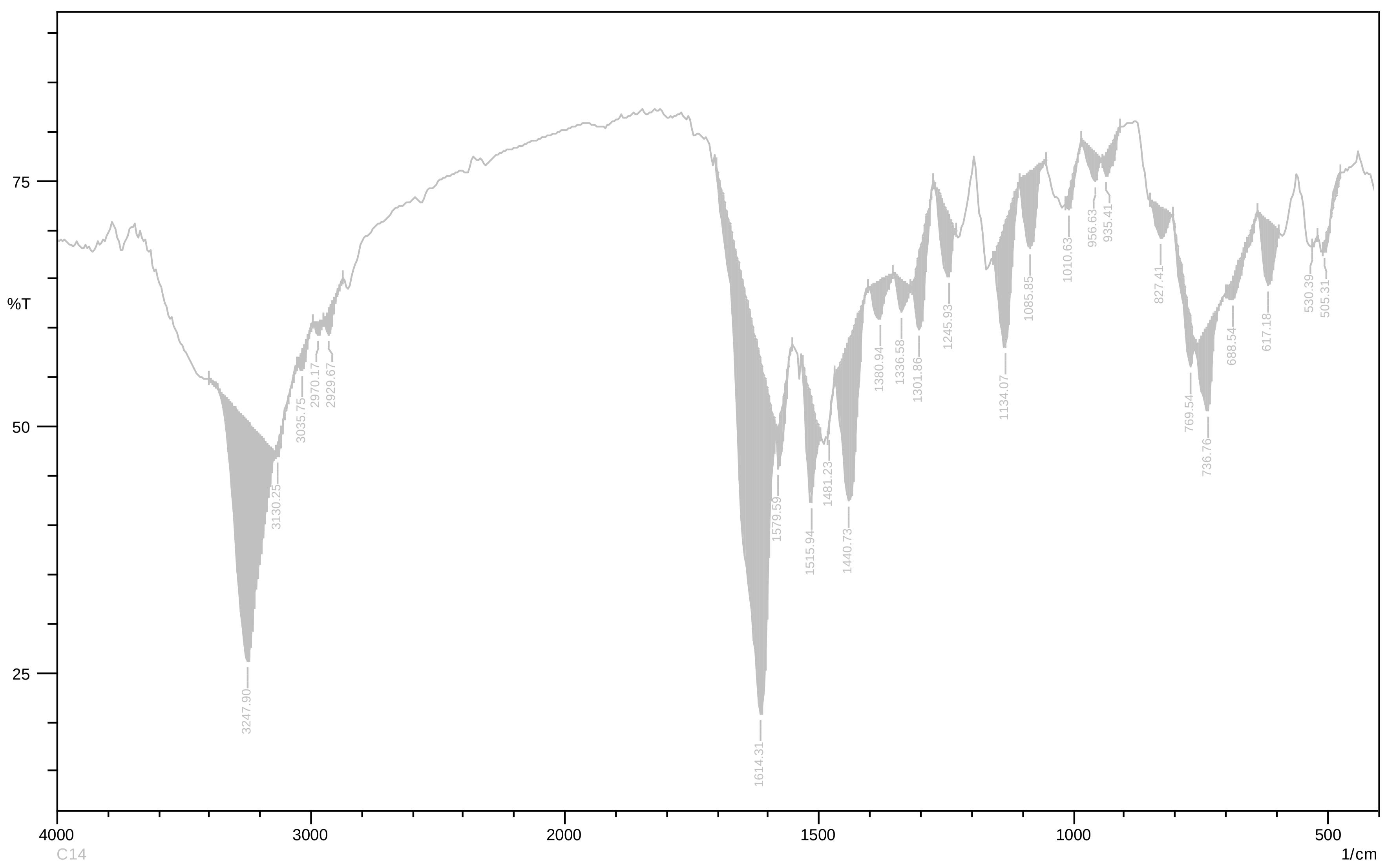
%T
75
50
25
3247.90
3130.25
3035.75
2970.17
2929.67
1614.31
1579.59
1515.94
1481.23
1440.73
1380.94
1336.58
1301.86
1245.93
1134.07
1085.85
1010.63
956.63
935.41
827.41
769.54
736.76
688.54
617.18
530.39
505.31
4000
3000
2000
1500
1000
500
1/cm
C14

PROTON DMSO {C:\Bruker\TOPSPIN\KFUCCP} nmr 3

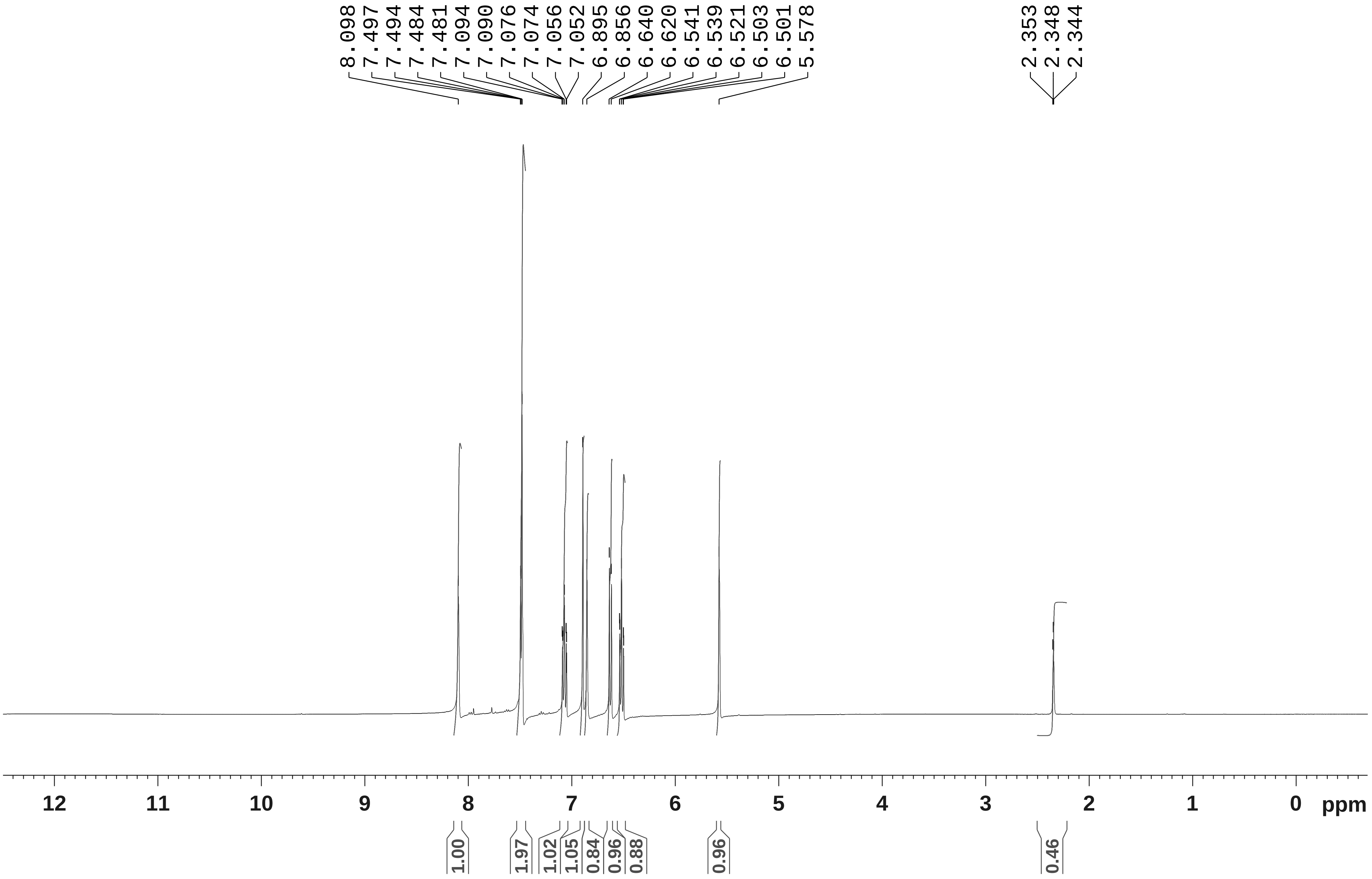

C13CPD DMSO {C:\Bruker\TOPSPIN\KFUCCP} nmr 3

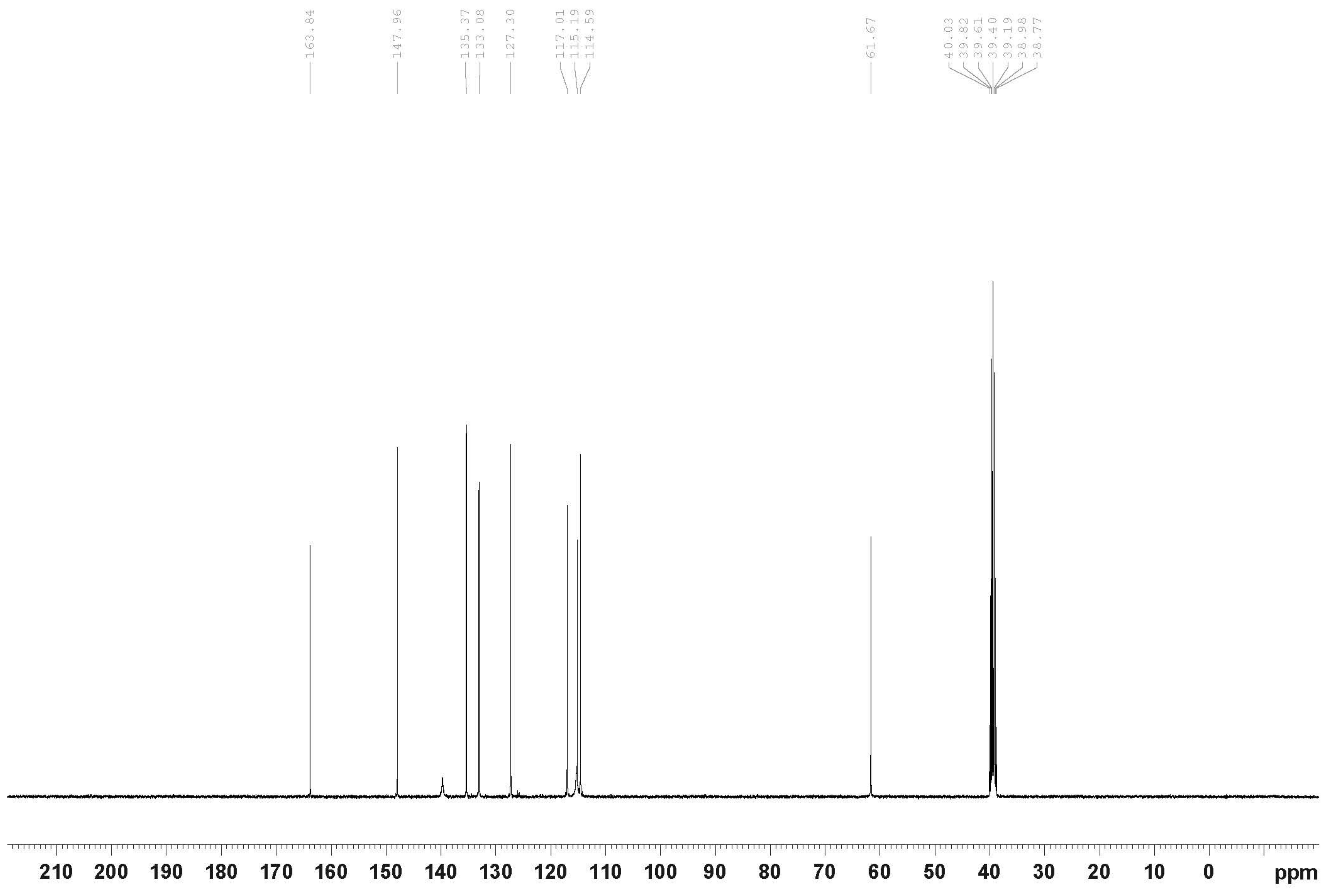

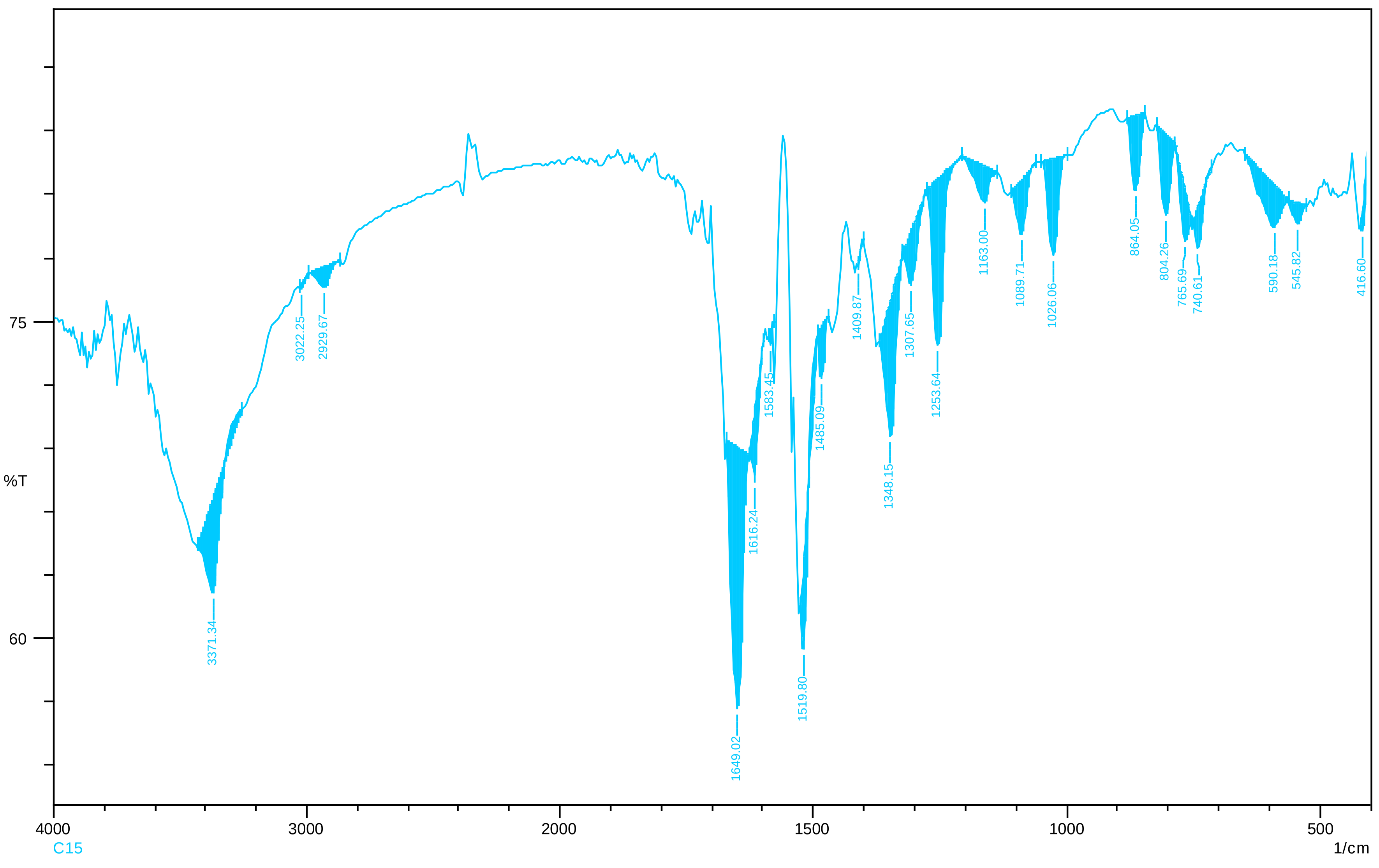

%T
75
60
4000
3000
2000
1500
1000
500
1/cm
C15
3371.34
3022.25
2929.67
1649.02
1616.24
1583.45
1519.80
1485.09
1409.87
1348.15
1307.65
1253.64
1163.00
1089.71
1026.06
864.05
804.26
765.69
740.61
590.18
545.82
416.60

PROTON DMSO {C:\Bruker\TOPSPIN\KFUCCP} nmr 4

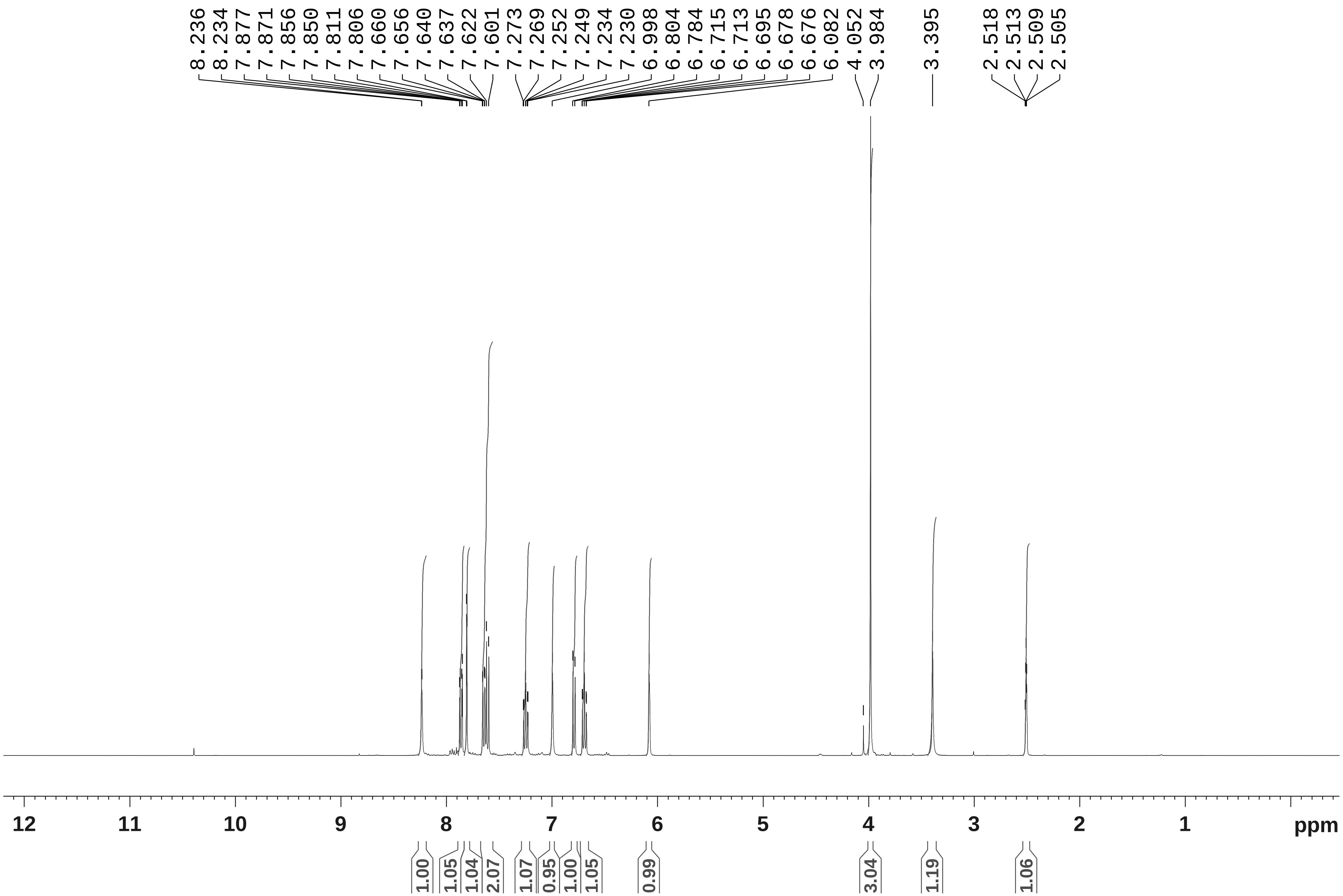

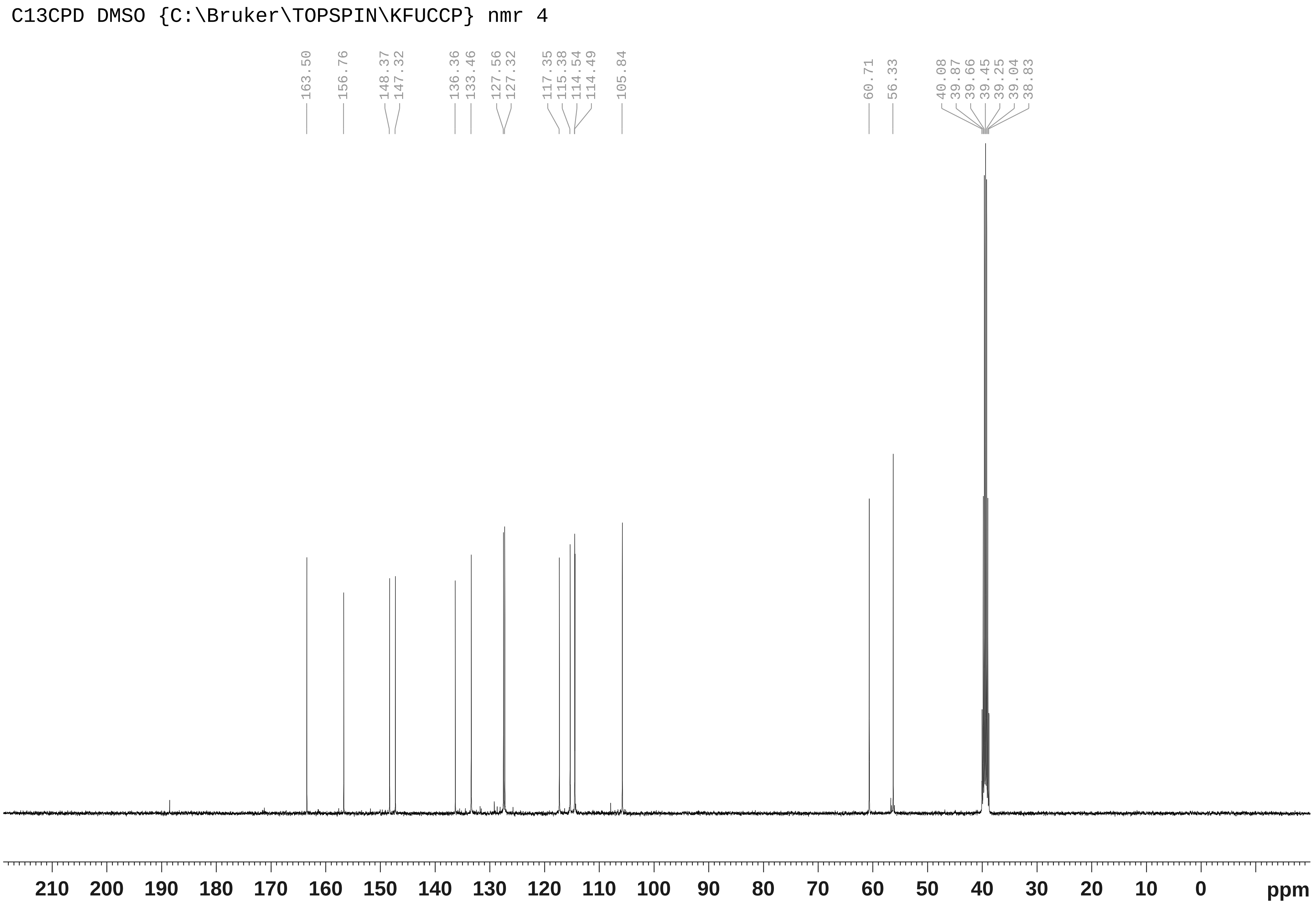
C13CPD DMSO {C:\Bruker\TOPSPIN\KFUCCP} nmr 4
163.50
156.76
148.37
147.32
136.36
133.46
127.56
127.32
117.35
115.38
114.54
114.49
105.84
60.71
56.33
40.08
39.87
39.66
39.45
39.25
39.04
38.83
210 200 190 180 170 160 150 140 130 120 110 100 90 80 70 60 50 40 30 20 10 0 ppm

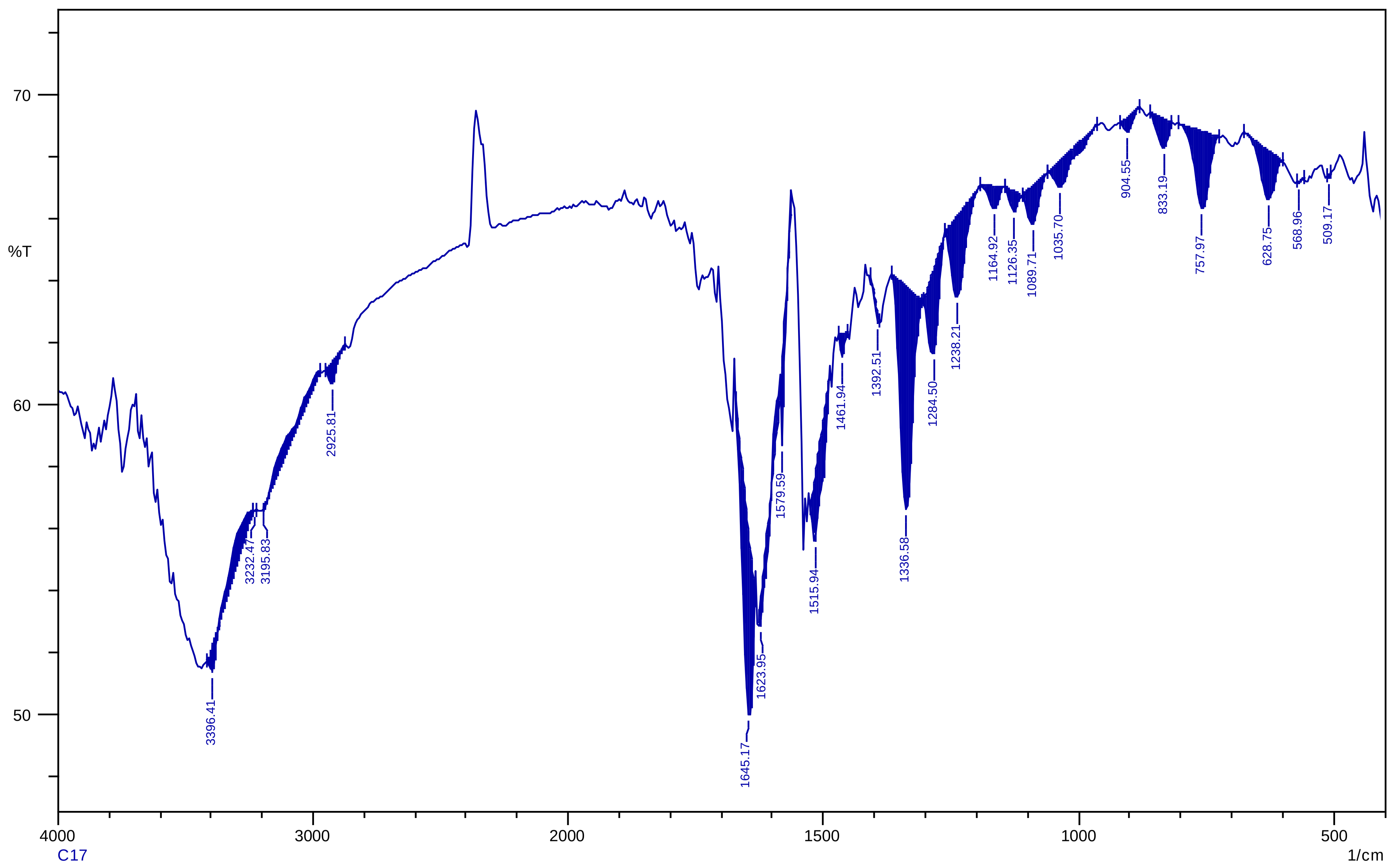

70
60
50
%T
4000
3000
2000
1500
1000
500
1/cm
C17
3396.41
3232.47
3195.83
2925.81
1645.17
1623.95
1579.59
1515.94
1461.94
1392.51
1336.58
1284.50
1238.21
1164.92
1126.35
1089.71
1035.70
904.55
833.19
757.97
628.75
568.96
509.17

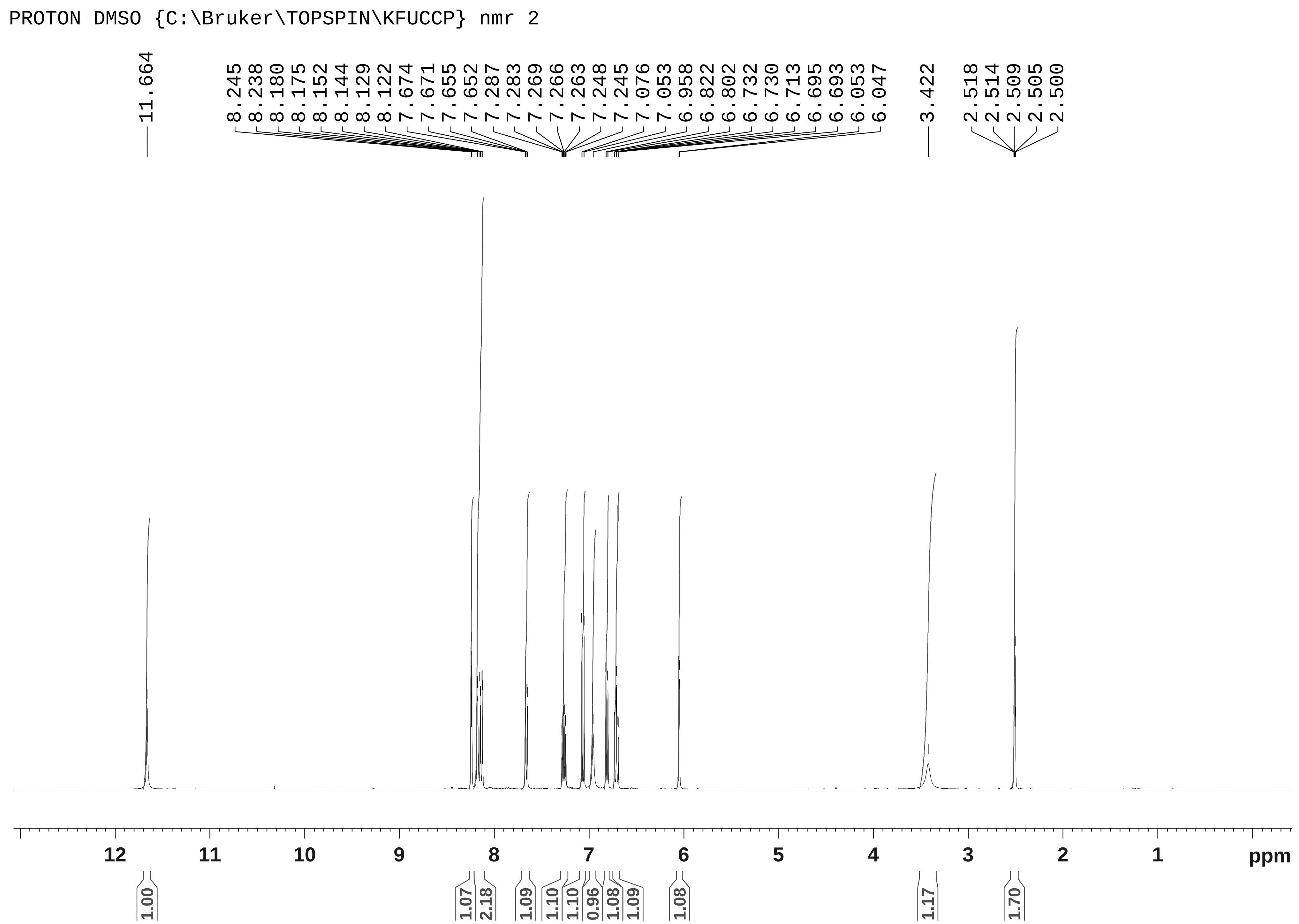
PROTON DMSO {C:\Bruker\TOPSPIN\KFUCCP} nmr 2
11.664
8.245
8.238
8.180
8.175
8.152
8.144
8.129
8.122
7.674
7.671
7.655
7.652
7.287
7.283
7.269
7.266
7.263
7.248
7.245
7.076
7.053
6.958
6.822
6.802
6.732
6.730
6.713
6.695
6.693
6.053
6.047
3.422
2.518
2.514
2.509
2.505
2.500
12
11
10
9
8
7
6
5
4
3
2
1
ppm
1.00
1.07
2.18
1.09
1.10
1.10
0.96
1.08
1.09
1.08
1.17
1.70

C13CPD DMSO {C:\Bruker\TOPSPIN\KFUCCP} nmr 2

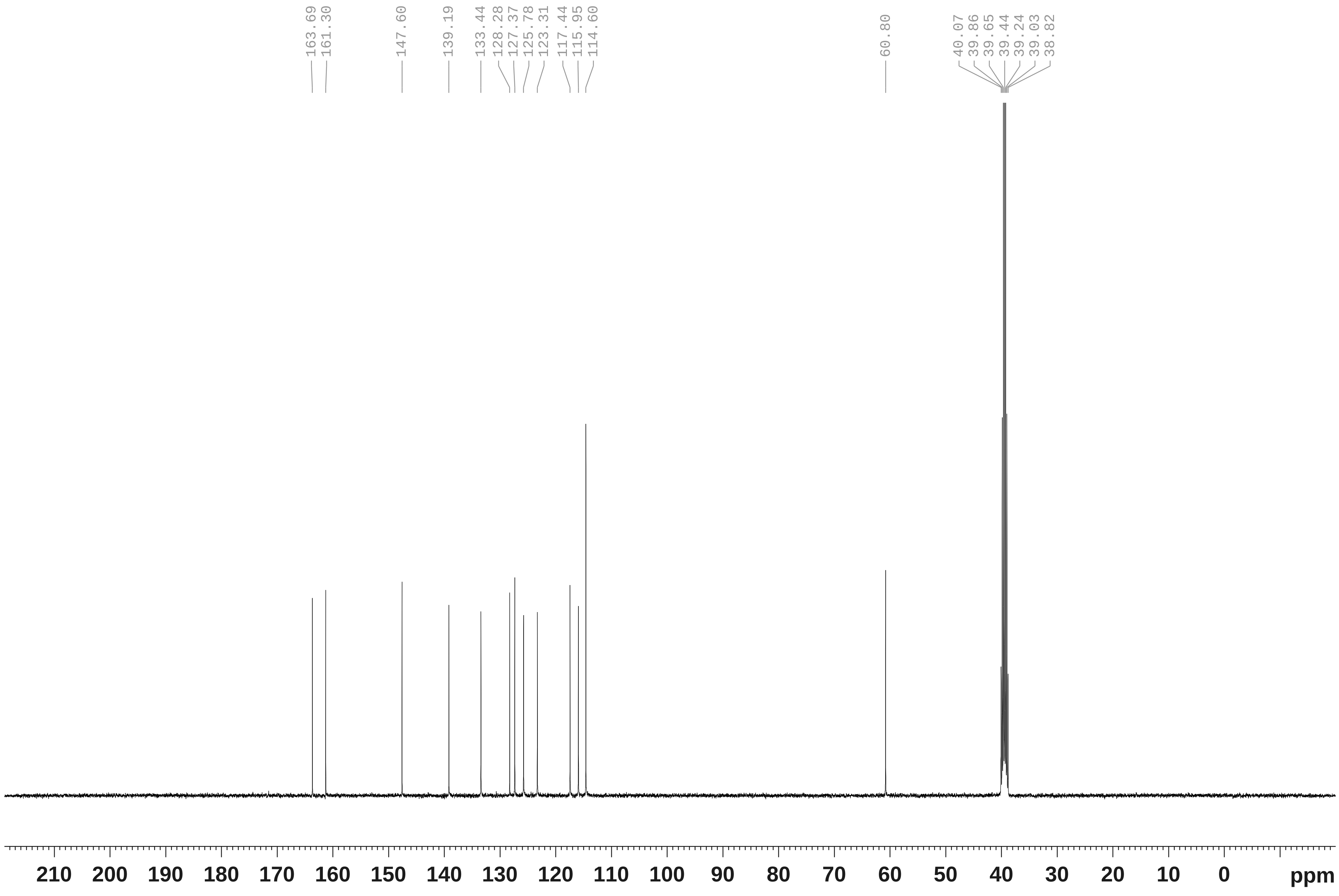

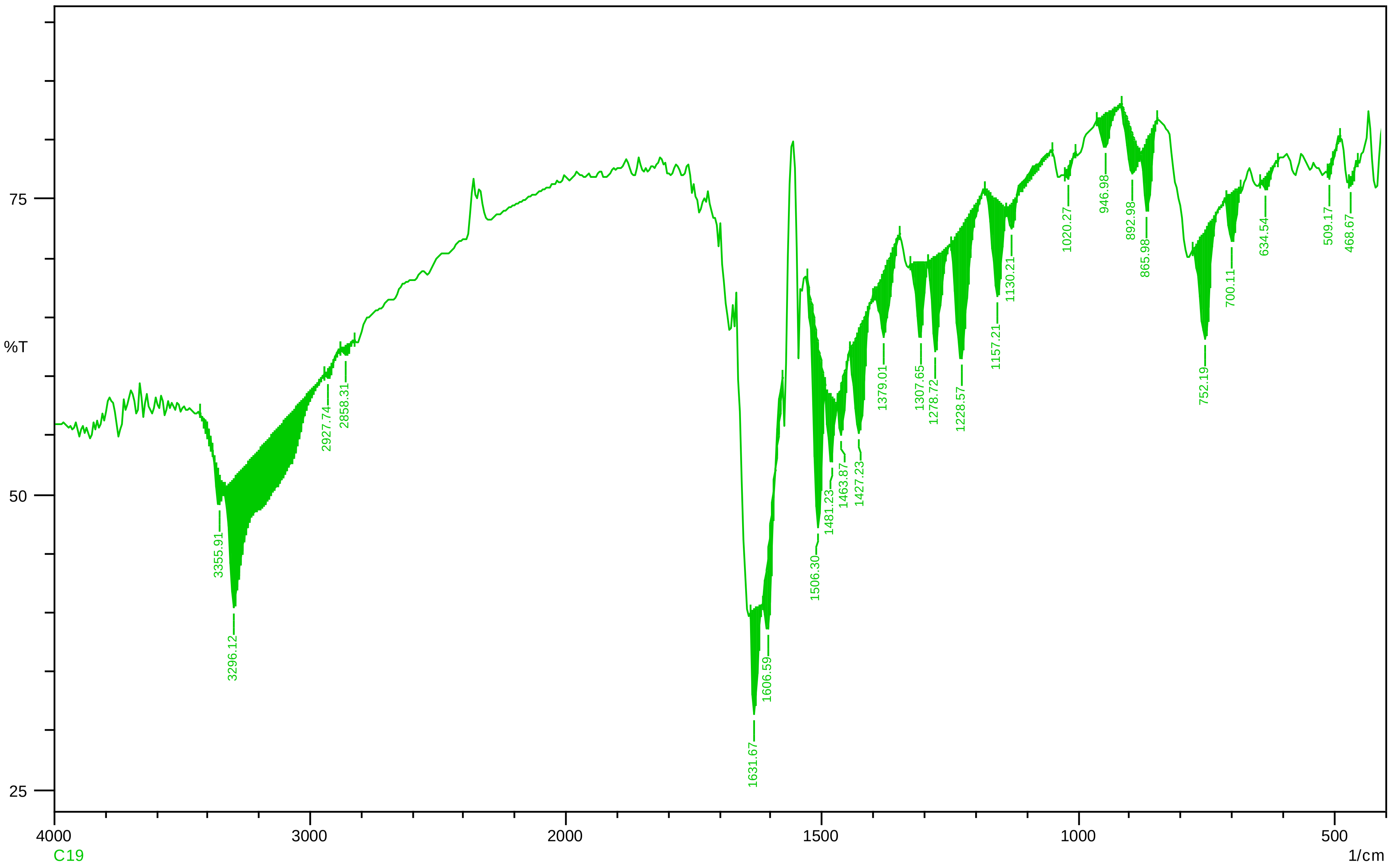

%T
75
50
25
4000
3000
2000
1500
1000
500
1/cm
C19
3355.91
3296.12
2927.74
2858.31
1631.67
1606.59
1506.30
1481.23
1463.87
1427.23
1379.01
1307.65
1278.72
1228.57
1157.21
1130.21
1020.27
946.98
892.98
865.98
752.19
700.11
634.54
509.17
468.67

PROTON DMSO {C:\Bruker\TOPSPIN\KFUCCP} nmr 5

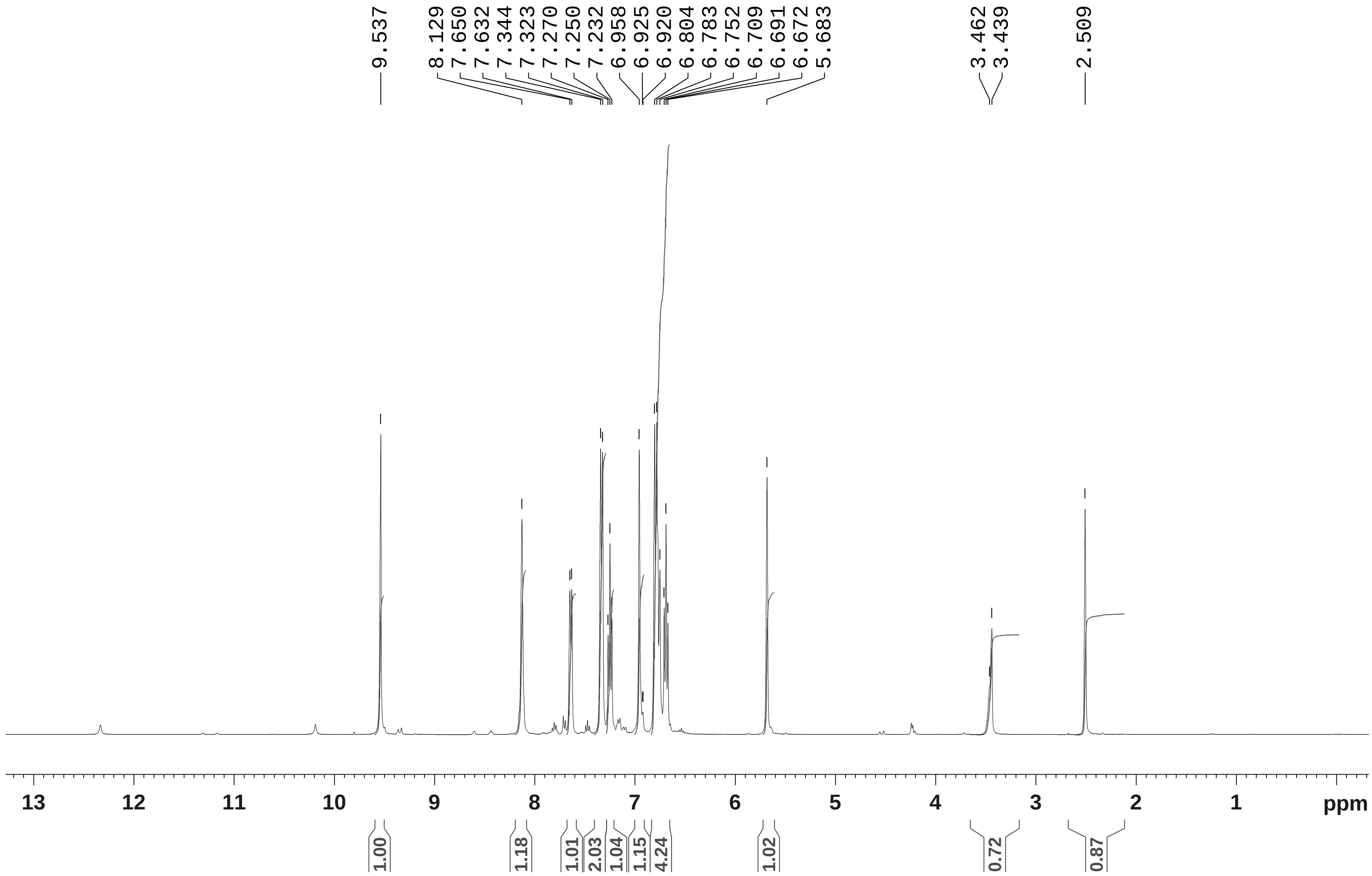

C13CPD DMSO {C:\Bruker\TOPSPIN\KFUCCP} nmr 5

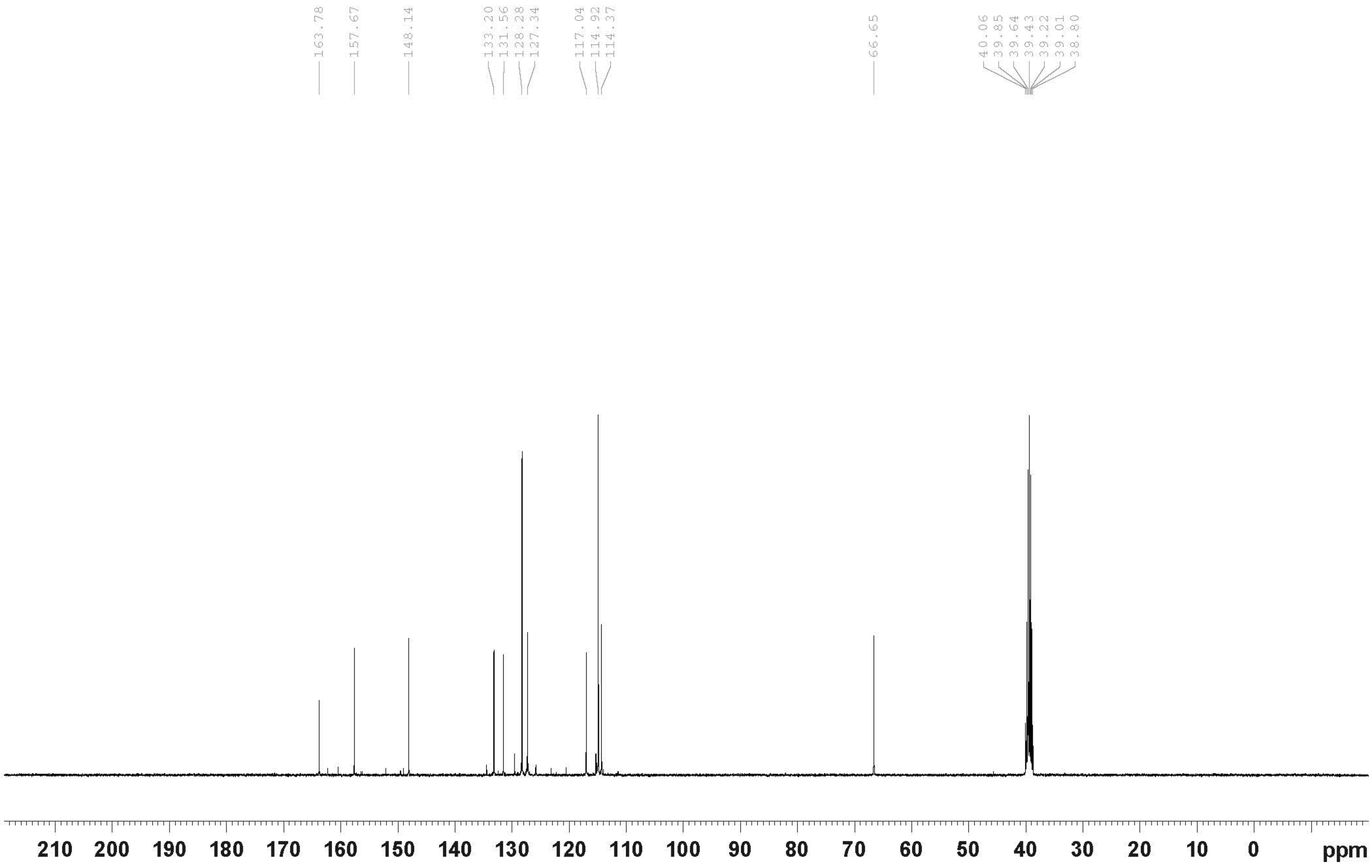

Supplement: Supplementary file 1 [file molecules-25-01316-s001.pdf]
